# Tetrabutoxy-substituted quintulene: A missing bowl-shaped cycloarene

**DOI:** 10.1126/sciadv.aee3923

**Published:** 2026-09-02

**Authors:** Mingxin He, Yuguang Sui, Jiawei Shao, Zipeng Wang, Yanwei Gu, Haiping Xia, Yong Ni

**Affiliations:** ^1^ Department of Chemistry, State Key Laboratory of Quantum Functional Materials, Southern University of Science and Technology, Shenzhen 518055, China.; ^2^ Guangdong Provincial Key Laboratory of Sustainable Biomimetic Materials and Green Energy, Southern University of Science and Technology, Shenzhen 518055, China.; ^3^ School of Chemistry and Chemical Engineering, Southeast University, Nanjing 211189, China.; ^4^ Materials Tech Laboratory for Hydrogen & Energy Storage, Ningbo Institute of Materials Technology & Engineering, Chinese Academy of Science, Ningbo, 315201, China.; ^5^ Shenzhen Grubbs Institute, Department of Chemistry, Southern University of Science and Technology, Shenzhen 518055, China.

## Abstract

The synthesis of corannulene and sumanene has pioneered the burgeoning field of buckybowl chemistry. Quintulene, a long-missing member of cycloarenes family, represents a distinctive type of bowl-shaped aromatics with curvature arising from edge topology and central cavity. Nevertheless, its synthesis has remained elusive for more than 40 years, primarily due to the high geometric strain. Herein we report the synthesis of tetrabutoxy-subtituted quintulene **1** through a rationally designed fold-in strategy. Single crystal analysis confirms the bowl-shaped geometry with a bowl-to-bowl inversion barrier of 19.1 kcal/mol. Quintulene **1** adopts a Clar-type localized aromatic character, consistent with other cycloarenes, but prone to hydrogenation and over-oxidation. Remarkably, the substantially structural strain attenuates local aromaticity in the benzenoid segments of quintulene **1**, enabling an almost quantitative fivefold Diels-Alder reaction. This work not only fills a critical gap in cycloarene chemistry but also underscores how geometric strain and electronic structure can reshape reactivity in curved π-systems.

## INTRODUCTION

**Fig. 1.**
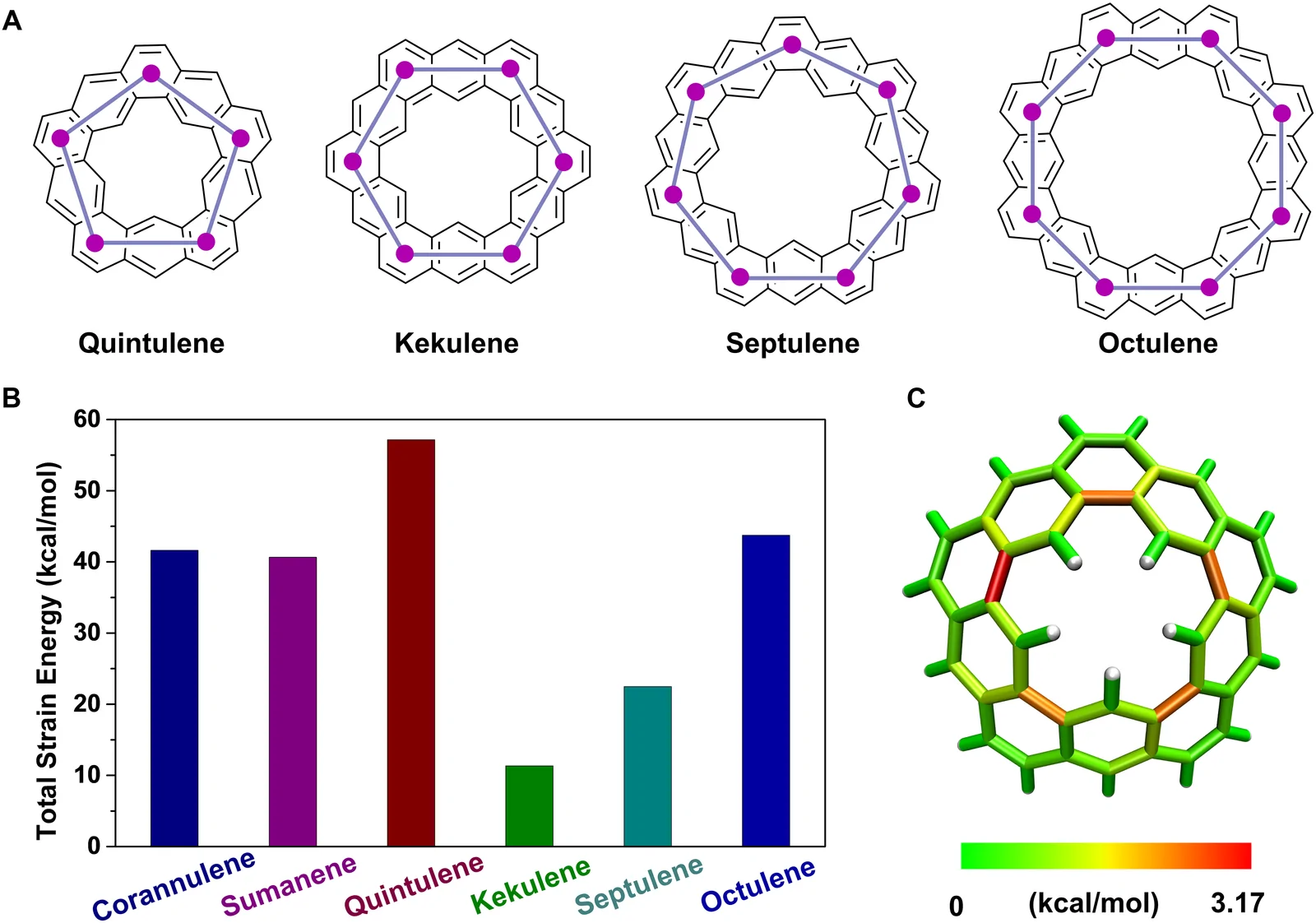
Representative cycloarenes and strain analysis. (**A**) Structures of representative cycloarenes with the centers of corner six-membered rings highlighted, the nomenclature derives from the polygonal shapes outlined by connecting these centers, namely pentagon for quintulene, hexagon for kekulene, heptagon for septulene and octagon for octulene. (**B**) Comparison of the total strain energies of quintulene to the conventional bowl-shaped polyarenes (corannulene and sumanene) and cycloarenes (calculated by B3LYP/def2TZVP). (**C**) Calculated Strain visualization map of quintulene.

In the landscape of carbon allotropes, graphene constitutes an atomically flat sheet (*1*), whereas fullerenes curve into closed spherical cages (*2*). Bridging these distinct dimensionalities are bowl-shaped polyarenes (*3*, *4*), their intrinsic curvature confers unique optoelectronic properties and bowl-to-bowl inversion dynamics (*5*, *6*), rendering them compelling candidates for organic electronics and supra-molecular chemistry (*7*). A long-standing paradigm for bowl-shaped aromatic compounds has been the reliance on pentagon embedding to induce positive Gaussian curvature, exemplified by the landmark structures of corannulene (*8*, *9*) and sumanene (*10*). Quintulene (*11*), a theoretically predicted yet synthetically elusive cycloarene consisting entirely of circularly-annulated benzenoid rings with radially oriented C-H bonds (*12*, *13*), challenge this convention, which also adopts a bowl-geometry (Fig. 1, A and C).

Cycloarenes have long intrigued chemists due to their electronic structures and topologies (*14*, *15*). Early interest centered on a fundamental question: whether their π-electrons follow Clar’s localized model (*16*) or exhibit global delocalization (*17*). This puzzle was conclusively addressed in 1978, when Staab and Diederich achieved the first synthesis and isolation of kekulene (*18*). X-ray crystallographic and ^1^H NMR studies, later corroborated by modern on-surface synthesis and scanning probe microscopy (*19*, *20*), confirmed a Clar-type localized aromatic character, an electronic feature shared by various homologs including contracted kekulene (*21*), septulene (*22*), octulene (*23*, *24*), and edge-expanded derivatives (*25*). The topologies of cycloarenes correlated directly with their central cavity. Kekulene’s hexagonal cavity yields planarity, while septulene’s heptagonal pore produces a mild negative curvature. Octulene displays a pronounced negative curvature and a hyperbolic geometry arising from the octagonal defect, which has been synthesized through a fold-in strategy (*23*) or a vinyl-ether cyclization protocol (*24*). Notably, quintulene stands apart with a positive curvature and bowl geometry that enabled by the edge topology and central pentagonal cavity.

Synthesis of bowl-shaped aromatic compounds represents a formidable challenge, primarily due to the high strain required to strategically fold a planar π-system into a stable concave architecture. Theoretical calculations predict a strain energy (*26*) of 57.17 kcal/mol for quintulene, substantially exceeding that of corannulene (41.6 kcal/mol) and sumanene (40.7 kcal/mol), and representing the highest value among cycloarene family (Fig. 1B and figs. S8 and S9). Therefore, the synthesis of parent quintulene remains elusive for decades, despite pioneering efforts by Staab and Sauer (*27*) since 1984 and the extended quintulene derivatives developed by Tan *et al.* recently (*11*, *28*). Herein, we report the first facile synthesis of butoxy-substituted quintulene and systematic investigation on its strain-governed properties. X-ray crystallographic and computational analysis demonstrate that butoxy-substituted quintulene maintains a Clar-type localized aromatic character with a non-negligible global π-electron delocalization, similar to other cycloarenes, but the local aromaticity within benzenoid segments is attenuated by the positive curvature and pronounced geometric strain. This electronic perturbation thereby facilitates an almost quantitative, fivefold Diels-Alder reaction, which underscores the profound relationship between geometric strain, electronic structure, and emergent reactivity in curved π-systems.

## RESULTS

### Molecular design and synthesis

Recent advances in molecular nanocarbons have provided powerful solutions to the long-standing challenge of constructing highly strained molecular nanocarbons (*29*–*31*). Among these, the fold-in strategy has emerged as a particularly effective approach. Stępień and colleagues pioneered this approach through inner-stitching of preorganized precursors achieved by McMurry or Wittig cyclooligomerization, enabling the synthesis of various strained bowl- and belt-shaped macrocycles (*32*–*34*). Itami’s group then developed a modified strategy that involves iterative Z-selective Wittig reaction, which can be used to construct a series of cylindrical and Möbius carbon nanobelts with extreme strain up to 119.5 kcal/mol (*35*–*37*). These methodological advances provide a robust foundation for targeting quintulene, whose geometrical strain is proposed primarily concentrated in the bonds along the periphery of central cavity that connect unshared benzene rings. Strain visualization mapping (*38*) (Fig. 1C) reveals a maximum bond strain of 3.27 kcal/mol, closely matching the reported value of Itami’s carbon nanobelt (3.07 kcal/mol).

**Fig. 2.**
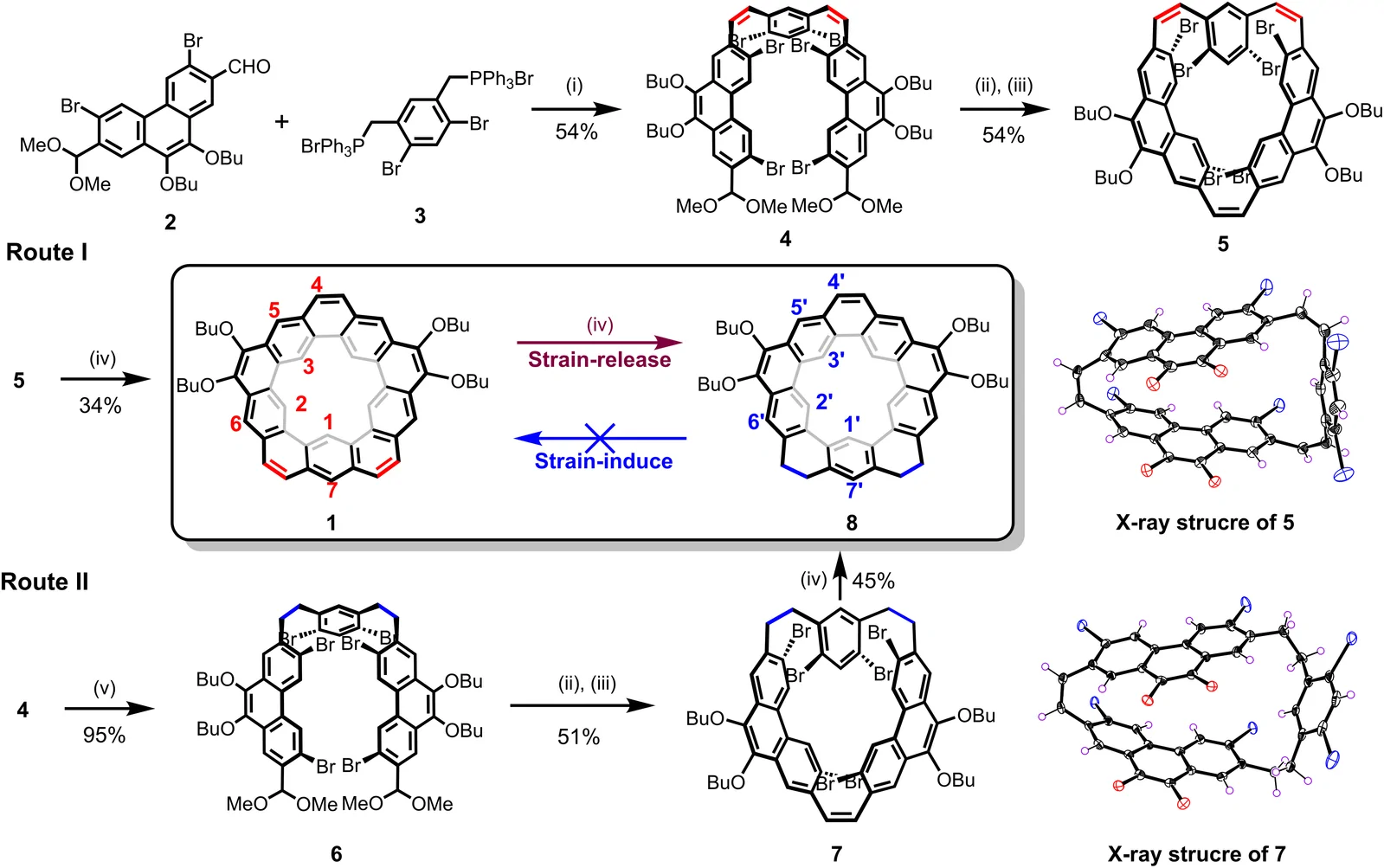
Synthetic routes to quintulene 1. Two strategies are employed based on a fold-in method. The inset shows the x-ray structure of **5** and **7** in Oka Ridge Thermal Ellipsoid plot (ORTEP), monochrome color-code are used, butyl-groups are omitted for clarity. Protons of **1** and **8** that discussed in the text labeled in colored numbers. Reagents and conditions: (i) NaOH(aq), DCM, 0°C to room temperature (r.t.); (ii) Trifluoroacetic acid (TFA), dichloromethane (DCM), r.t.; (iii) Zn, TiCl_4_, CuI, THF, reflux; (iv) Ni(cod)_2_, 2,2′-bipyridyl, THF, 65°C (reaction time: only **1** (2 hours); **1** and **8** (3 days); (v) 4-methylbenzenesulfonhydrazide (p-TsNHNH_2_), sodium acetate (CH_3_COONa), dioxane/H_2_O, reflux. OMe: methoxy-group; OBu: butoxy-group; cod: 1,4-cyclooctadiene.

Therefore, we designed a fold-in strategy for the synthesis of tetrabutoxy-substituted quitulene **1**, which involves Z-selective Wittig reaction followed by intramolecular McMurry macrocyclization to construct preorganized precursor, and culminates in a Yamamoto coupling to forge the most strained central bonds (Fig. 1E and Fig. 2, Route I). The synthesis commenced with asymmetrical dibromo-substitutued phenacene **2**, which was easily prepared in three steps from symmetrical dibromo-diiodo-substituted phenacene (see Supplementary Information). A two-fold Wittig reaction between **2** and dibrominated ylide reagent **3** produced **4** with high Z-selectivity due to the *ortho*-bromo effects, in 54% yield. **4** possess a symmetrical geometry, and the two Z-configured vinyl-segments generated a favorable spatial arrangement that would facilitate intramolecular macrocyclization. Acetal hydrolysis with trifluoroacetic acid (TFA), followed by intramolecular McMurry macrocyclization, furnished the preorganized all-Z macrocycle **5** in 54% yield over two steps, as confirmed by x-ray crystallography (Fig. 2 inset). Subsequent five-fold Yamamoto coupling of **5** using 10 equivalents of Ni(cod)_2_ and 2,2′-bipyridine at 65°C for 2 hours afforded tetrabutoxy-substituted quintulene **1** in 34% yield. **1** is partially oxidized on silica gel columns (fig. S47) even when deactivated with triethylamine (TEA), which can be purified by preparative gel permeation chromatography (GPC). It’s worth noting that prolonged reaction time (3 days) led to notable formation of partially hydrogenated product **8**, arising from strain-promoted nickel(0)-mediated reduction.

The unexpected strain-induced reduction inspired the design of an alternative synthetic strategy involving an early-stage hydrogenation of two olefinic bonds into ethylene units (Fig. 2, Route II). This structural modification potentially alleviates the cumulative strain (*25*, *39*) inherent in both the McMurry cyclization and Yamamoto coupling steps, thereby suppressing side reactions and enhancing the overall efficiency of the key stitching process. Diimide-mediated alkene hydrogenation of **4** proceeded quantitatively to afford **6**. Demethylation with TFA, and subsequent McMurry cyclization furnished the preorganized macrocycle **7** in 51% yield, comparable to that of **5**. The Z-configured vinyl group of **7**, unambiguously established by x-ray crystallography (Fig. 2 inset), enabled the intramolecular Yamamoto homocoupling and delivered **8** in 45% yield, slightly higher than the stitching of macrocycle **5**. However, all attempts to aromatize **8** to the fully conjugated quintulene **1** via oxidative dehydrogenation failed (table S1). A complex mixture of intractable substants was obtained even under mild conditions, attributing to the ease of over oxidation of quintulene **1** driven by the high geometric strain (fig. S47). The recurrence of unexpected reduction in the original route and over-oxidation in the alternative approach illuminate the long-standing synthetic challenge pose by quintulene and its analogues.

### Optical and electrochemical properties

Tetrabutoxy-substituted quintulene **1** exhibits a well-resolved absorption spectrum in dichloromethane, with a blue-shifted maximum (λ_abs_ = 317 nm, fig. S5A) relative to kekulene (λ_abs_ = 327 nm), consistent with its shortened π-conjugation pathway. The major absorption band with vibronic peaks at 296, 317, 350 nm arises from a combination of several high-lying occupied molecular orbitals HOMO−n to low-lying unoccupied molecular orbital LUMO+m (n, m = 2–5) electronic transtions according to time-dependent DFT calculations (table S2). A weak absorption tail extending from 380 to 500 nm is attributed to symmetry-forbidden transitions from HOMO-n to LUMO+m (n, m = 0–3), which was weakly allowed likely due to the lowered symmetry imposed by the butoxy substituents and dynamics of the bowl (fig. S10). The fluorescence spectrum of **9** shows well-resolved emission at 499 and 518 nm, the absolute fluorescence quantum yield was determined to be 2.11% by using integrating sphere technique. The relatively low quantum efficiency likely results from the weak absorption in the lower energy region. Cyclic voltammetry (CV) measurement of **1** in dichoromethane reveals multiple quasi-reversible redox process (fig. S5B), which can be further distinguished by differential pulse voltammetry (DPV) into three oxidation waves with half-wave potentials (E_1/2_^ox^) at 0.58, 0.74 and 1.01 V (vs Fc^+^/Fc), respectively.

### Molecular geometry and electronic structure

**Fig. 3.**
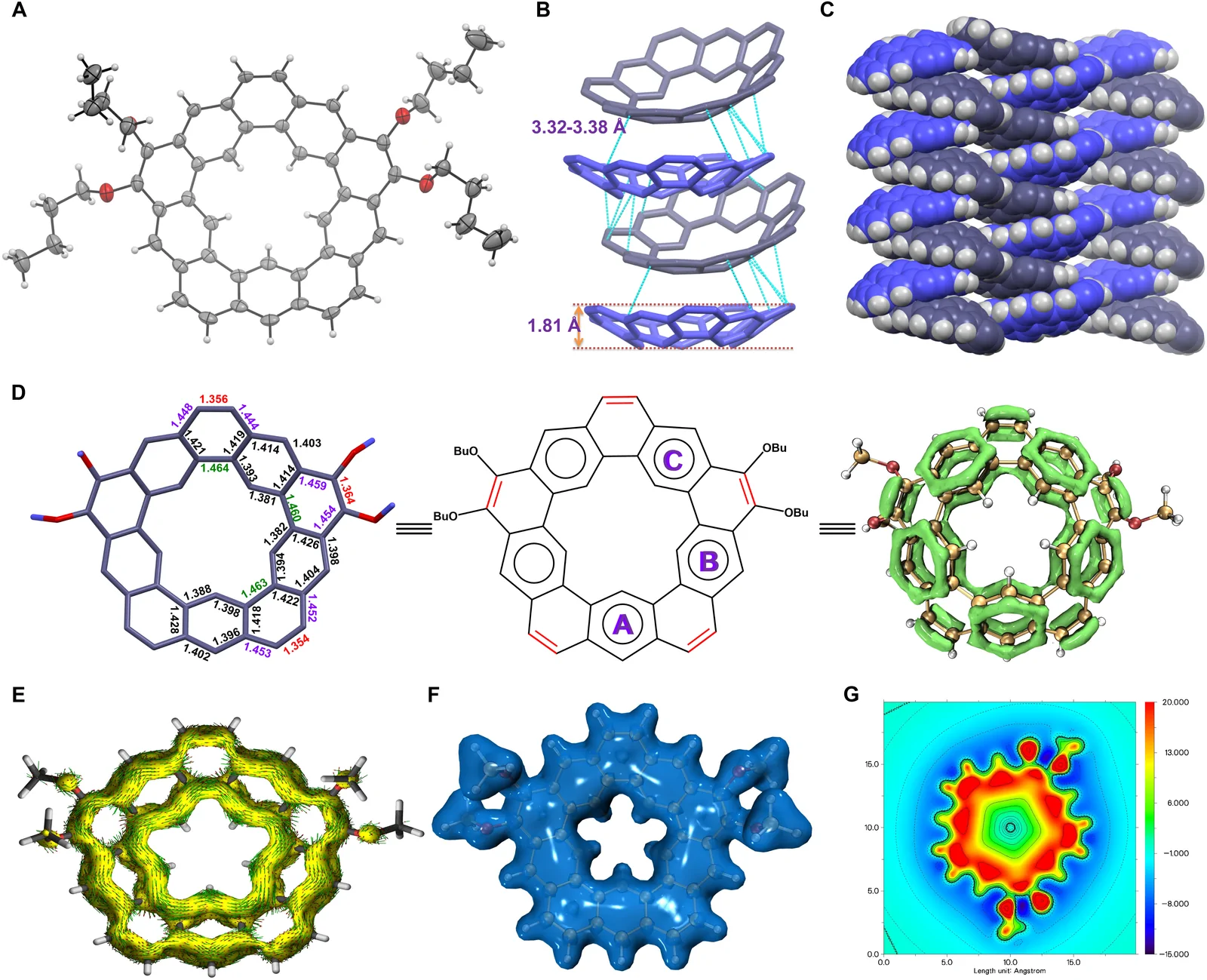
X-ray crystallographic structure and predominant electronic structure of 1. (**A**) Crystal structure of **1** in depicted as Oak Ridge thermal ellipsoid plots (ORTEP) at 50% probability from top view. (**B**) Capped-stick models for the conjugated skeletons of four packed molecules, with bowl-depth and multiple close π-π interactions between neighboring bowls highlighted. (**C**) Crystal packing as a space-filling model along c axis with butoxy-substituents omitted for clearly. (**D**) Selected bond lengths (in Å) from the x-ray structures (left, different colors correspond to distinct types of CC bonds), Clar’s localized bonding model (middle) and calculated localized orbital locator isosurface of π-orbitals (LOL-π, isovalue = 0.48, right) of quintulene. (**E**) Calculated ACID plots of **1** with an isovalue of 0.04, the arrows indicate the ring current flow. (**F**) Calculated 3D ICSS maps of **1** with an isovalue of 6. (**G**) 2D ICSS_zz_ map for the outer ring is provided to better clarify the shielding/deshielding area of the bowl. Butoxy-substituents were replaced by methoxy-groups to during calculations and the magnetic field is oriented perpendicular to the XY plane, pointing out through the paper.

Single crystals of **1** suitable for x-ray diffraction were grown by slow diffusion of methanol into its solution in chloroform (CHCl_3_) at room temperature. The butoxy-substituted quintulene can be understood as a curved hybrid of phenanthrene-like and anthracene-like motifs, the framework comprises five phenanthrene-like tricyclic sectors, each with a formal vinyl bond in the central ring. X-ray crystallographic analysis (Fig. 3, A to C and fig. S22) reveals a bowl-shaped geometry with evenly distributed curvature over the constituent rings. The measured bowl depth is 1.81 Å (Fig. 3B), substantially exceed that of corannulene (0.87 Å) (*40*). The molecules assemble into a slipped column through concave-to-convex (bowl-to-bowl) stacking, with a discernible offset between adjacent bowls. Multiple intermolecular close π-π contacts with distances in range of 3.32–3.38 Å were observed. Comprehensive bond length analysis reveals four distinct types of C − C bonds within the π-framework of **1** (Fig. 3D). The outer periphery contains five nearly equivalent C − C bonds within 1.354–1.376 Å, consistent with typical olefinic double-bond (∼1.35 Å). The C − C bonds linking these “double bonds” are much longer (1.436–1.459 Å), characterized as typical C(sp^2^)-C(sp^2^) single-bond. Bonds within ring A, B and C fall in the range of 1.381–1.428 Å, typical of aromatic benzene rings. Additionally, the bonds along the inner periphery that linking these unshared benzenoid rings (1.460–1.464 Å) also feature as typical C(sp^2^)-C(sp^2^) single-bond. These results suggest a dominant Clar-type aromatic model (Fig. 3D middle), wherein π-electrons mainly delocalized in five discrete benzene rings.

The harmonic oscillator measure of aromaticity (HOMA) (*41*) values for those six-membered rings containing olefinic double bonds was 0.27, 0.28 and 0.29 regarding to their non-aromatic character (fig. S14A). In contrast, rings A, B and C exhibit notably higher HOMA values (0.83), consistent with their localized aromaticity. The calculated localized orbital locator isosurface of π-orbitals (LOL-π) (*42*) clearly visualize the electronic structure of **1**, revealing five distinct benzene rings and five olefinic double bonds, in agreement with the bond-length-based assignments (Fig. 3D). In addition, the calculated two-dimensional isochemical shielding surface (ICSS) (*43*) map shows a magnetically deshielded chemical enviroment (negtive value) in the inner cavity and outside the periphery (Fig. 3G), while the three-dimensional ICSS map reveals that the shielding sphere mainly locates on the bowl’s backbone (Fig. 3F), again indicating a dominant local aromatic feature in quintulene.

Large negative nucleus independent chemical shift NICS(1)zz (*44*) values were predicted for the five aromatic sextet rings regarding to their localized aromaticity, whereas the other six-membered rings containing olefinic double bonds also showed notably negative NICS value indicating non-negligible contribution from the globally delocalized electronic structure (fig. S14A). Besides, the NICS(1)zz values of the five aromatic were less negative than those of phenanthrene (−29.2) and anthracene (−34.8) at the same position. Anistropy of the induced current density (ACID) (*45*) plots of **1** exhibits diatropic ring current circuit along the outer periphery while paratropic for the inner rim, and nearly zero current density along the radical bonds (Fig. 3E and fig. S12). This pattern can be rationalized by the superposition of diatropic ring currents associated with individual aromatic benzene rings and the cancellation effect along the radial bonds connecting inner and out rims. Nevertheless, obvious current density was observed across the CC double bonds, and a clockwise ring current circuit was formed along the outmost periphery, indicating that π-electrons within these olefinic segments remain capable of delocalizing over the macrocyclic framework, and the molecules were dominant with local aromaticity accompanied by a non-negligible degree of global (super)aromaticity (*46*), similar to kekulene and its expanded homologs. In particular, quintulene exhibit a more pronounced electron delocalization along its inner periphery relative to the outer rim (Fig. 3E), owing to its bowl-shaped topology and substantial structural strain (fig. S13).

### Bowl-to-bowl dynamics

**Fig. 4.**
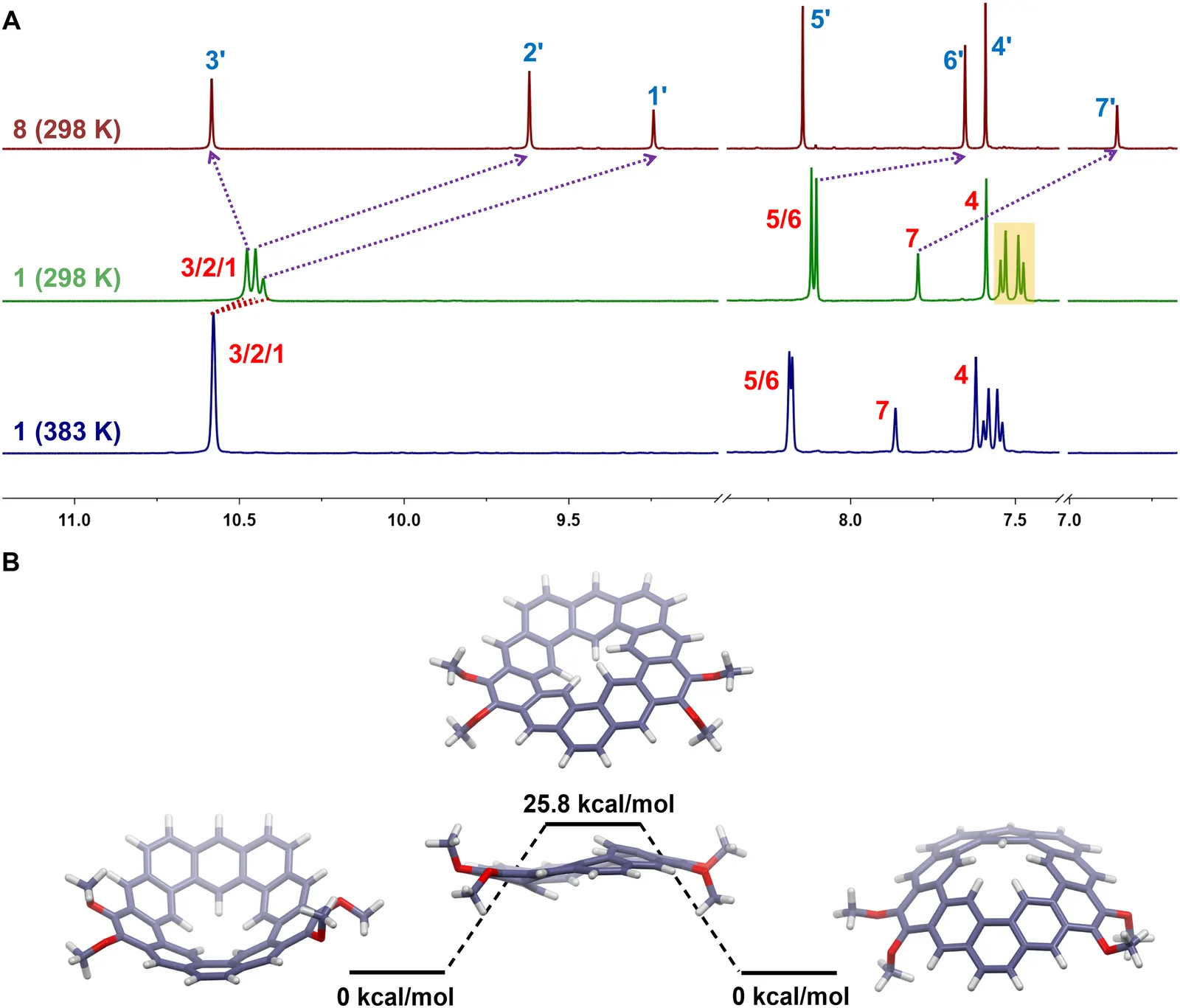
^1^H-NMR spectra and bow-to-bow dynamics. (**A**) Spectra of **8** in CDCl_3_ at 298 K and **1** in C_2_D_2_Cl_4_ at 298 K and 383 K. Molecular structures are shown in Fig. 2 with labeled assignments. Resonance peaks associated with protons on two symmetric olefinic double bonds in **1** are highlighted, which disappeared in **8**, reflecting the hydrogenation motivated by nickel(0) during the synthesis, the shift of protons at the central benzenoid rings are also highlighted to support the formation of saturated ethylene subunits. (**B**) The proposed pathway for the flip-flop (bowl-to-bowl) dynamics of quintulene **1** with butoxy-groups simplified to methoxy-groups to reduce computational cost. A clip-shaped transition state and an interconversion energy barrier of 25.6 kcal/mol are predicted at room temperature. The side-view and top view of the transition state are present to clarify its clip-shaped geometry.

The ^1^H NMR spectra of quintulene **1** in C_2_D_2_Cl_4_ at 298 K was well-resolved and fully assigned (Fig. 4A). Both the inner- and outer-rim protons are deshielded with resonances appeared in the low-field region, further supporting a localized aromatic character of quintulene **1**. The inner protons, sharing similar chemical environment, give rise to three slightly separated singlets (Δν ≈ 50 Hz) near 10.5 ppm in a 1:2:2 ratio, which gradually merge into a time-averaged singlet (Fig. 4A, and figs. S1 and S2) upon heating, indicating rapid bowl-to-bowl inversion over NMR timescale at elevated temperatures. Variable-temperature (VT) NMR studies revealed a coalescence temperature of 383 K, corresponding to an estimated inversion barrier of approximately 19.1 kcal/mol (*47*). Theoretical calculations (B3LYP/def2TZVP) support a flip-flop inversion mechanism via a clip-shaped transition state, an energy barrier of 25.6 kcal/mol was predicted (Fig. 4B), in reasonable agreement with the experimental value. The concentration dependent fluorescence (fig. S6) and ^1^H-NMR (fig. S7) measurements confirmed notable aggregation at high concentrations, consistent with the offset bowl-to-bowl stacking that observed in single crystal structure.

### Geometric strain governed reactivity

**Fig. 5.**
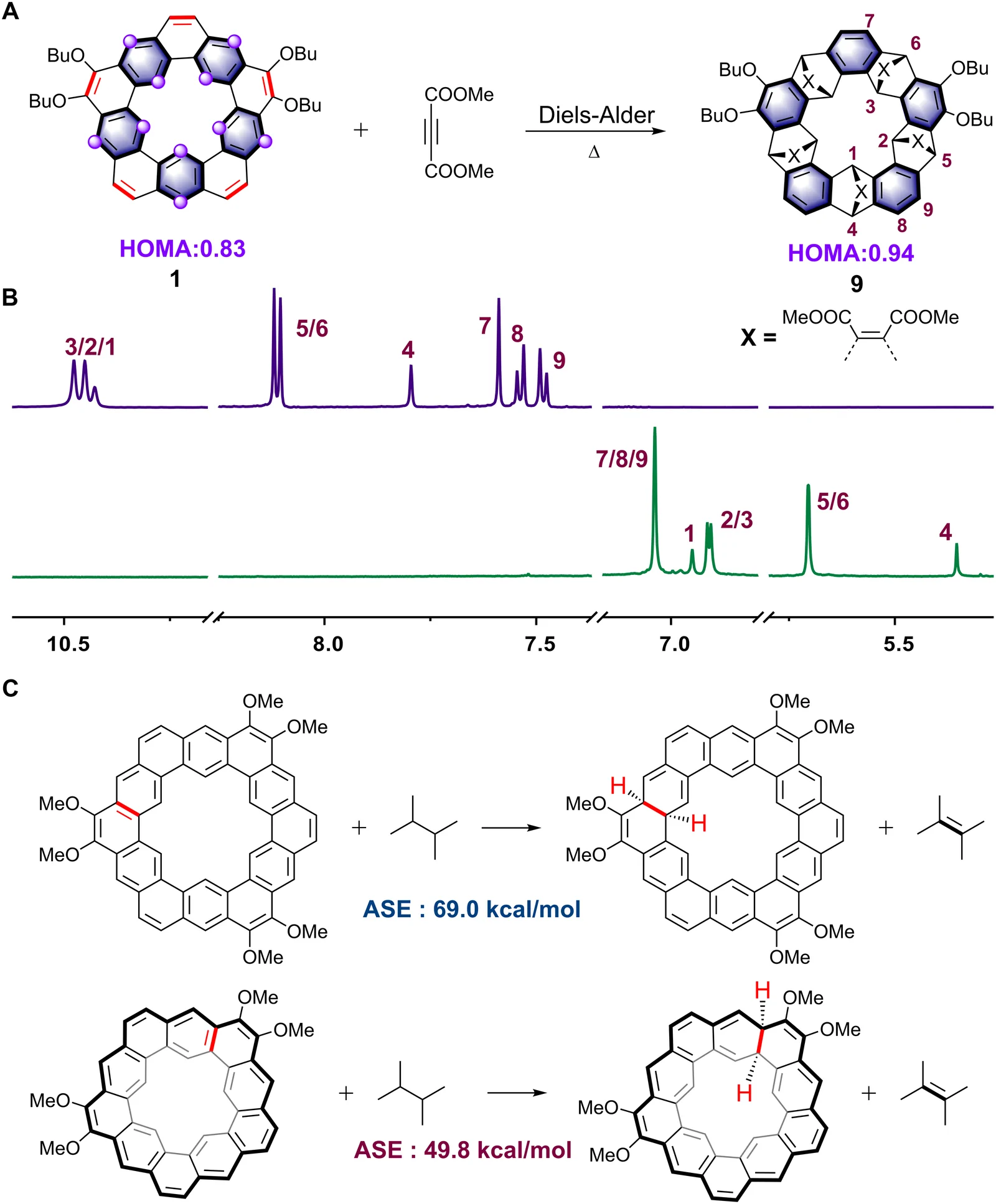
Geometric strain governed Reactivity. (**A**) Five-fold Diels-Alder reaction of butoxy-subtituted quintulene **1** with reactive sites highlighted and aromatic benzenoid rings shaded. The HOMA value of the benzenoid rings in both **1** and **9** are highlighted for comparison. (**B**) ^1^H NMR spectra of **1** and **9** with the assignment of protons at same carbon-sites. (**C**) Calculated aromatic stabilization energy (ASE) of the localized benzenoid rings in both kekulene and quintulene **1** [B3LYP/6-31G(d,p)].

The bowl-shaped curvature and pronounced strain of quintulene is expected to elongate the C-C bonds at the “rim” while compressing those along the “hub”, the central benzenoid rings are thereby activated which could exhibit reactivity similar to that of anthracene. To test this hypothesis, quintulene **1** was subjected to Diels-Alder reaction with dimethyl acetylenedicarboxylate, leading to a fivefold cycloaddition (Fig. 5, A and B). While the Diels–Alder reaction of anthracene with DMAD requires a high temperature of 145°C (*48*), the same cycloaddition proceeds at 105°C for our bowl-shaped system, reflecting a moderate activation effect originating from geometric strain. DFT calculations (B3LYP/def2TZVP) identified isomer **9** (fig. S15), with all five DMAD units attached in the same outward orientation, as the lowest-energy structure, consistent with the NMR data. The substantially released strain and notably higher HOMA values for the benzenoid rings of **9** (0.93–0.95) compared to pristine quintulene **1** (0.83), drives the Diels-Alder reaction. In contrast, planar kekulene remains robust under identical condition (*49*). To further elucidate this geometric-strain governed reactivity, the averaged aroamtic stabilization energy (ASE) of the central benzenoid rings of butoxy-substituted quintulene was estimated as 34.7, 50.7 and 28.3 kcal/mol based on the hydrolysis of the outer, linking and inner double bond respectively, which is substantially lower than that of kekulene (41.0, 69.0 and 38.9 kcal/mol, Fig. 5C and figs. S16, S17, and S18), suggesting that curvature-induced strain attenuates local aromaticity, imparting amplified triene character to the central rings and thereby favoring efficient Diels-Alder cycloaddition.

## DISCUSSION

In summary, a butoxy-substituted quintulene was synthesized through a fold-in strategy involving Z-selective Wittig reaction followed by intramolecular McMurry macrocylization, and culminating in a Yamamoto coupling for the stitching process. The bowl-shaped geometry was unambiguously confirmed x-ray single crystal analysis, and the flip-flop (bowl-to-bowl) dynamics was monitored by VT-NMR with an estimated inversion barrier of 19.1 kcal/mol through a clip-shaped transition state. Comprehensive structural and computational analysis confirmed a Clar-type localized aromatic character. Quintulene is peculiarly prone to hydrogenation and over-oxidation during preparation. In particular, the pronounced curvature and geometric strain diminished the ASE remarkably, imparting amplified triene character to the central rings of quintulene, thus enabling an almost quantitative fivefold Diels-Alder reaction. The synthesis of butoxy-substituted quintulene fills a long-standing gap in the cycloarene family and opens avenues to other strained π-systems with nontrival topologies, such as twisted cycloarenes. Moreover, this work underscores how strain and curvature unlock unprecedented transformation pathways in curved π-systems, which may expand the synthetic arsenal toward functional carbon-rich architectures.

## MATERIALS AND METHODS

### General experimental procedures

All reactions were performed under a nitrogen atmosphere, inflame-dried glassware with magnetic stirring using standard Schlenk techniques, unless otherwise mentioned. All solvents were distilled over CaH_2_ or Na and purged with nitrogen before use. All other reagents were purchased from commercial sources and used without purification, unless otherwise noted. Thin-layer chromatography was performed on commerical silica gel and visualized by a ultra-violet lamp (254 nm). ^1^H nuclear magnetic resonance (NMR) and ^13^C NMR were recorded at room temperature unless other-wise required on a Bruker Avance 400 MHz or a Bruker Avance 600 MHz spectrometer.

Crystallographic data have been deposited with the Cambridge Crystallographic Data Centre as supplementary publication. X-ray single crystal data were collected on a Bruker D8 Venture diffractometer using Ga (Mo) Kα radiation source. The selected crystal was kept at 100.0 (200.0) K during data collection. Using Olex2 (*50*), the structure was solved with the ShelXT (*51*) structure solution program using Intrinsic Phasing and refined with the ShelXL (*52*) refinement package using Least Squares minimization.

### Compound synthesis

Methods for compound synthesis are described in supplementary materials. **S0** and **3** were synthesized according to literature procedures (*23*, *53*).

**S0**: To an argon-purged solution of 3,6-Dibromo-2,7-diiodo-phenanthrene-9,10-dione (3.00 g, 4.86 mmol) in tetrahydrofurane (180 mL) and water (120 mL), were added tetra-n-butylammonium bromide (0.47 g, 1.46 mmol) and sodium hydrosulfite (3.38 g, 19.4 mmol). After 20 minutes of stirring a solution of potassium hydroxide (2.72 g, 46.6 mmol) in water (30 mL) was added to the reaction mixture followed by 1-bromobutane (2.62 mL, 24.3 mmol). The red colored reaction mixture was stirred under a reflux condenser in 110°C overnight. After cooling most of the tetrahydrofurane was removed on a rotary evaportator, resulting in precipitation of the crude product from the reaction mixture, which was then filtered under reduced pressure, washed with water and dried in air, subsequent column chromatography gave **S0**.

**3**: To a stirring solution of 1,5-dibromo-2,4-bis(bromomethyl)benzene (4.00 g, 9.48 mmol) in DMF (20 mL) in a 50-mL 2-necked flask equipped with reflux condenser under nitrogen was added triphenylphosphine (6.22 g, 23.7 mmol) in a single portion. The mixture was heated at reflux with continued stirring for 20 hours. After cooling to room temperature, the mixture was diluted with an additional 75 mL of DMF and poured into 300 mL stirring toluene. The precipitated solids were filtered off and washed with toluene and Et_2_O. The solid material was collected and dried under high vacuum to provide **3**.

### Theoretical calculations

Density functional theory (DFT) calculations on the geometrical and electronic properties of the ground-state were performed at the B3LYP/6-31G(d,p) level and the energy calculation was performed at the B3LYP/def2TZVP (*54*). The bowl-to-bowl inversion dynamics were searched using the quadratic synchronous transit (OST3) method at the B3LYP/6-31G(d,p) theory level (*55*). The transition state (TS) was found to exhibit only one imaginary frequency. The Intrinsic Reaction Coordinate (IRC) calculation succeeded by fully geometry optimization (pseudo-IRC) demonstrates that the transition state follows the minimum energy path downhill to the product and reactant. Time-dependent DFT (TD-DFT) calculations were performed at the B3LYP/6-31G(d,p) level of theory under vacuum. NICS values were calculated [B3LYP/6-31G(d,p)] using the standard GIAO (GIAO=NMR) (*45*). ACID plot [B3LYP/6-31G(d,p)] was calculated by using the method developed by Herges based on the optimized ground-state geometries based on the x-ray crystal structure (*44*). To devoid σ-bond effect, the additional option [NMR = CSGT, IOp(10/93) = 2] was used to read in wanted p-orbitals (*56*). The iso-chemical shielding surface (ICSS) calculations were carried out and the VMD programme was used to plot ICSS cube files (*43*, *57*, *58*).

## Supplementary Materials

### The PDF file includes:

Supplementary Text Tables S1 to S5 Figs. S1 to S47 Cartesian coordinates of optimized structures Legends for data S1 to S3
