# Supplementary material for: Tetrabutoxy-substituted quintulene: A missing bowl-shaped cycloarene
Source: Sci Adv. 2026 Sep 2;12(36):eaee3923. doi: 10.1126/sciadv.aee3923 (PMC13537259; doi:10.1126/sciadv.aee3923)
Supplement: Supplementary file 1 [file sciadv.aee3923_sm.pdf]

Science Advances
AAAS

Supplementary Materials for

Tetrabutoxy-substituted quintulene: A missing bowl-shaped cycloarene

Mingxin He *et al.*

Corresponding author: Yong Ni, niy@sustech.edu.cn

Sci. Adv. **12**, eaee3923 (2026)
DOI: 10.1126/sciadv.aee3923

The PDF file includes:

Supplementary Text
Tables S1 to S5
Figs. S1 to S47
Cartesian coordinates of optimized structures
Legends for data S1 to S3

Other Supplementary Material for this manuscript includes the following:

Data S1 to S3

1. Materials and methods

All reactions were performed under an argon atmosphere using standard Schlenk techniques with dry solvents and oven-dried glassware, unless otherwise specified. Commercially available reagents were used as received. ^{1}H NMR and ^{13}C NMR spectra were acquired on Bruker AVANCE 400 or 600 MHz spectrometers. Chemical shifts for ^{1}H NMR chemical shifts are reported in parts per million (ppm) relative to residual chloroform (δ 7.26 ppm) or 1,1,2,2-tetrachloroethane (δ 6.00 ppm). High-resolution mass spectrometry (HRMS) was performed on a Thermo Fisher Scientific Q-Exactive mass spectrometer. UV-Vis-NIR spectra were recorded on a PerkinElmer Lambda 750s spectrophotometer. Absolute photoluminescence quantum yields (PLQY) were determined using an Edinburgh Instruments FLS1000 spectrophotometer. Electrochemical measurements, including cyclic voltammetry (CV) and differential pulse voltammetry (DPV), were carried out on a Zahner IM6e electrochemical workstation. A standard three-electrode configuration was employed, consisting of a glassy carbon working electrode, a platinum wire counter electrode, and an Ag/AgCl reference electrode. Measurements were conducted in a dichloromethane solution containing 0.1 M tetrabutylammonium hexafluorophosphate ($TBAPF_6$) as the supporting electrolyte, with ferrocene added as an internal standard. Purification by preparative recycling gel permeation chromatography (GPC) was conducted using a Japan Analytical Industry Recycling Preparative HPLC (LacoACE LC-5060 PlusII) with THF as the eluent.

2. Synthetic procedures

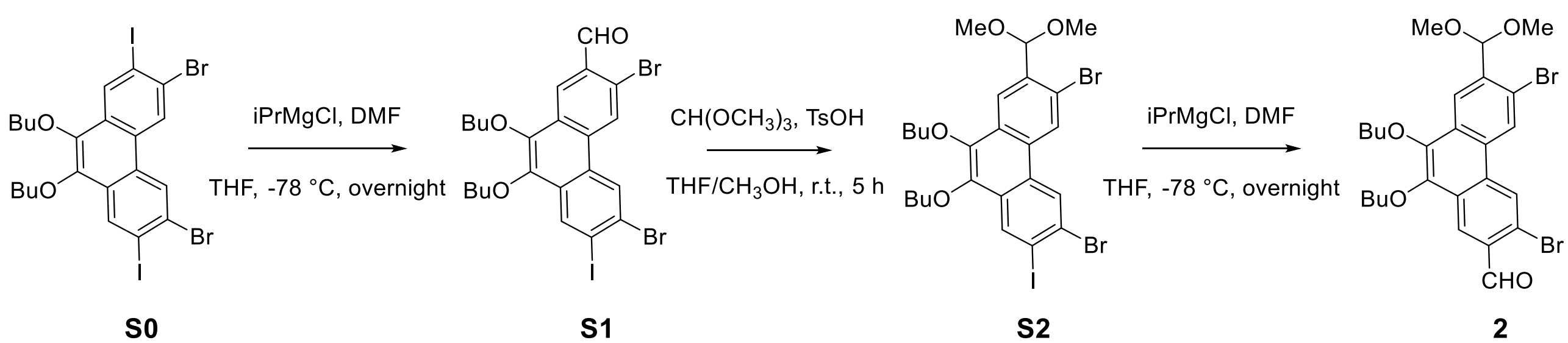

Synthesis of 2:

S0 (7.32 g, 10 mmol) was dissolved in dry THF (100 mL) and the solution was cooled to -78 °C, and iPrMgCl (2.0 M in THF, 5 mL, 10 mmol) was added dropwise over 30 mins. After stirring at -78 °C for 1 h, anhydrous DMF (3.0 mL, 20 mmol) was added. The reaction mixture was then allowed to warm to room temperature and stirred overnight. The reaction was quenched with saturated aqueous NH_4Cl solution (50 mL). The aqueous layer was extracted with dichloromethane (DCM), and the combined organic layer were dried over Na_2SO_4 and concentrated under reduced pressure. The resulting crude product, containing **S1**, was used directly in the next step without further purification.

The crude product containing **S1** was dissolved in a mixture of THF (40 mL), DCM (40 mL), and methanol (4 mL). To this solution were added $CH(OCH_3)_3$ (3.0 mL, 26.3 mmol) and p-toluenesulfonic acid (172 mg, 1.0 mmol). The resulting mixture was stirred at room temperature for 10 hours. Upon completion, the reaction was quenched with a saturated aqueous $NaHCO_3$ (10 mL). The mixture was concentrated under reduced pressure, and the residue was roughly purified by a quick column chromatography on silica gel (petroleum ether). The crude product containing **S2** was used in the next step.

The crude product containing d **S2** was dissolved in dry THF (60 mL) under an argon atmosphere. The solution was cooled to -78 °C, and iPrMgCl (2.0 M in THF, 5 mL, 10 mmol) was added dropwise. After stirring at -78 °C for 1 hour, anhydrous DMF (3.0 mL, 20 mmol) was added. The reaction mixture was warmed to room temperature and stirred overnight. The reaction was quenched by the addition of saturated aqueous NH_4Cl solution (20 mL). The mixture was extracted with dichloromethane, and the combined organic layers were dried over Na_2SO_4 and concentrated under reduced pressure. The crude residue was purified by flash column chromatography on silica gel (petroleum ether/DCM, 20:1 v/v) to afford **2** as a yellow solid(2.50 g, 43%). ^{1}H NMR (400 MHz, $CDCl_3$, 25 °C): δ ppm 10.52 (s, 1H), 8.78 (s, 1H), 8.74 (s, 1H), 8.72 (s, 1H), 8.50 (s, 1H), 5.72 (s, 1H), 4.22 (t, J = 6.6 Hz, 4H), 3.46 (s, 6H), 1.92-1.83 (m, 4H), 1.66-1.55 (m, 4H), 1.02 (t, J = 7.3 Hz, 6H). ^{13}C NMR (100 MHz, $CDCl_3$): δ ppm 191.77, 144.17, 143.72, 136.89, 131.90, 131.71, 130.35, 129.55, 128.15, 128.07, 128.00, 125.88, 122.99, 122.17, 121.19, 102.79, 73.88, 73.71, 54.05, 32.52, 32.51, 19.52, 19.49, 14.08, 14.05. HRMS analysis (APCI, m/z) calcd. for $C_{26}H_{31}Br_2O_5$ $[M+H]^+$: 581.0533, found: 581.0537.

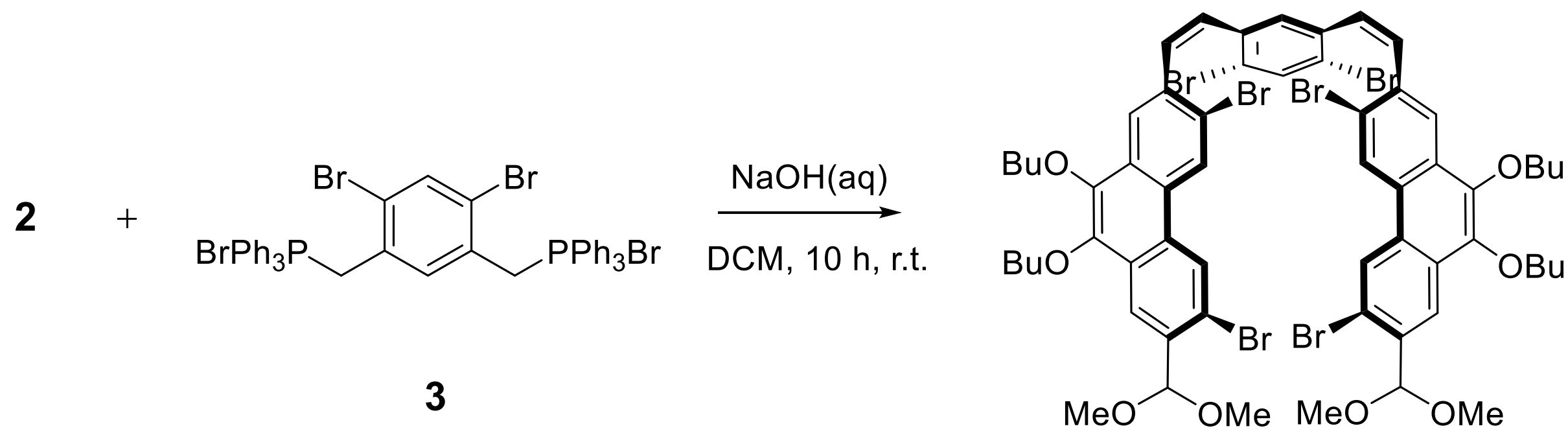

3

4

Synthesis of 4:

A mixture of **2** (0.75 g, 1.29 mmol) and **3** (0.40 g, 0.59 mmol) was dissolved in dry dichloromethane (40 mL) under an argon atmosphere. To this solution was added an aqueous sodium hydroxide solution (1.0 M, 2.75 mL). The reaction mixture was stirred in the dark for 10 hours. Upon completion, the reaction was quenched with saturated aqueous ammonium chloride solution and extracted with dichloromethane. The combined organic extracts were dried over anhydrous Na_2SO_4 and concentrated under reduced pressure. The crude residue was purified by column chromatography (petroleum ether/ethyl acetate = 100:1, v/v) to afford **4** as a faint yellow solid (0.45 g, 54%). ^{1}H NMR (400 MHz, $CDCl_3$, 25 °C): δ ppm 8.76 (s, 2H), 8.50 (s, 2H), 8.47 (s, 2H), 7.84 (s, 1H), 7.76 (s, 2H), 7.02 (s, 1H), 6.55 (d, J = 12.1 Hz, 2H), 6.44 (d, J = 12.1 Hz, 2H), 5.77 (s, 2H), 4.17 (t, J = 6.5 Hz, 4H), 3.79 (t, J = 6.6 Hz, 4H), 3.48 (s, 12H), 1.87-1.80 (m, 4H), 1.64-1.50 (m, 8H), 1.42-1.25 (m, 4H), 0.98 (t, 12H). ^{13}C NMR (100 MHz, $CDCl_3$): δ ppm 143.79, 143.24, 136.53, 135.41, 135.17, 134.99, 131.78, 130.90, 129.11, 128.99, 128.79, 128.77, 127.94, 127.15, 127.04, 123.88, 123.63, 122.85, 121.53, 120.96, 102.95, 73.66, 73.64, 53.98, 32.54, 32.40, 19.52, 19.43, 14.17, 14.07. HRMS analysis (APCI, m/z) calcd. for $C_{60}H_{64}Br_6O_8Na$ $[M+Na]^+$: 1408.9594, found: 1408.9466.

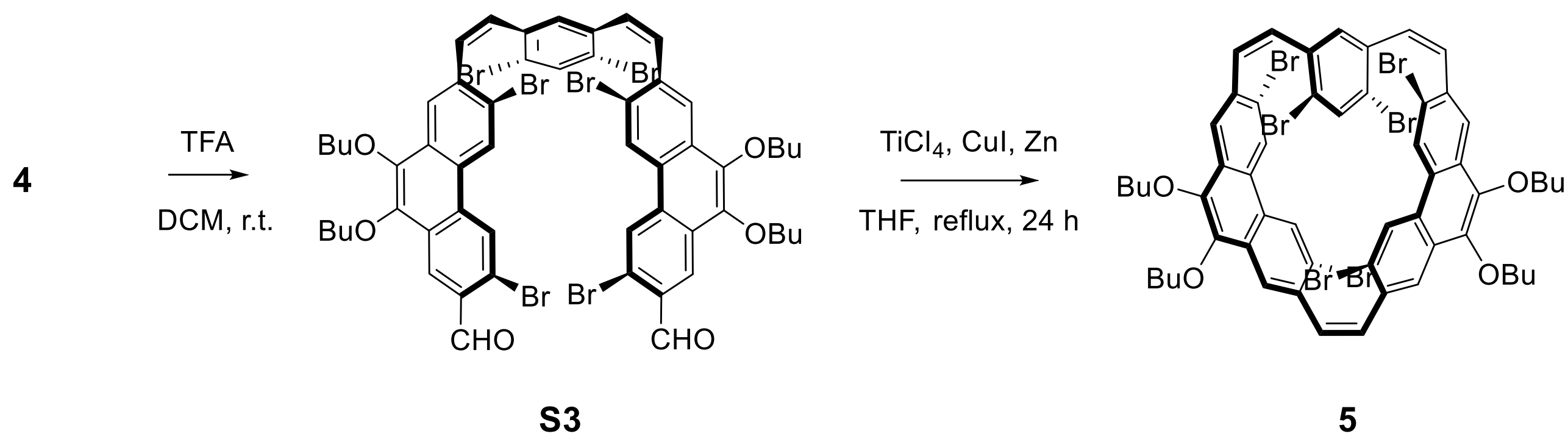

Synthesis of 5:

Trifluoroacetic acid (TFA) (0.5 mL, 6.40 mmol) was added to a solution of **4** (0.45 g, 0.32 mmol) in dichloromethane (DCM) at 0 °C. The resulting mixture was warmed to room temperature and stirred for 12 hours. The reaction was cautiously quenched by the successive addition of cold water and a saturated aqueous $NaHCO_3$ solution. The mixture was extracted with DCM, and the combined organic layers were dried over anhydrous Na_2SO_4 and concentrated under reduced pressure. The crude residue was triturated with methanol to afford the crude product containing **S3**, which was used directly in the next step without further purification.

A mixture of zinc powder (2.27 g, 34.88 mmol) and copper(I) iodide (250 mg, 1.32 mmol) in dry THF (350 mL) was cooled to 0 °C under an argon atmosphere. Titanium(IV) chloride (1.9 mL, 17.56 mmol) was added dropwise, and the resulting mixture was heated under reflux for 2 hours. A solution of the crude **S3** (300 mg, 0.23 mmol) in dry THF (60 mL) was then added to the refluxing mixture over 5 hours via a syringe pump. After the addition was complete, the reaction was maintained under reflux for an additional 24 hours. The mixture was cooled to room temperature, quenched by the addition of $NaHCO_3$, concentrated under reduced pressure and the residue was rapidly purified by flash column chromatography on silica gel (dichloromethane as eluent). Further purification by preparative gel permeation chromatography (GPC) using THF as the eluent at a flow rate of 16 mL/min afforded **5** (172 mg, 54%) as faint yellow solid. ^{1}H NMR (400 MHz, $CDCl_3$, 25 °C): δ ppm 8.12 (s, 2H), 8.01 (s, 2H), 7.92 (s, 1H), 7.47 (s, 2H), 7.46 (s, 2H), 7.32 (s, 2H), 6.92 (s, 1H), 6.80 (d, *J* = 11.8 Hz, 2H), 6.48 (d, *J* = 11.9 Hz, 2H), 4.17-4.03 (m, 4H), 3.59 (m, 2H), 3.23 (m, 2H), 1.79 (m, 4H), 1.74 (m, 4H), 1.54 (m, 8H), 1.05 (m, 12H). ^{13}C NMR (100 MHz, $CDCl_3$): δ ppm 142.78, 142.11, 138.84, 135.92, 135.76, 135.23, 134.94, 131.56, 131.27, 130.25, 127.97, 127.19, 127.00, 126.60, 125.73, 125.00, 123.45, 123.32, 121.27, 73.28, 73.25, 32.87, 32.75, 19.72, 19.57, 14.43, 14.21. HRMS analysis (APCI, m/z) calcd. for $C_{56}H_{53}Br_6O_4$ $[M+H]^+$: 1262.9039, found: 1262.9058.

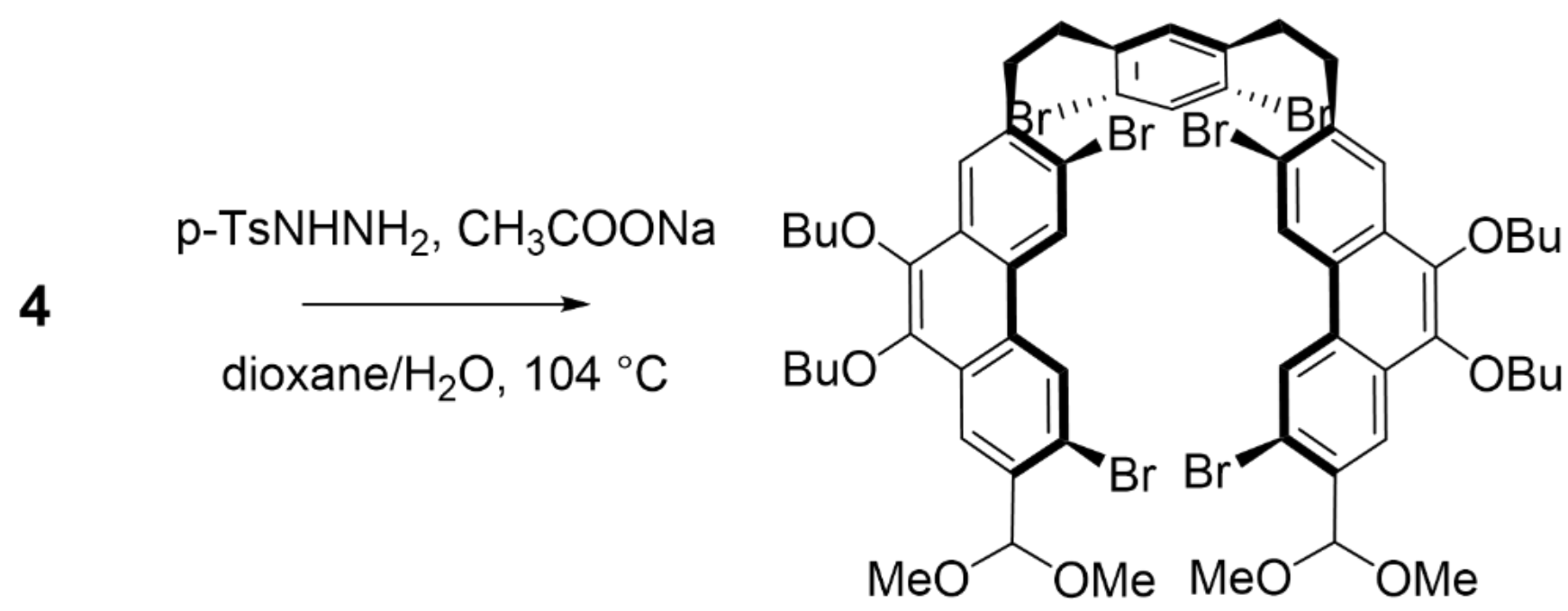

6

Synthesis of 6:
A mixture of 4 (600 mg, 0.43 mmol), 4-methylbenzenesulfonhydrazide (p-$TsNHNH_2$, 800 mg, 4.3 mmol), and sodium acetate (CH_3COONa, 352 mg, 4.3 mmol) in dioxane (20 mL) and water (5 mL) was stirred under an argon atmosphere at 110 °C for 7 days. After cooling to room temperature, the mixture was concentrated under reduced pressure. The residue was partitioned between dichloromethane (DCM) and water. The organic layer was separated, dried over anhydrous Na_2SO_4 and concentrated. The resulting crude material was washed with methanol to afford **6** (575 mg, 95%). ^1H NMR (400 MHz, $CDCl_3$, 25 °C): δ ppm 8.64 (s, 2H), 8.63 (s, 2H), 8.37 (s, 2H), 7.77 (s, 2H), 7.76 (s, 1H), 6.74 (s, 1H), 5.60 (s, 2H), 4.14 (t, J = 6.6 Hz, 4H), 4.07 (t, J = 6.4 Hz, 4H), 3.36 (s, 12H), 3.01-2.87 (m, 8H), 1.79-1.86 (m, 8H), 1.49-1.65 (m, 8H), 1.04 (t, J = 7.4 Hz, 6H), 0.98 (t, J = 7.4 Hz, 6H). ^{13}C NMR (100 MHz, $CDCl_3$): δ ppm 143.65, 143.14, 139.84, 139.12, 135.91, 134.98, 132.72, 129.46, 128.64, 128.61, 127.18, 127.08, 123.95, 122.88, 122.64, 122.33, 120.70, 102.77, 73.60, 73.40, 53.81, 36.50, 35.85, 32.69, 32.56, 29.85, 19.71, 19.51, 14.24, 14.06.

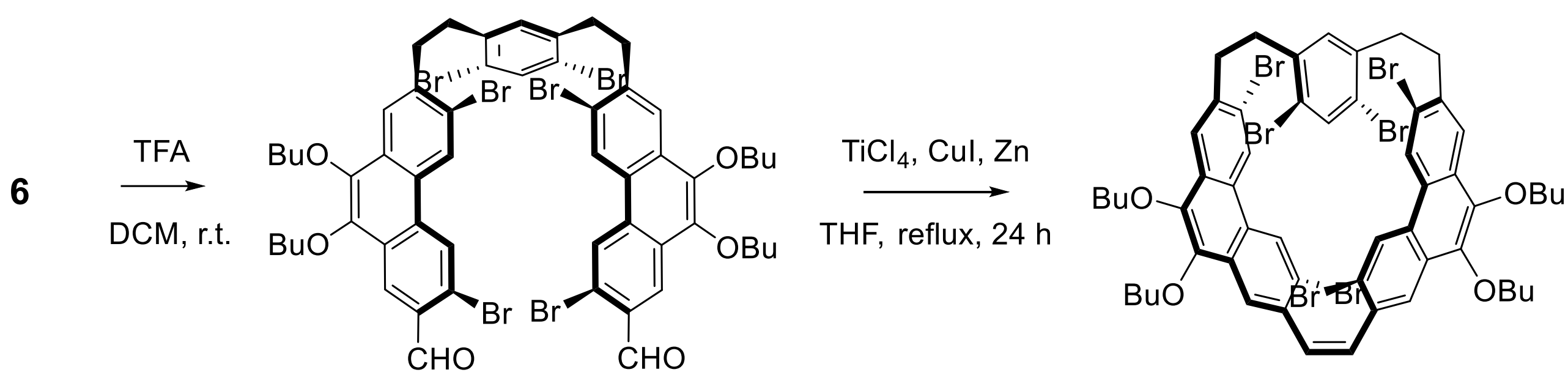

S4 **7**

Synthesis of 7:
To a solution of **6** (0.45 g, 0.32 mmol) in DCM at 0 °C was added trifluoroacetic acid (TFA) (0.50 mL, 6.40 mmol) and the resulting mixture was stirred at room temperature for 12 h. The reaction was quenched by adding cold water and aq.$NaHCO_3$ The organic layer was extracted with DCM and then evaporate in vacuo to afford crude product containing **S4** which was used in the next step without further purification.
To a mixture of zinc (2.30 g, 45.3 mmol) and copper(I) iodide (290 mg, 1.5 mmol) in dry THF (350 mL) was added titanium tetrachloride (2.5 mL, 22.8 mmol) slowly at 0 °C under Ar atmosphere and the reaction mixture was heated to reflux for 2 h. A solution of **S4** (398 mg, 0.31 mmol) in dry THF (100 mL) was added to the boiling mixture using a syringe pump. After completion of addition (5 hours), the mixture was kept under reflux for 24 h. After cooling to the

room temperature, the mixture was quenched by the addition of $NaHCO_3$, the solvent was removed under the reduced pressure and the residue was quickly purified by silica gel column chromatography (DCM), and then further purified by preparative GPC using THF at a rate of 16 mL/min to afford **7** (197 mg, 51%) as white solid. ^{1}H NMR (600 MHz, $CDCl_3$, 25 °C): δ ppm 8.09 (s, 2H), 8.01 (s, 2H), 7.86 (s, 1H), 7.47 (s, 2H), 7.41 (s, 2H), 7.30 (s, 2H), 6.84 (s, 1H), 4.08 (m, 4H), 3.66-3.56 (m, 2H), 3.44 (m, 2H), 3.35-3.03 (m, 8H), 1.83-1.74 (m, 4H), 1.65-1.48 (m, 12H), 1.06 (t, *J* = 7.1 Hz, 6H), 1.01 (t, *J* = 7.4 Hz, 6H). ^{13}C NMR (100 MHz, $CDCl_3$): δ ppm 142.72, 141.96, 139.12, 137.92, 136.52, 135.68, 135.62, 130.66, 128.22, 126.99, 126.73, 126.43, 126.35, 126.33, 124.96, 123.26, 122.76, 122.14, 121.34, 73.24, 73.00, 34.29, 32.71, 32.68, 32.39, 19.66, 19.56, 14.33, 14.19. HRMS analysis (APCI, m/z) calcd. for $C_{56}H_{57}Br_6O_4$ $[M+H]^+$: 1266.9352, found: 1266.9266.

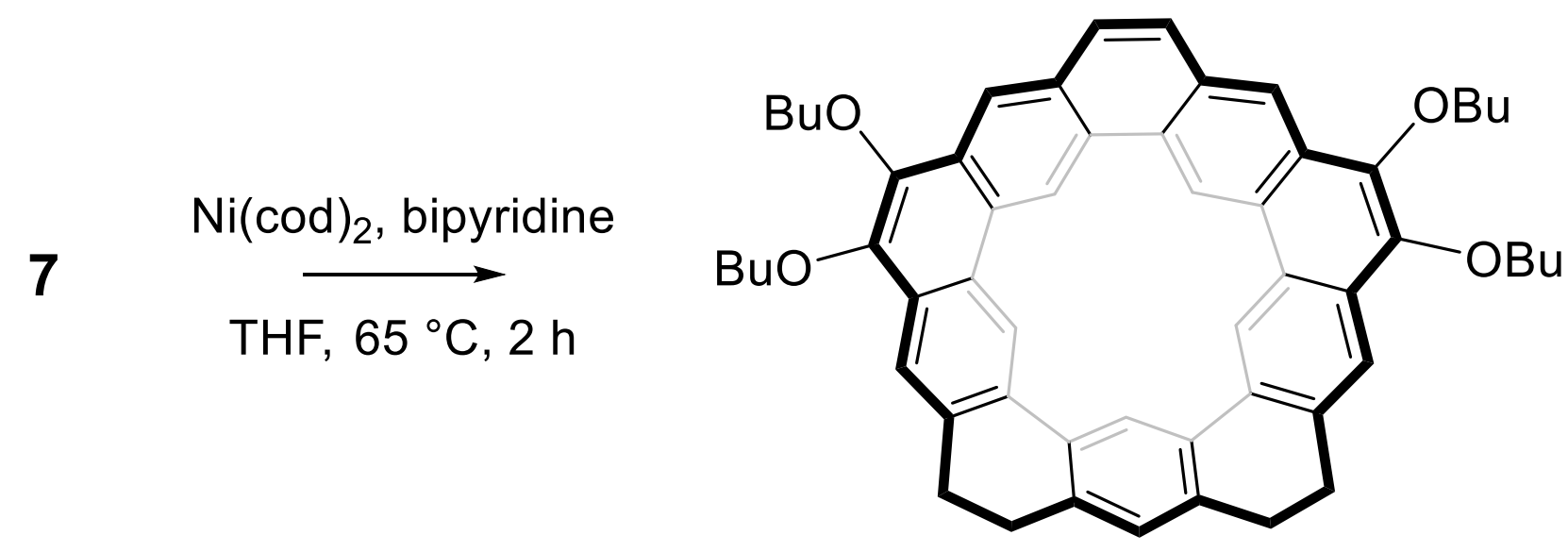

8

Synthesis of 8:

A mixture of **7** (120 mg, 0.094 mmol), bis(1,5-cyclooctadiene)nickel(0) (Ni(cod)$_2$, 260 mg, 0.95 mmol), and 2,2'-bipyridyl (150 mg, 0.96 mmol) in dry THF (60 mL) was prepared in a glovebox. The reaction mixture was heated at 65 °C for 2 hours with stirring. After cooling to room temperature, the solvent was removed under reduced pressure. The crude residue was initially purified by a short silica gel column (dichloromethane) followed by preparative gel permeation chromatography (GPC) using THF as the eluent at a flow rate of 16 mL/min, to afford **8** (34 mg, 45%) as yellow solid. ^{1}H NMR (600 MHz, $CDCl_3$, 25 °C): δ ppm 10.58 (s, 2H), 9.62 (s, 2H), 9.24 (s, 1H), 8.15 (s, 2H), 7.65 (s, 2H), 7.59 (s, 1H), 6.86 (s, 1H), 4.36 (dt, *J* = 9.5, 6.7 Hz, 2H), 4.31 (dt, *J* = 9.5, 6.7 Hz, 2H), 4.13 (dt, *J* = 9.5, 6.6 Hz, 2H), 4.06 (dt, *J* = 9.5, 6.6 Hz, 2H), 3.38-3.25 (m, 2H), 3.07-3.11 (m, 2H), 2.81-2,85 (m, 2H), 2.53 - 2.59 (m, 2H), 1.86-1.93 (m, 8H), 1.58-1.65 (m, 8H), 1.03 (td, *J* = 7.4, 2.9 Hz, 12H). ^{13}C NMR (100 MHz, $CDCl_3$): δ ppm 143.96, 143.25, 136.21, 135.53, 134.91, 130.96, 130.50, 129.53, 129.43, 129.34, 128.73, 127.89, 126.70, 126.12, 125.06, 122.13, 121.21, 121.19, 73.65, 73.60, 32.75, 32.72, 28.34, 27.94, 19.59, 19.58, 14.17. HRMS analysis (APCI, m/z) calcd. for $C_{56}H_{57}O_4$ $[M+H]^+$: 793.4251, found: 793.4243.

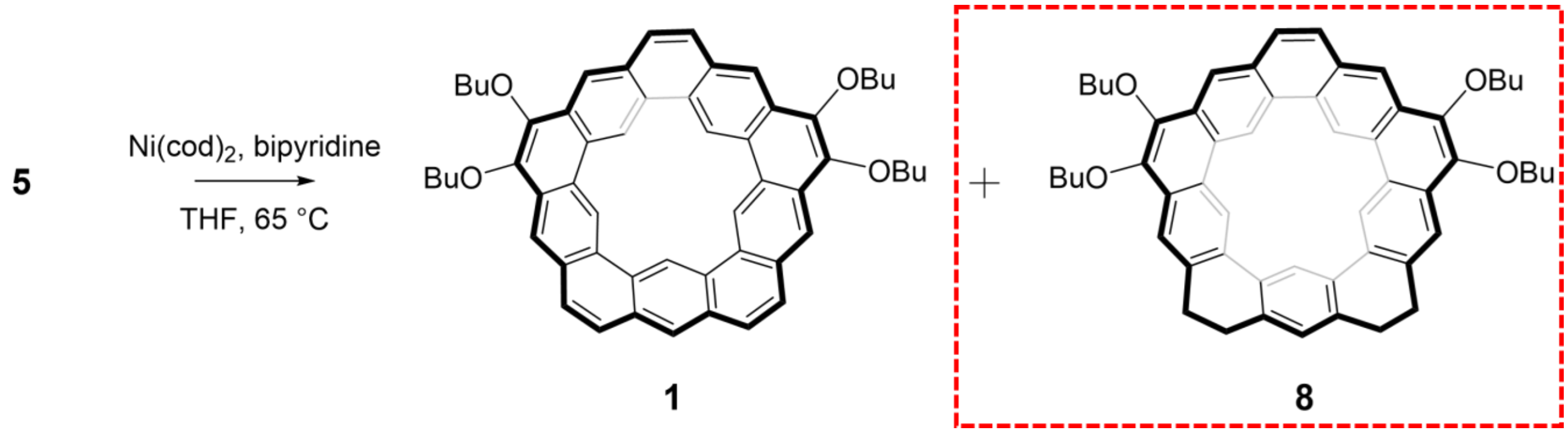

Synthesis of 1:

5 (202 mg, 0.15 mmol), bis(cyclooctadiene)nickel(0) (400 mg, 1.46 mmol) and 2,2'-bipyridyl (280 mg, 1.46 mmol) were dissolved in dry THF (100 mL) in a glovebox. The reaction mixture was stirred and heated to 65 °C for 2 h. After cooling to room temperature, the solvent was removed under vacuum, and the residue was first purified by a short flash column chromatography (DCM), and then further purified by preparative GPC using THF at a rate of 16 mL/min to afford **1** (40 mg, 34%) as yellow solid. Notably, **8** was isolated in about 5% yield following a longer reaction period of three days. ^{1}H NMR (600 MHz, $C_2D_2Cl_4$, 25 °C): δ ppm 10.48 (s, 2H), 10.45 (s, 2H), 10.43 (s, 1H), 8.12 (s, 2H), 8.10 (s, 2H), 7.80 (s, 1H), 7.59 (s, 2H), 7.54 (d, J = 9.2 Hz, 2H), 7.48 (d, J = 9.3 Hz, 2H), 4.41 (m, 4H), 4.13 (m, 4H), 1.93 (m, 8H), 1.62 (m, 8H), 1.06 (t, J = 7.4 Hz, 12H). ^{13}C NMR (100 MHz, $CDCl_3$): δ ppm 143.27, 143.25, 131.11, 131.03, 130.55, 130.50, 130.38, 129.57, 129.47, 129.28, 129.24, 126.81, 126.57, 126.46, 122.24, 122.08, 121.85, 121.81, 121.53, 73.53, 32.77, 19.60, 14.19. HRMS analysis (APCI, m/z) calcd. for $C_{56}H_{53}O_4$ $[M+H]^+$: 789.3938, found: 789.3946.

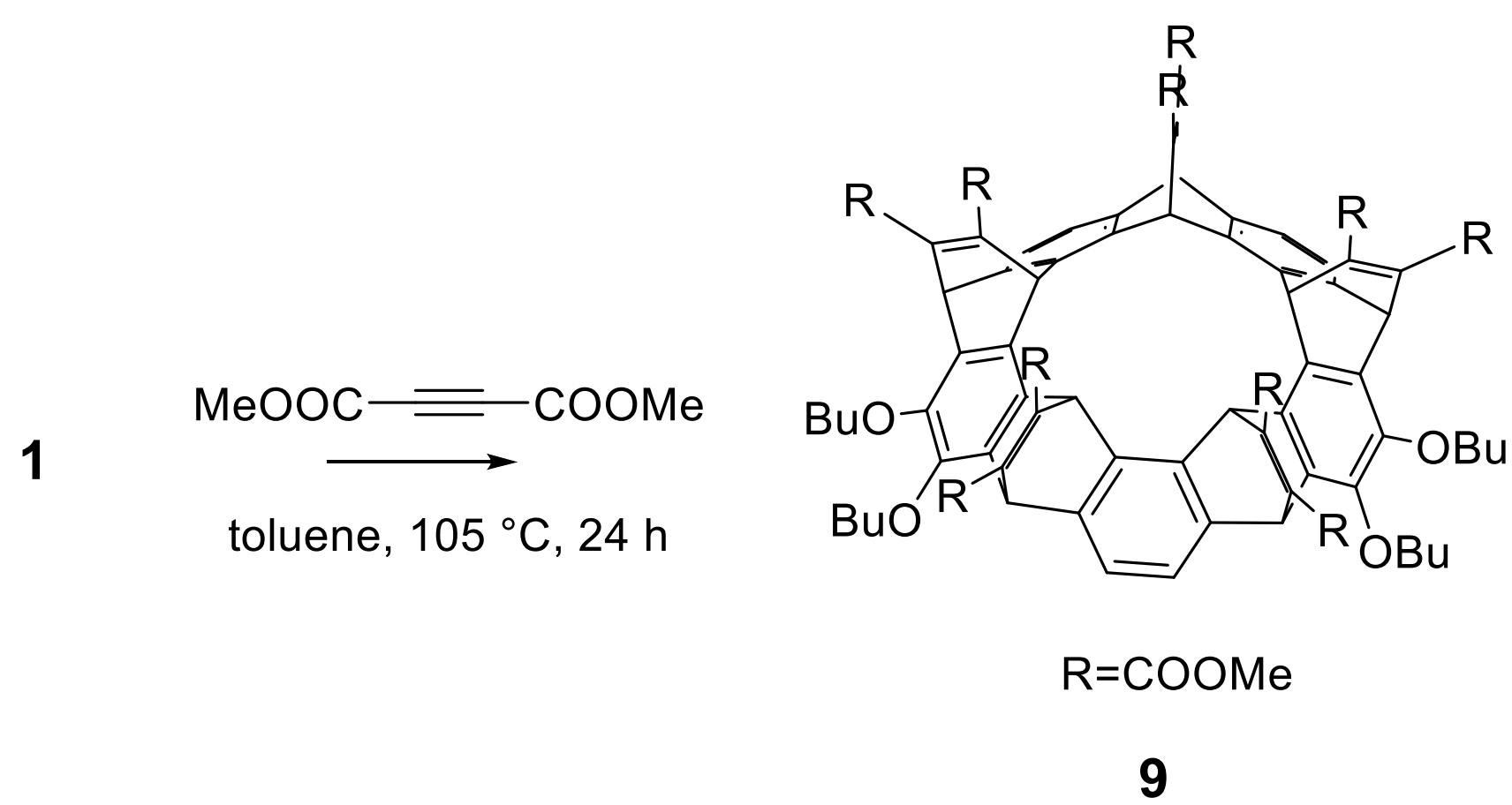

Synthesis of 9:

A solution of **1** (10 mg, 0.0127 mmol) and dimethyl acetylenedicarboxylate (DMAD) (0.19 mL, 12.6 mmol) in dry toluene (3 mL) was stirred at 105 °C for 24 h under N_2 atmosphere. The solvent was removed under a reduced pressure. The residue was recrystallized from a small amount of hexane to give pure **9** a as a solid (12 mg). ^{1}H NMR (400 MHz, $CDCl_3$, 25 °C): δ ppm 7.04 (s, 6H), 6.95 (s, 1H), 6.91 (s, 2H), 6.91 (s, 2H), 5.71 (s, 2H), 5.70 (s, 2H), 5.35 (s, 1H), 3.74-3.89 (m, 38H), 1.68-1.71 (m, 8H), 1.42-1.49 (m, 8H), 0.92-0.95 (t, J = 7.4 Hz, 12H). ^{13}C NMR (100 MHz, $CDCl_3$): δ ppm 166.00, 165.88, 165.65, 149.31, 149.28, 148.18, 145.56, 141.19, 141.06, 141.01,

138.91, 138.78, 138.07, 134.19, 133.80, 121.23, 121.05, 74.42, 52.53, 52.41, 47.43, 44.78, 32.44, 19.44, 14.10. HRMS analysis (APCI, m/z) calcd. for $C_{86}H_{83}O_{24}$ $[M+H]^+$: 1499.5269, found: 1499.5263.

Table S1. **Attempts of reactions for the oxidative aromatization of 8**.

Oxidants	Reaction conditions and results
DDQ	rt, overoxidation
p-chloranil	rt, no reaction; 80 °C overoxidation
Pd/C	150 °C, no reaction; 230 °C, partial decomposition
MnO_2	80 °C, no reaction
Br_2	0 °C, no formation of **1**
I_2	no reactiom

3. Additional spectra

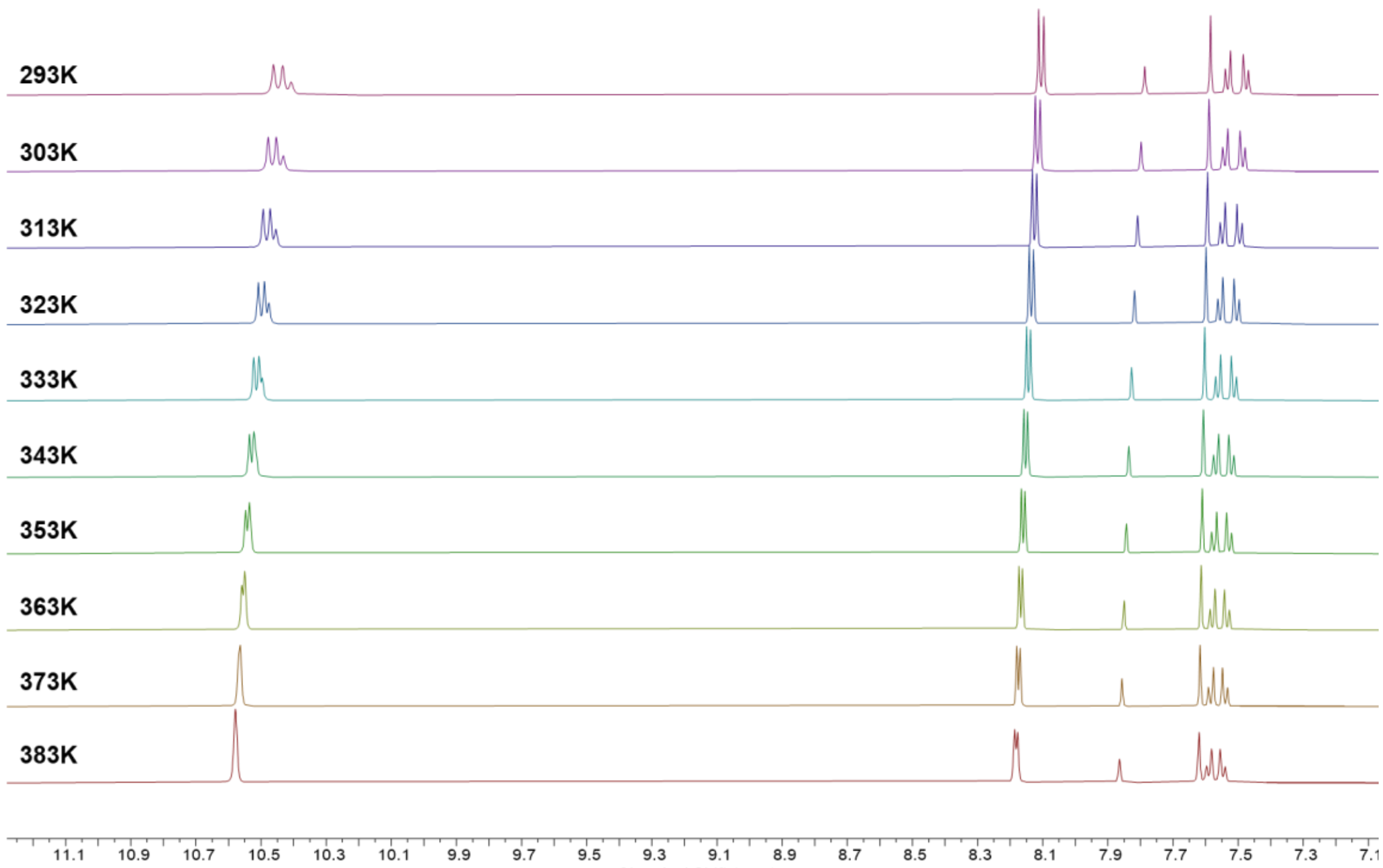

Fig. S1. VT ^{1}H NMR spectrum of 1. The spectrum of the aromatic region was recorded in the temperature ranging from 293K to 383K in $CDCl_2CDCl_2$ (600 MHz), the signals of inner protons gradually merged into a sharp singlet upon increasing the temperature to the 383K.

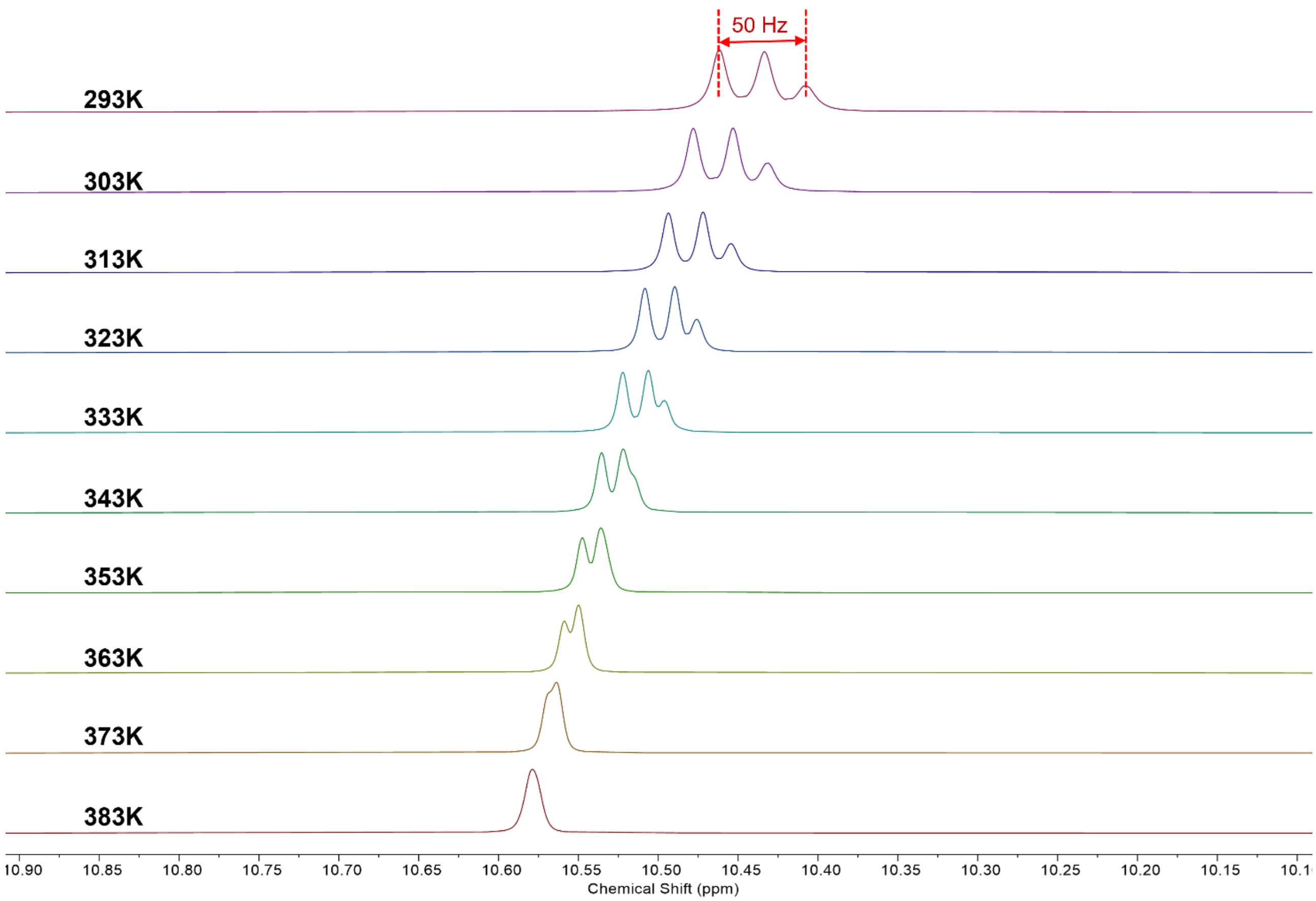

Fig. S2. Zoom in VT ^{1}H NMR spectrum of 1 for inner protons. The signal coalescing at high temperatures was due to the fast bowl-to-bowl inversion.

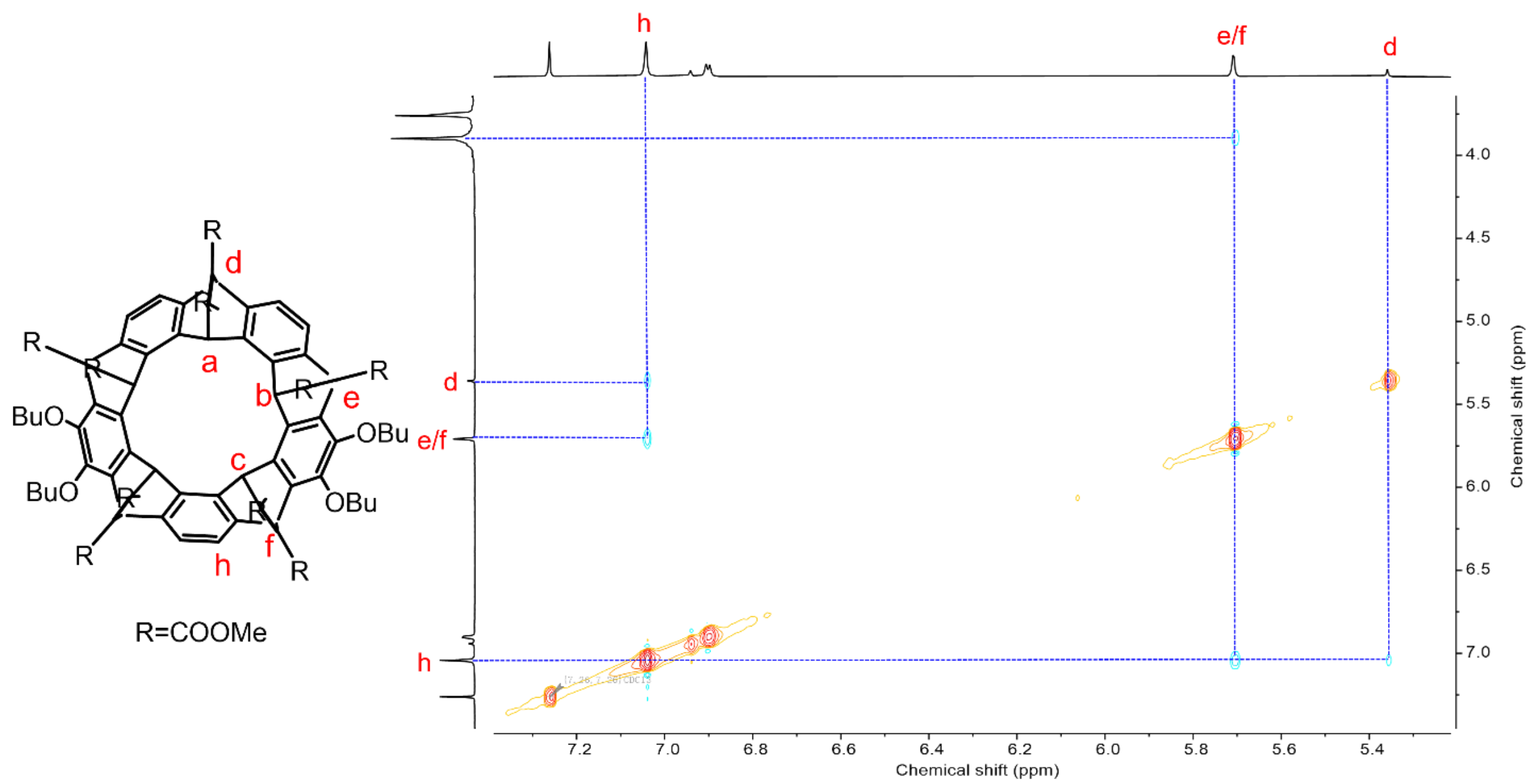

Fig. S3. 2D ROESY NMR spectrum of 9. Partial spectrum of **9** in CD_2Cl_2 (400 MHz) at 298 K showing aromatic/aromatic correlations.

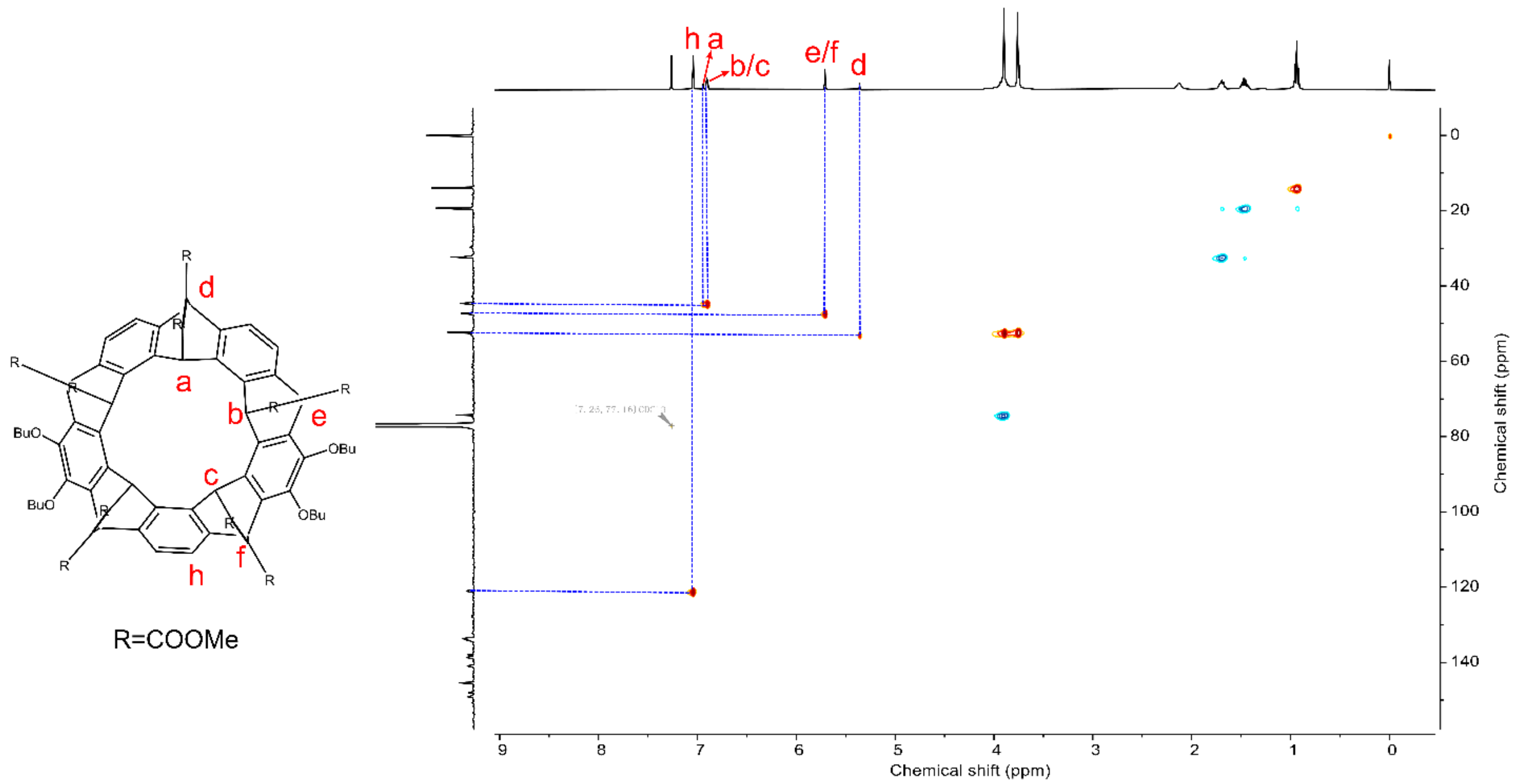

Fig. S4. 2D HSQC NMR spectrum of 9. Partial spectrum of **9** in CD_2Cl_2 (400 MHz) at 298 K showing C-H correlations.

VT ^{1}H NMR spectrum of **1** was recorded in $CDCl_2CDCl_2$. The exchange rate constant k_c was estimated at the coalescence temperature (T_C = 383 K) using equation(1), the obtained k_c value were then fitted with equation(2). In Equation 2, k_b is the Boltzmann constant and h is Planck's constant.

$$k_c = \frac{\pi \Delta v}{\sqrt{2}} \qquad \text{(Eq 1)}$$

$$\Delta G^{\ddagger} = RT_c \ln \frac{k_b T_c \sqrt{2}}{h \pi \Delta v} \qquad \text{(Eq 2)}$$

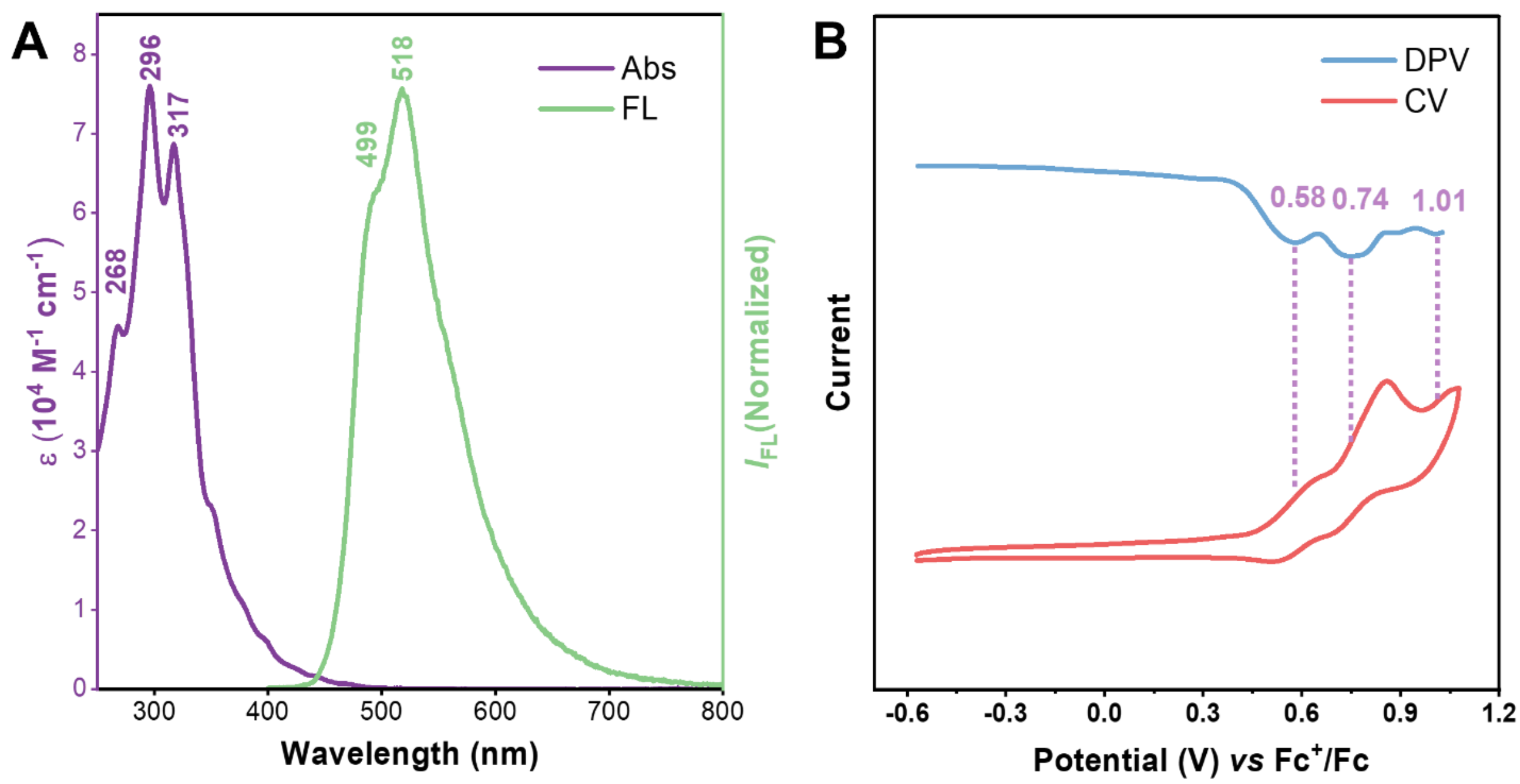

Fig. S5. Optical and electrochemical characterization of quintulene 1. (**A**), UV-vis absorption and normalized fluorescence spectra of **1** measured in DCM (1×10^{-5} M) at room temperature. (**B**), Cyclic voltammogram and differential pulse voltammogram of **1** measured in DCM with 0.1 M $TBAPF_6$ as the supporting electrolyte.

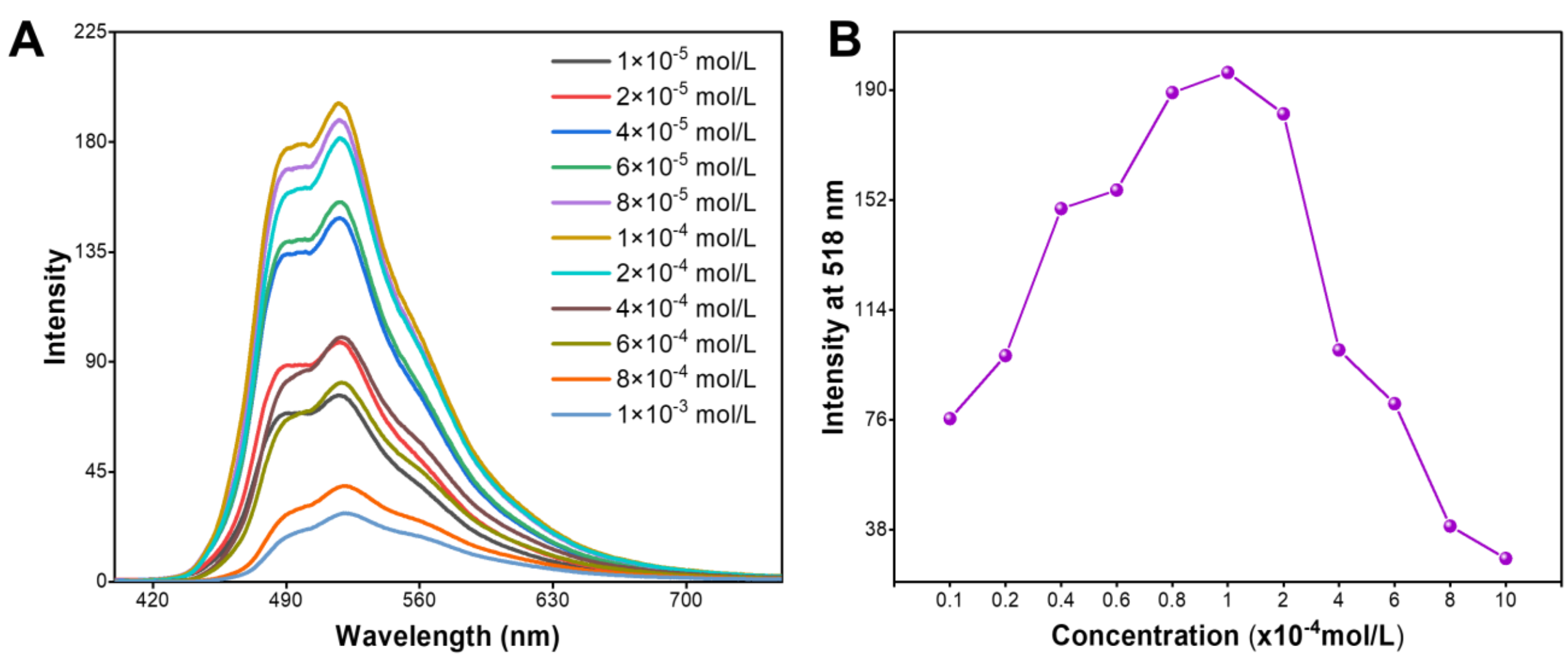

Fig. S6. Concentration-dependent fluorescence spectra. (**A**), Fluorescence spectra of **1** measured in DCM at different concentrations at room temperature. (**B**), The fluorescence intensity monitored at 518 nm for varying concentrations of **1**.

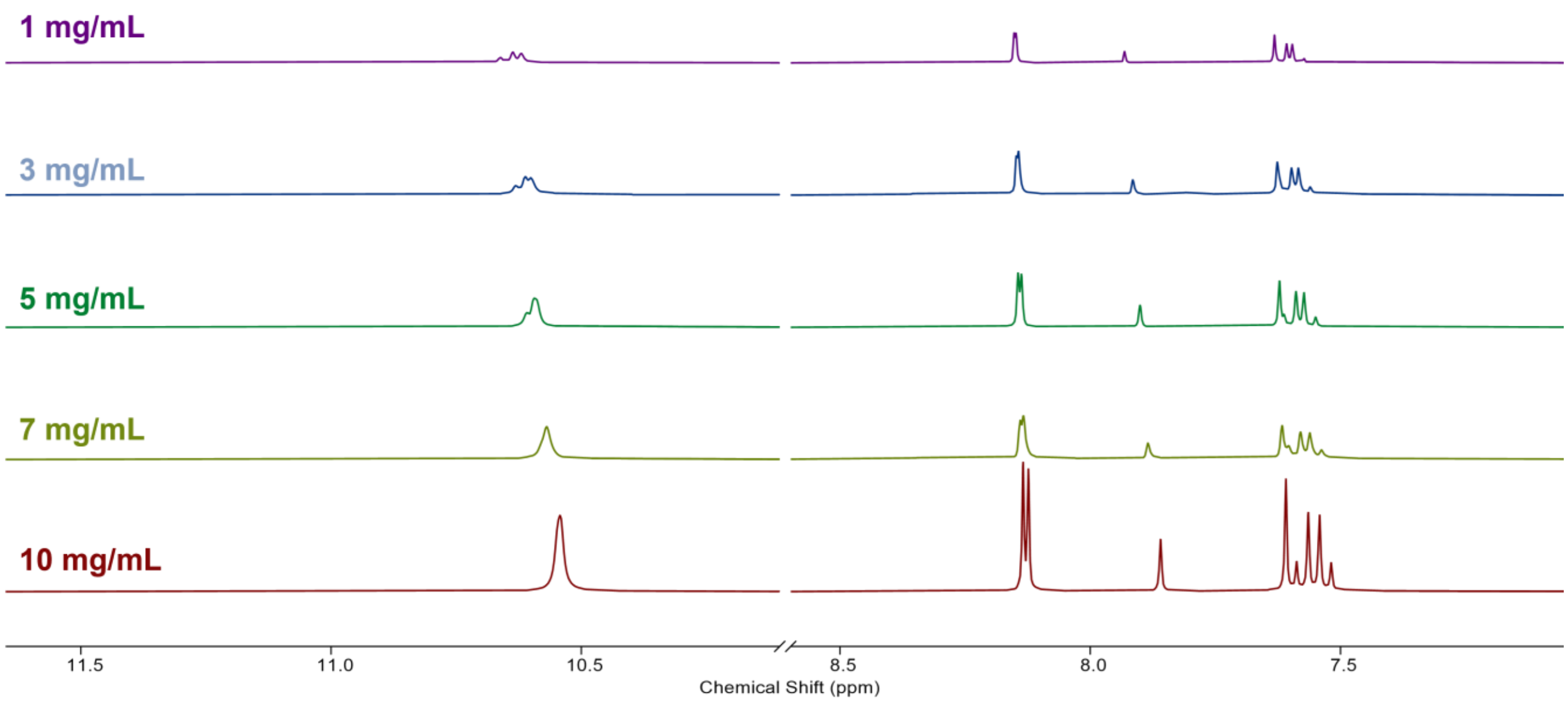

Fig. S7. Concentration-dependent ¹H NMR. The spectrum of the aromatic region was recorded in different concentrations ranging from 1 mg/mL to 10 mg/mL in $CDCl_2CDCl_2$ (400 MHz), the signals of inner protons gradually merged into a sharp singlet upon increasing the concentration to the 10 mg/mL.

4. DFT calculation

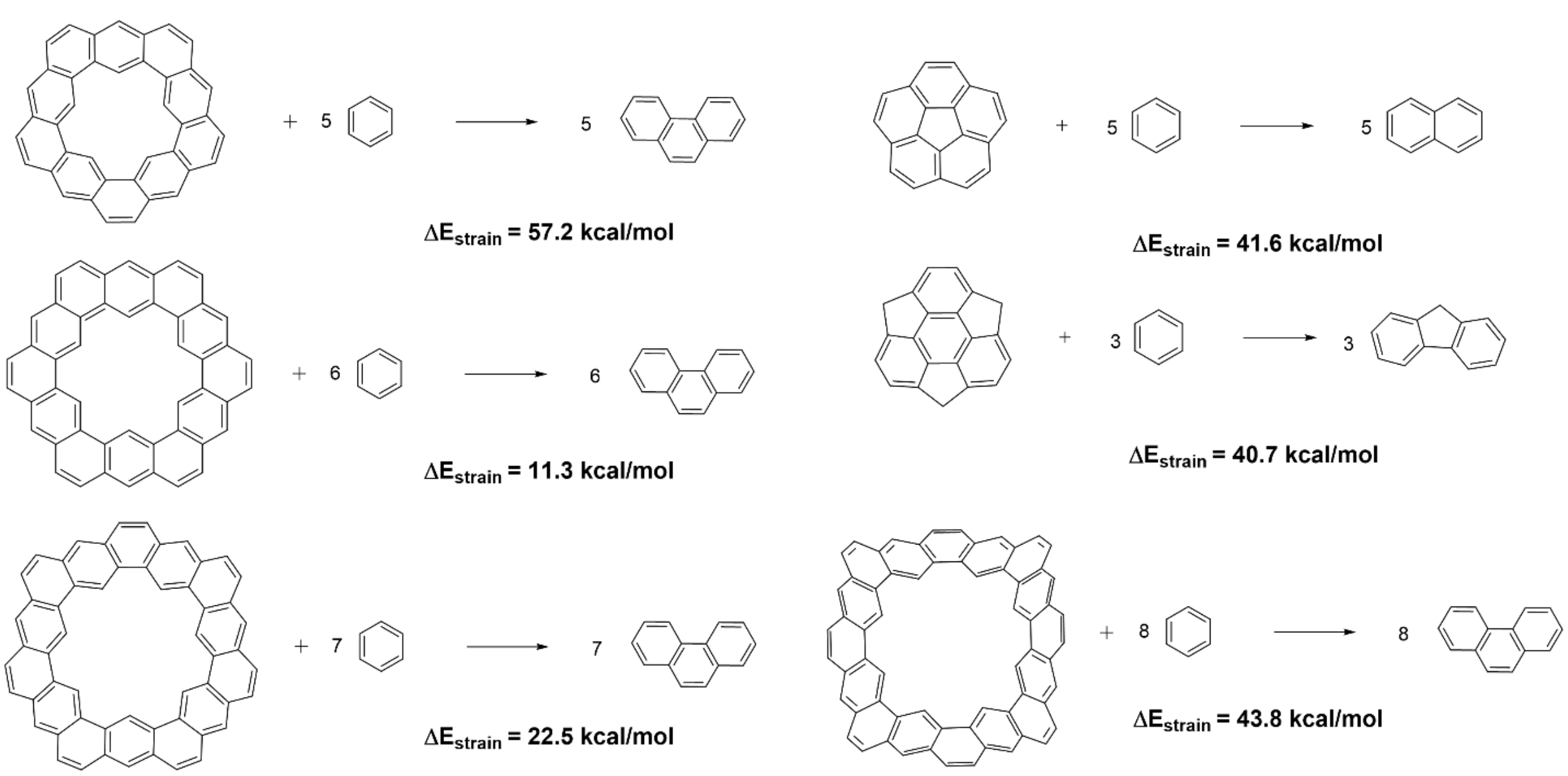

Fig. S8. Calculated strain energy. The hypothetical homodesmotic reactions applied in the calculation of strain energy for quintulene, kekulene, septulene, octulene, corannulene and sumanene. The substituents are replaced by hydrogen during the calculations (B3LYP/def2TZVP).

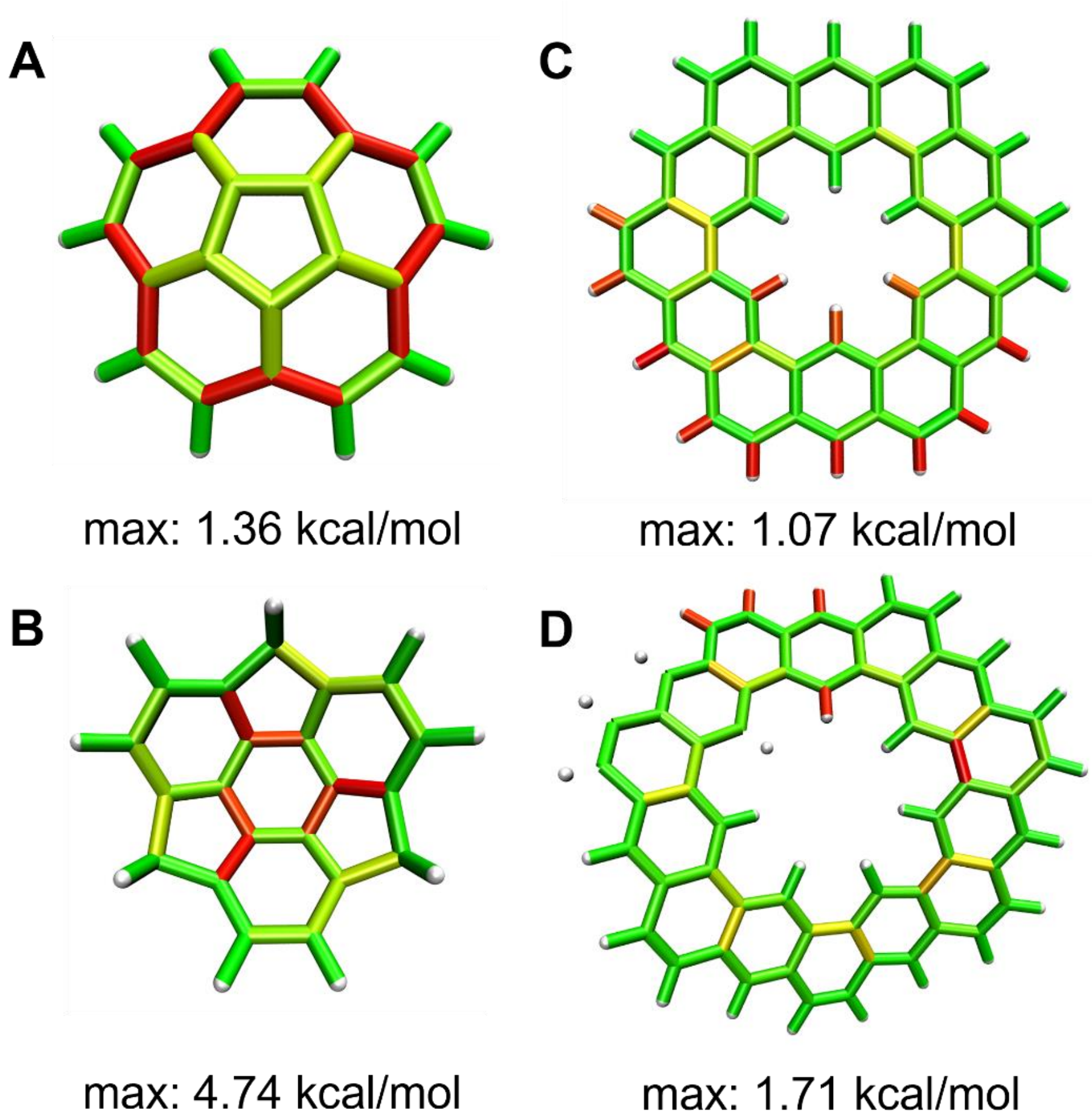

Fig. S9. Calculated StrainViz maps. (**A**), corannulene; (**B**), sumanene; (**C**), kekulene; (**D**), septulene. The substituents are omitted for clarity (B3LYP/6-31G(d,p).

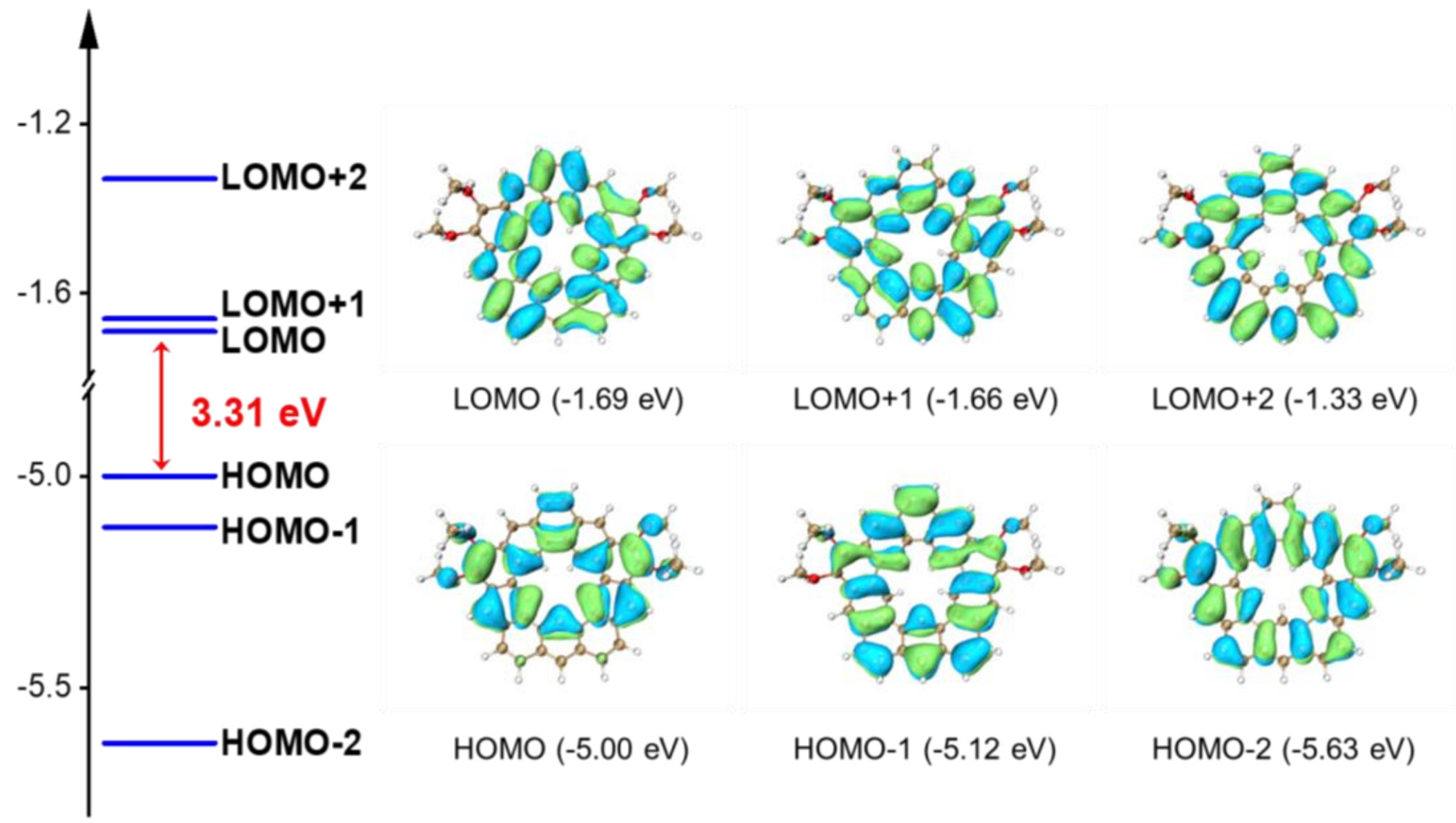

Fig. S10. Frontier molecular orbitals. Frontier molecular orbital profiles and energy diagram of **1** obtained by B3LYP/6-31G(d,p) level calculation. The substituents are replaced by methoxy groups during the calculations.

Table. S2. Selected TD-DFT (B3LYP/6-31G(d,p)) calculated energies, oscillator strength and compositions of major electronic transitions of 1.

Wavelength (nm)	Osc.Strength (f)	Major contributions
403.15	0.0246	H-1->L+1 (68%), HOMO->LUMO (19%), H-1->LUMO (8%), HOMO->L+1 (3%)
392.49	0.0225	H-1->L+3 (27%), HOMO->L+2 (57%), H-3->L+1 (2%), H-2->LUMO (4%), H-1->LUMO (2%)
392.07	0.0148	H-1->L+2 (32%), HOMO->L+3 (54%), H-3->LUMO (3%), H-2->L+1 (3%), HOMO->LUMO (2%)
364.26	0.0452	H-2->LUMO (20%), H-2->L+1 (33%), H-1->L+4 (16%), HOMO->L+3 (17%), H-1->L+2 (4%), H-1->L+3 (2%)
361.84	0.0218	H-3->L+1 (17%), H-2->LUMO (43%), H-2->L+1 (16%), H-3->LUMO (5%), HOMO->L+2 (9%), HOMO->L+4 (4%)
357.07	0.0179	H-3->LUMO (32%), H-1->L+4 (46%), H-4->L+1 (2%), H-3->L+1 (4%), H-2->L+1 (4%), HOMO->L+3 (3%)
355.59	0.4046	H-1->L+2 (18%), H-1->L+3 (32%), HOMO->L+2 (16%), H-3->LUMO (9%), H-3->L+1 (5%), H-2->LUMO (7%), HOMO->L+3 (6%), HOMO->L+4 (3%)
354.56	0.3536	H-1->L+2 (34%), H-1->L+3 (15%), H-1->L+4 (13%), HOMO->L+3 (10%), H-5->LUMO (3%), H-3->LUMO (6%), H-3->L+1 (6%), H-2->L+1 (5%), HOMO->L+2 (6%)
342.25	0.1253	H-4->LUMO (31%), H-3->L+1 (33%), H-2->LUMO (12%), H-4->L+1 (2%), H-3->LUMO (3%), H-3->L+2 (2%), H-1->L+3 (9%), HOMO->L+2 (2%)
331.30	0.3709	H-3->LUMO (27%), H-2->L+1 (25%), H-2->L+2 (19%), H-3->L+1 (4%), H-3->L+3 (9%), H-2->LUMO (2%), H-1->L+4 (6%)
328.87	0.1819	H-4->LUMO (10%), H-4->L+1 (63%), H-5->LUMO (4%), H-2->L+1 (2%), H-2->L+2 (6%), H-1->L+4 (8%)
327.99	0.0132	H-2->L+3 (82%), H-4->L+3 (2%), H-3->L+2 (6%), H-2->L+2 (2%)

323.30	0.5669	H-4->LUMO (47%), H-3->L+1 (15%), H-3->L+2 (10%), H-5->L+1 (2%), H-4->L+1 (4%), H-2->LUMO (3%), H-2->L+2 (4%), H-2->L+3 (3%), H-1->L+3 (2%), HOMO->L+4 (4%)
322.80	0.1212	H-3->L+2 (74%), H-3->L+1 (6%), H-2->LUMO (2%), H-2->L+3 (3%), HOMO->L+4 (5%)
322.36	0.1874	H-4->L+1 (21%), H-2->L+2 (43%), H-3->LUMO (8%), H-3->L+3 (6%), H-2->L+1 (4%), H-1->L+4 (6%)
293.44	0.7639	H-5->LUMO (38%), H-5->L+1 (30%), H-4->L+2 (4%), H-4->L+3 (7%)
292.37	0.7695	H-5->LUMO (32%), H-5->L+1 (41%), H-4->L+2 (3%), H-4->L+3 (3%), H-3->L+3 (2%), HOMO->L+5 (3%), HOMO->L+6 (2%)
271.16	0.2001	H-5->L+2 (19%), HOMO->L+5 (58%), H-6->L+1 (2%), H-5->LUMO (3%), HOMO->L+6 (7%), HOMO->L+7 (2%)
269.99	0.1516	H-5->L+2 (29%), HOMO->L+5 (26%), HOMO->L+6 (13%), H-6->LUMO (2%), H-5->L+3 (2%), H-5->L+4 (6%), H-4->L+3 (2%), HOMO->L+7 (8%)
266.12	0.2457	H-1->L+5 (52%), HOMO->L+7 (13%), H-7->L+1 (4%), H-6->LUMO (4%), H-5->L+1 (2%), H-5->L+2 (3%), H-1->L+6 (7%), H-1->L+7 (2%)
265.41	0.1755	H-1->L+7 (29%), HOMO->L+6 (40%), H-6->LUMO (2%), H-5->L+2 (9%), H-5->L+3 (3%), H-1->L+5 (5%)
264.61	0.047	H-1->L+5 (25%), H-1->L+6 (29%), HOMO->L+7 (27%), H-5->L+3 (6%)
259.41	0.0379	H-7->LUMO (67%), H-6->LUMO (12%), H-1->L+6 (5%), HOMO->L+7 (3%)
258.27	0.1118	H-6->LUMO (30%), H-6->L+1 (25%), H-1->L+7 (10%), H-7->LUMO (9%), H-7->L+1 (9%)
257.57	0.1514	H-7->L+1 (44%), H-6->LUMO (15%), H-8->LUMO (3%), H-8->L+1 (3%), H-6->L+1 (3%), H-5->L+2 (4%), H-1->L+5 (7%), H-1->L+7 (3%), HOMO->L+6 (3%)
235.13	0.1567	H-2->L+6 (42%), H-2->L+7 (13%), H-7->L+2 (8%), H-7->L+3 (4%), H-6->L+3 (4%), H-3->L+6 (2%), HOMO->L+9 (4%)
232.67	0.3814	H-3->L+6 (49%), H-8->L+2 (5%), H-6->L+3 (2%), H-4->L+5 (2%), H-3->L+7 (9%), H-2->L+6 (5%), H-2->L+7 (7%), H-1->L+9 (4%)
232.28	0.185	H-3->L+7 (54%), H-11->L+1 (4%), H-7->L+3 (4%), H-6->L+2 (9%), H-3->L+6 (6%), H-1->L+9 (4%), HOMO->L+8 (3%)
219.58	0.1253	H-11->L+2 (11%), H-10->L+2 (10%), H-10->L+3 (40%), H-13->L+1 (3%), H-10->LUMO (3%), H-9->L+4 (6%), H-4->L+7 (4%), H-2->L+9 (2%)
216.06	0.1066	H-3->L+8 (18%), H-2->L+9 (40%), H-11->L+2 (5%), H-11->L+4 (7%), H-10->LUMO (3%), H-8->L+4 (2%)
215.58	0.1403	H-12->L+1 (10%), H-11->L+3 (12%), H-2->L+8 (28%), H-13->LUMO (6%), H-11->LUMO (3%), H-10->L+2 (3%), H-10->L+4 (5%), H-4->L+7 (3%), H-3->L+9 (9%)
212.98	0.1523	H-11->L+2 (32%), H-3->L+8 (12%), H-13->L+1 (8%), H-12->L+3 (7%), H-11->L+3 (6%), H-10->L+3 (9%), H-5->L+5 (2%), H-2->L+9 (7%)
208.43	0.1268	H-12->L+3 (13%), H-11->L+2 (12%), H-11->L+4 (22%), H-10->L+3 (2%), H-5->L+6 (7%), H-5->L+7 (3%), H-4->L+9 (5%), H-3->L+8 (2%), H-3->L+9 (2%), H-2->L+9 (2%), HOMO->L+10 (2%)
207.67	0.1139	H-12->L+2 (10%), H-10->L+4 (13%), H-4->L+8 (14%), H-17->LUMO (2%), H-14->LUMO (4%), H-13->L+3 (2%), H-11->L+3 (8%), H-10->L+2 (4%), H-5->L+6 (4%), H-5->L+7 (7%), HOMO->L+11 (7%)
207.18	0.1365	H-11->L+4 (34%), H-4->L+9 (15%), HOMO->L+10 (11%), H-17->LUMO (5%), H-17->L+1 (3%), H-16->L+1 (3%), H-1->L+11 (9%)

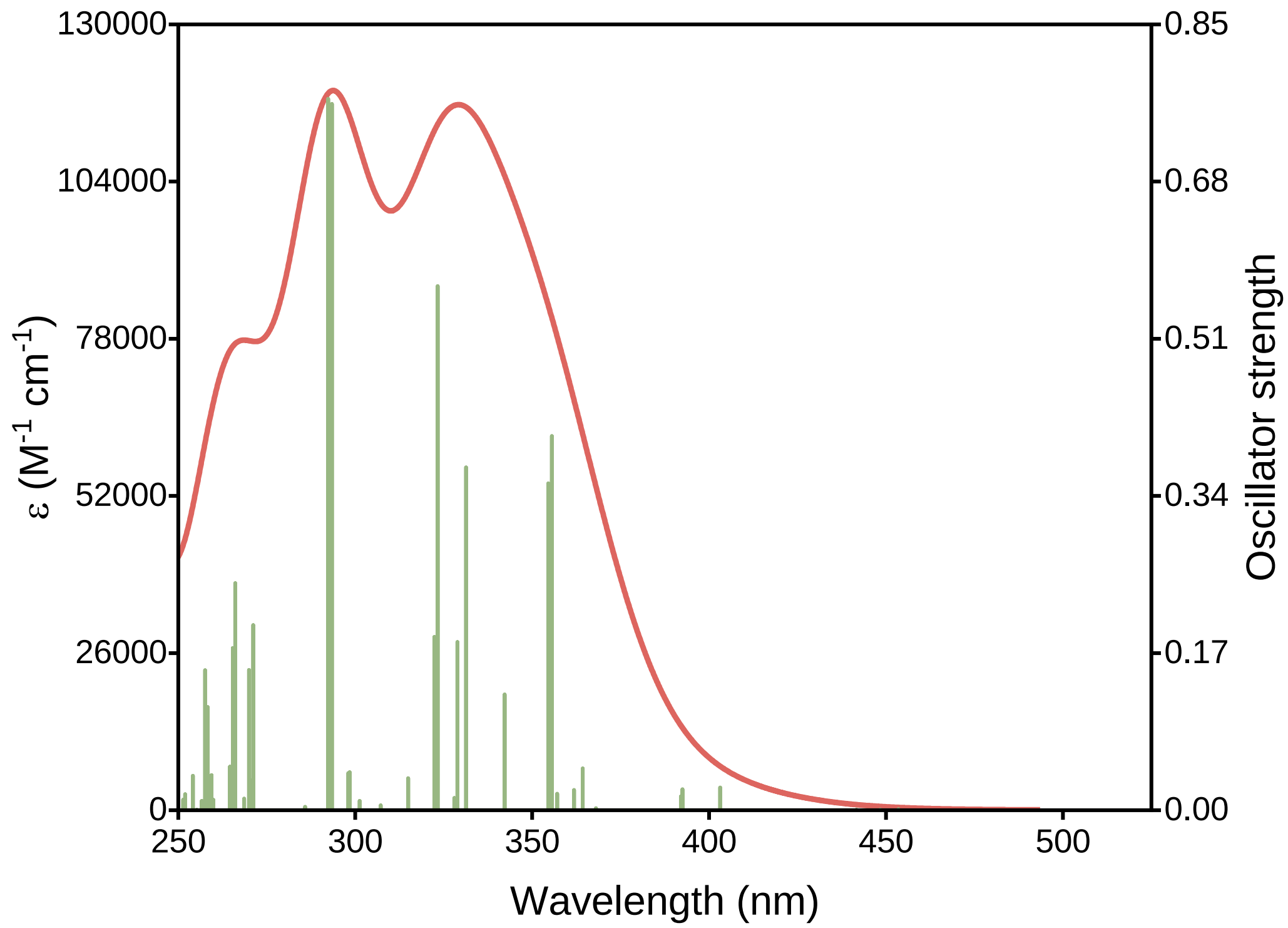

Fig. S11. Calculated electronic absorption spectrum. The electronic absorption spectrum of **1** was obtained at the level of B3LYP/6-31G(d,p). Full width at half maximum (FWHM) is set to 0.4 eV.

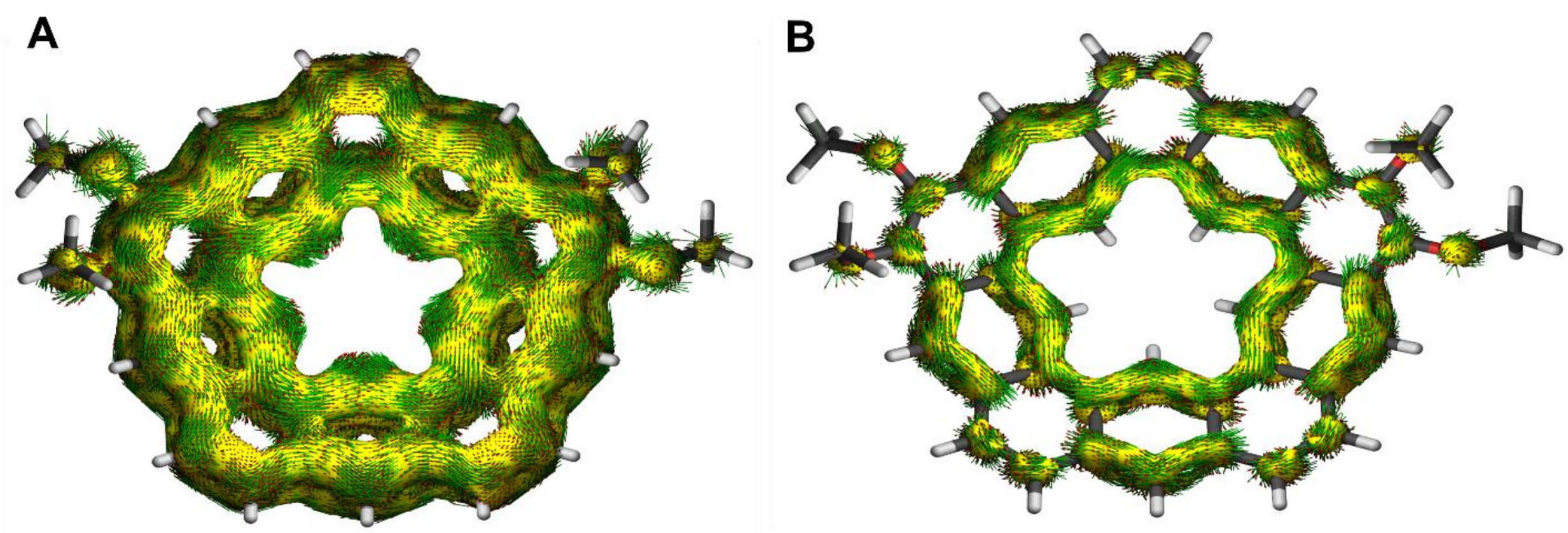

Fig. S12. Calculated ACID plots. The AICD plots of **1** with isovalue of 0.02 (**A**) and 0.06 (**B**) were calculated at the level of B3LYP/6-31G(d,p). The magnetic field is pointing out through the paper. The substituents are replaced by methoxy groups during the calculations.

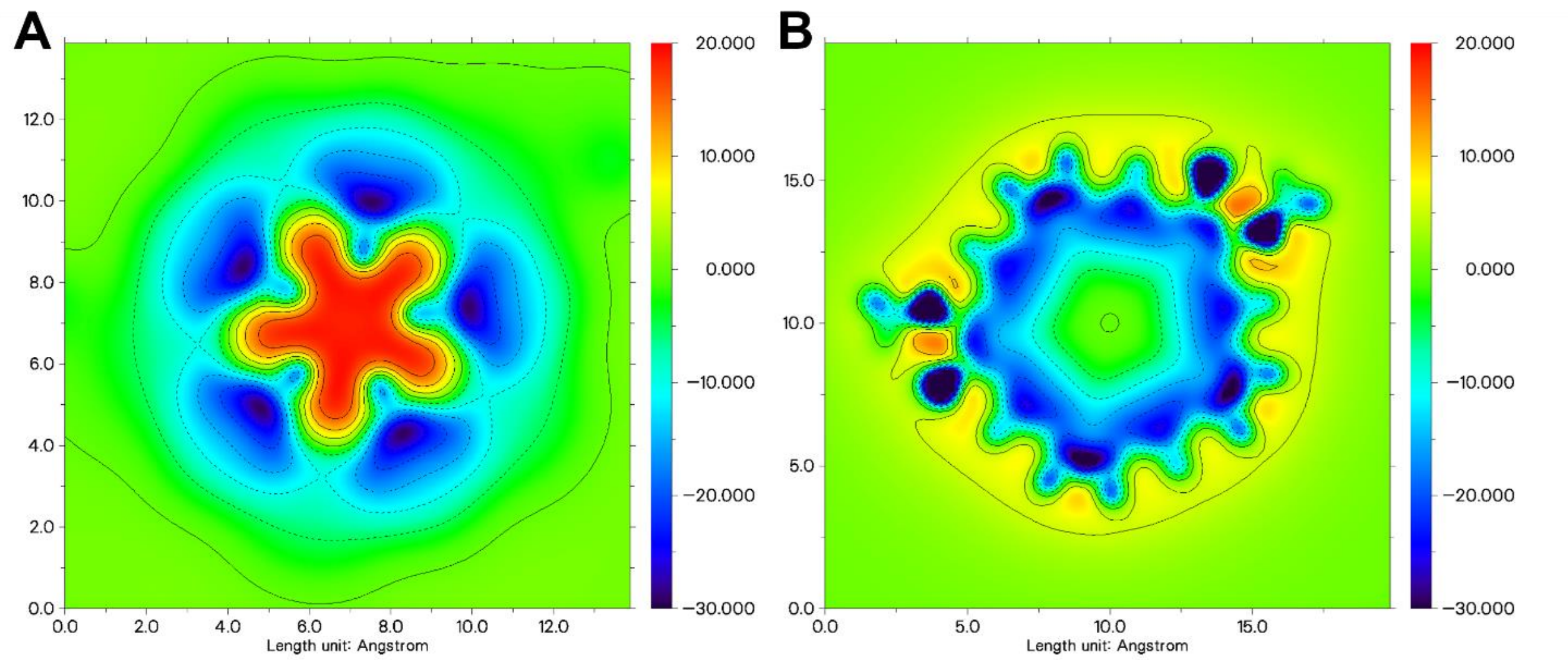

Fig. S13. Calculated 2D NICS$_{zz}$. (**A**), Calculated 2D NICS(1)$_{zz}$ for the outer ring of **1.** (**B**), 2D NICS(-1)$_{zz}$ for the inner ring of **1**. The substituents were replaced by methoxy groups during the calculations.

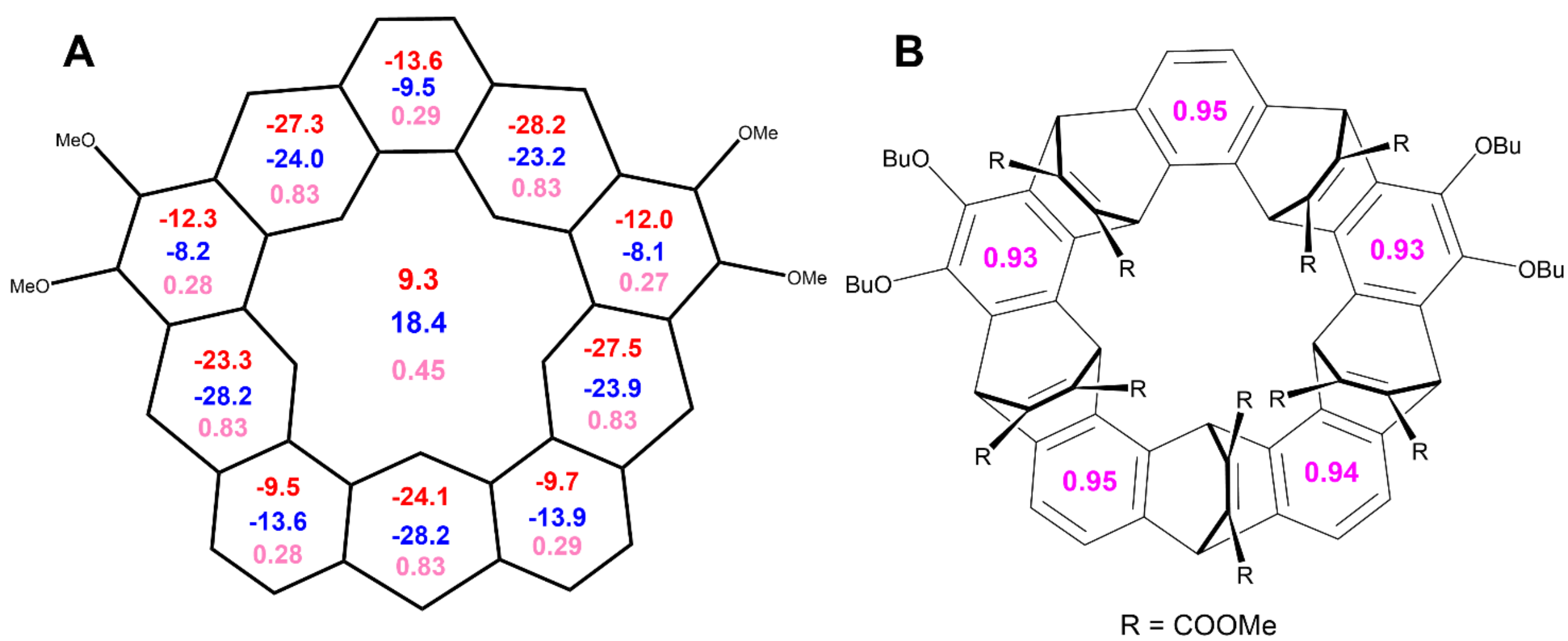

Fig. S14. Calculated NICS$_{zz}$ and HOMA values. (**A**), Calculated NICS(+1)$_{zz}$ values, NICS(-1)$_{zz}$ values and HOMA values (the numbers in red, blue and pink color, respectively) of **1**. (**B**), Calculated HOMA values of **9**. The substituents of **1** were replaced by methoxy groups during the calculations and the magnetic field is oriented perpendicular to the XY plane, pointing out through the paper.

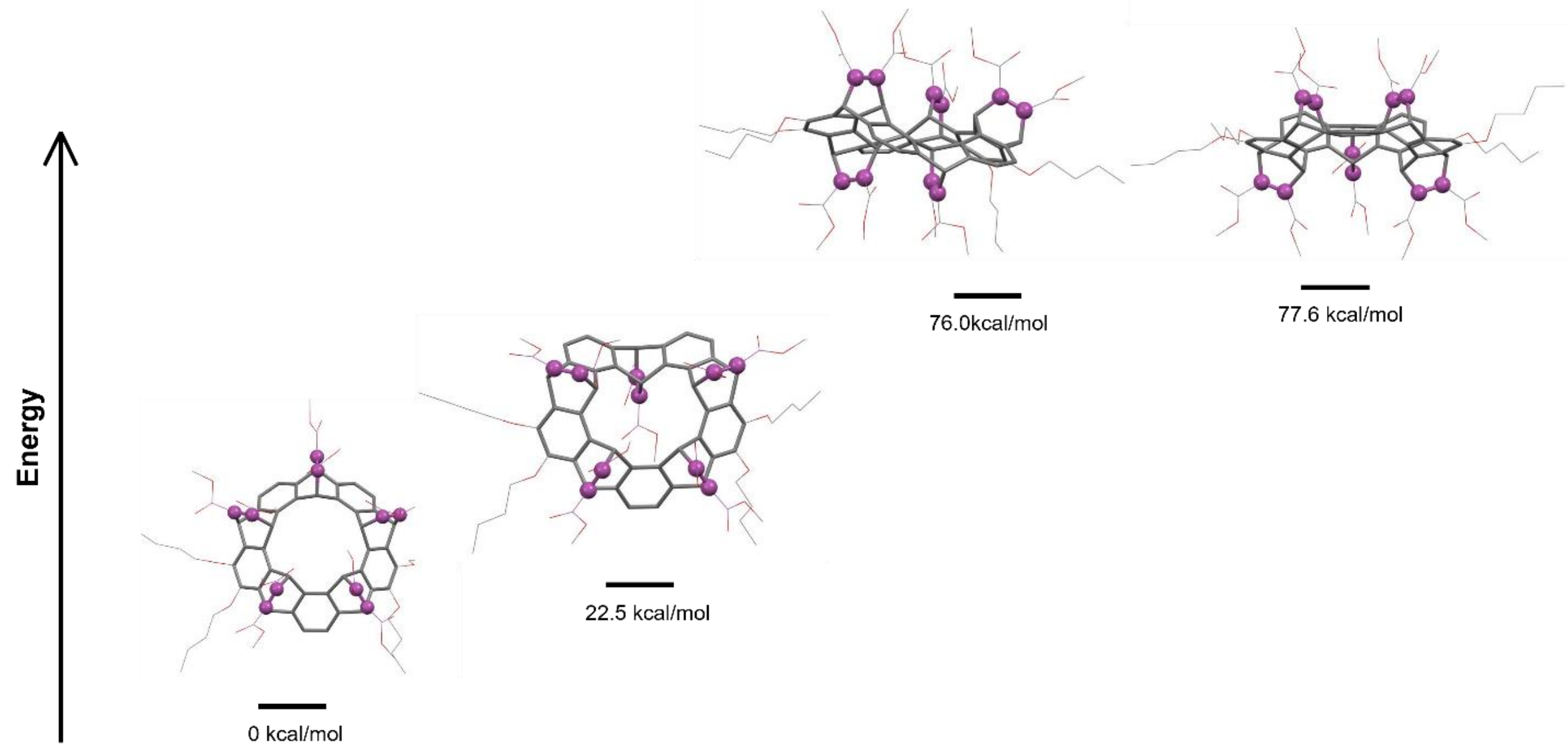

Fig. S15. Energy comparison of different geometries. Calculated electronic energy comparison of **9** with different possible geometries at the level of B3LYP/def2TZVP.

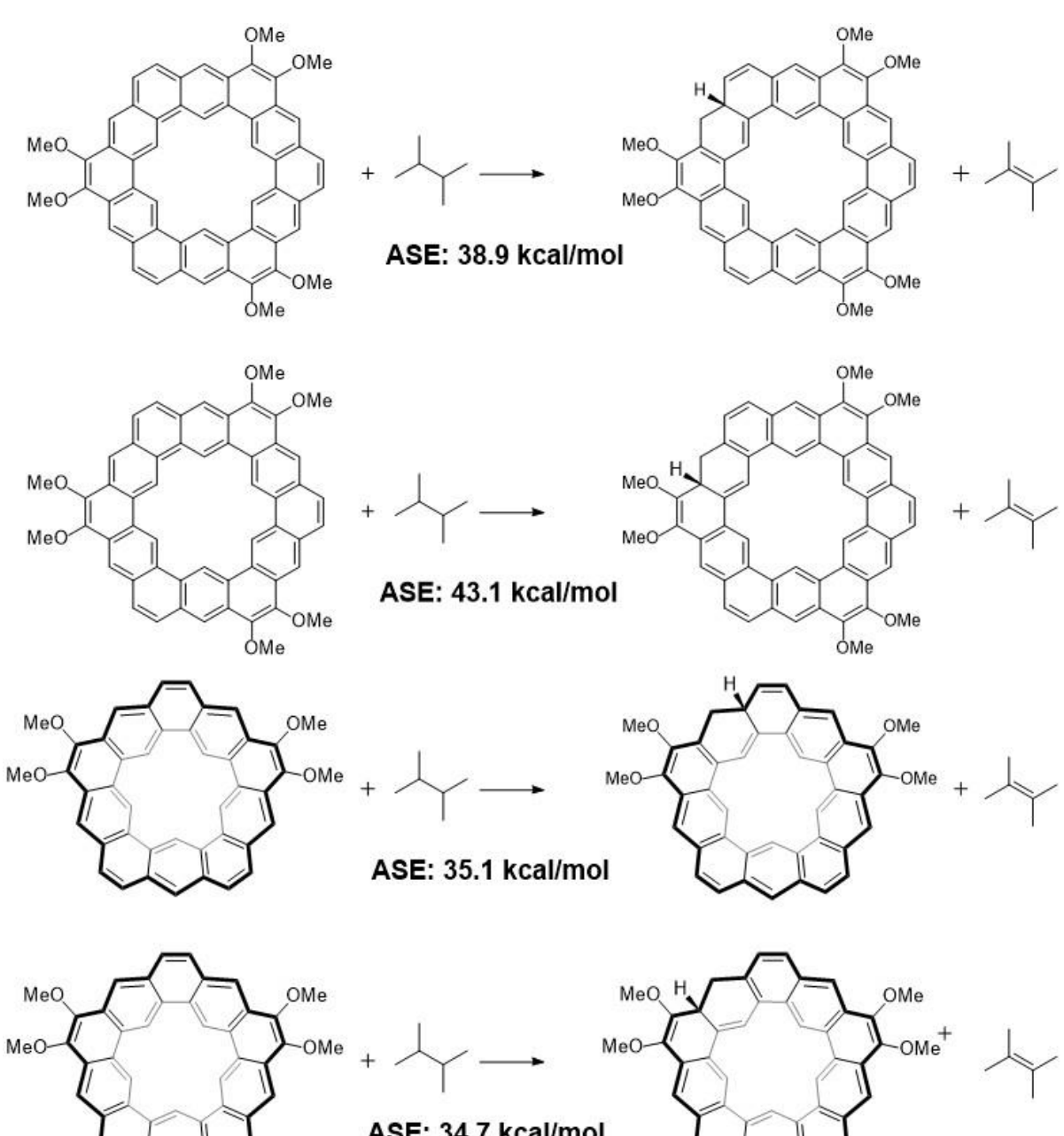

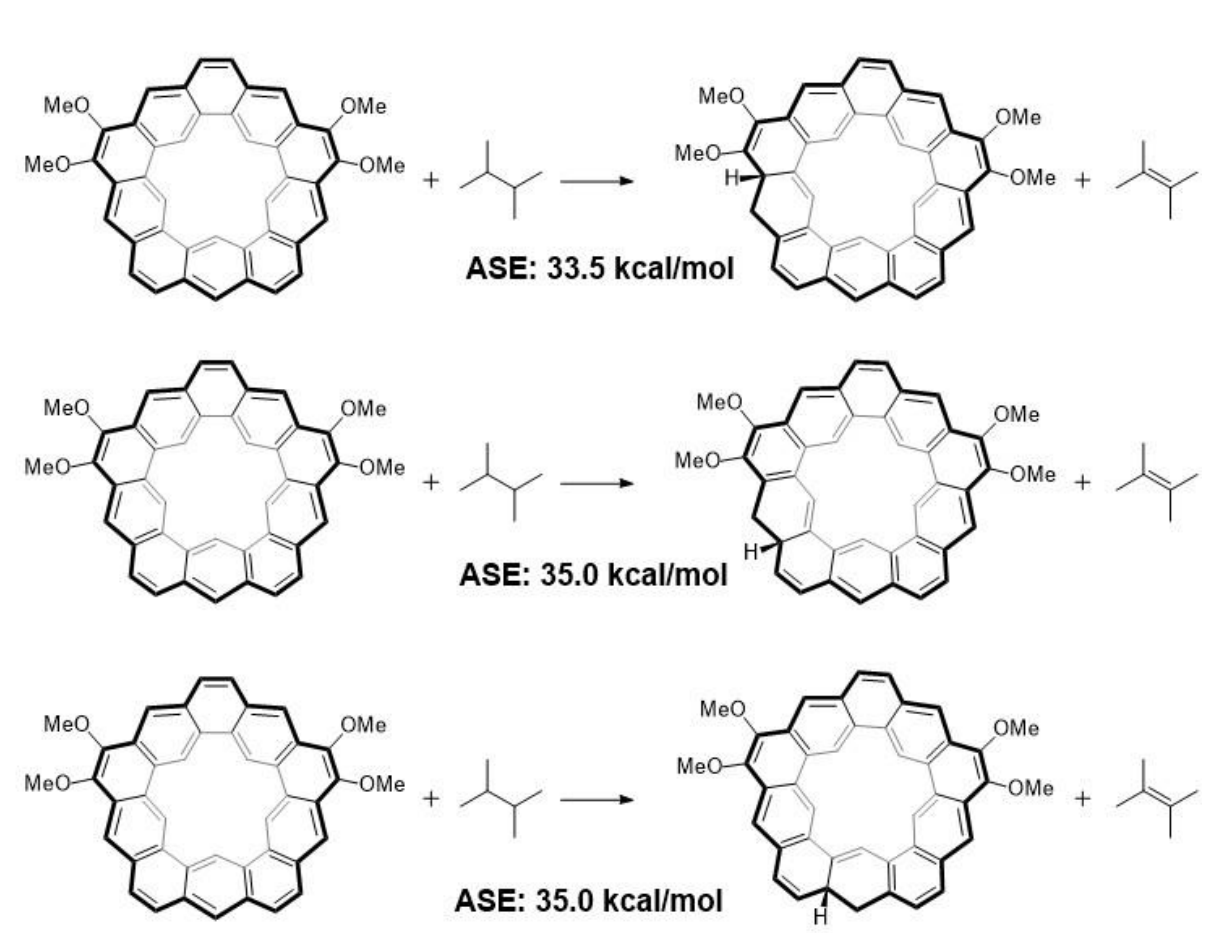

	average ASE
kekulene	41.0 kcal/mol
quintulene	34.7 kcal/mol

Fig. S16. Comparison of ASE. Calculated aromatic stabilization energy (ASE) of the localized benzenoid rings in both kekulene and quintulene **1** by the hydrogenation of outer double bond at the level of B3LYP/6-31G(d,p).

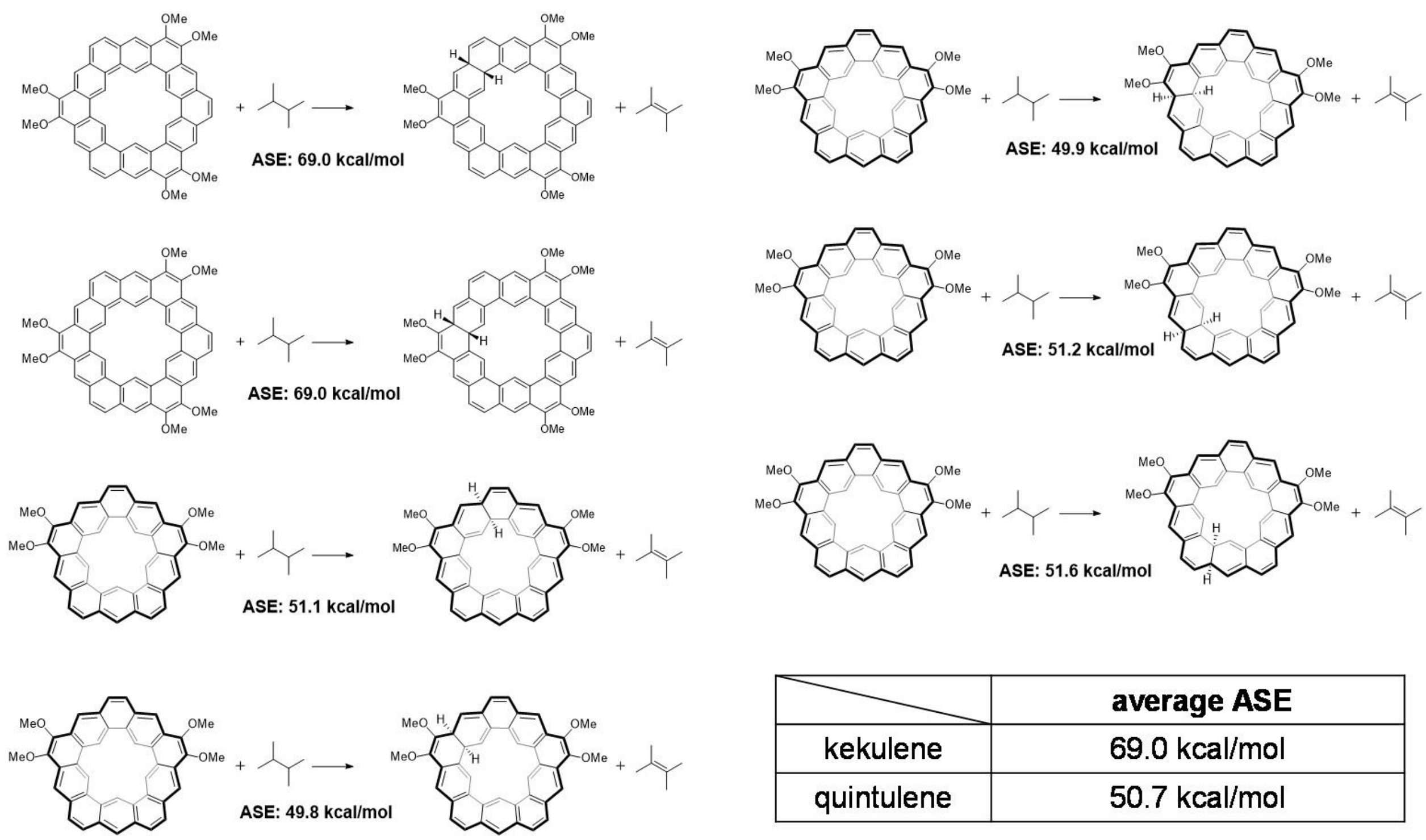

	average ASE
kekulene	69.0 kcal/mol
quintulene	50.7 kcal/mol

Fig. S17. Comparison of ASE. Calculated aromatic stabilization energy (ASE) of the localized benzenoid rings in both kekulene and quintulene **1** by the hydrogenation of interlinkage double bond at the level of B3LYP/6-31G(d,p).

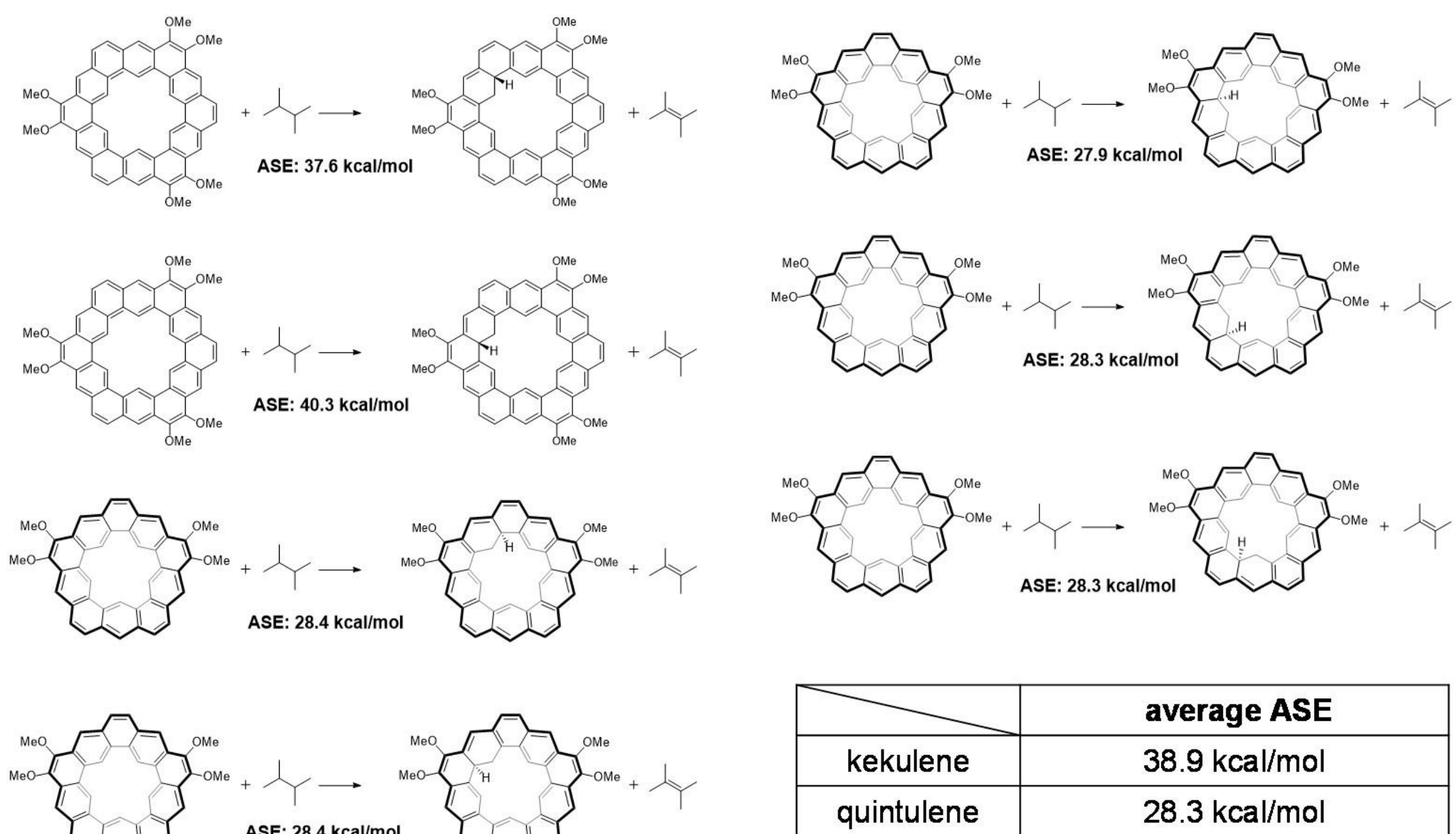

	average ASE
kekulene	38.9 kcal/mol
quintulene	28.3 kcal/mol

Fig. S18. Comparison of ASE. Calculated aromatic stabilization energy (ASE) of the localized benzenoid rings in both kekulene and quintulene **1** by the hydrogenation of inner double bond at the level of B3LYP/6-31G(d,p).

5. X-ray crystallographic data

Table. S3. Crystallographic data of 7.

Empirical formula	$C_{112}H_{112}Br_{12}O_8$
Formula weight	2544.93
CCDC No	2496960
Temperature/K	100.00
Crystal system	triclinic
Space group	P-1
a/Å	12.8818(9)
b/Å	16.3007(12)
c/Å	27.9818(19)
α/°	74.260(3)
β/°	89.853(3)
γ/°	67.856(3)
Volume/$Å^3$	5205.4(6)
Z	2
$r_{calc}g/cm^3$	1.624
μ/mm^{-1}	3.923
F(000)	2536.0
Crystal size/mm^3	0.15 × 0.12 × 0.12
Radiation	Ga Kα (λ = 1.34139)
2 theta range for data collection/°	5.26 to 104.192
Index ranges	$-15 \leq h \leq 15$, $-19 \leq k \leq 19$, $-32 \leq l \leq 32$
Reflections collected	117333
Independent reflections	17732 [R_{int}= 0.0515, R_{sigma} = 0.0300]
Data/restraints/parameters	17732/0/1197
Goodness-of-fit on F^2	1.056
Final R indexes [I>=2σ (I)]	R_1 = 0.0383, wR2 = 0.0949
Final R indexes [all data]	R_1 = 0.0447, wR2 = 0.0975

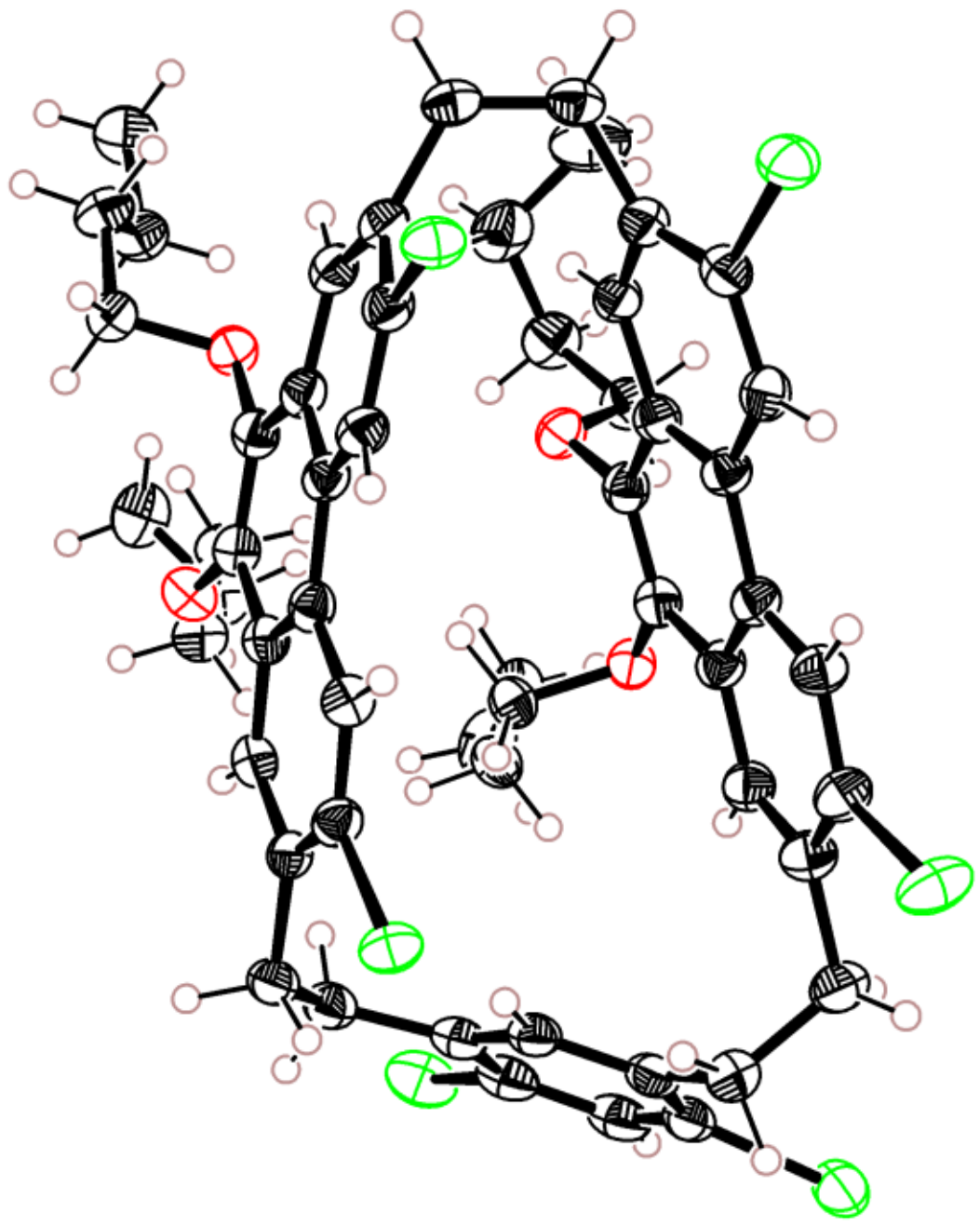

Fig. S19. X-ray structure. ORTEP drawings of the **7** showing thermal ellipsoids at the 50% probability level.

Table. S4. Crystallographic data of 5.

Empirical formula	$C_{57}H_{53}Br_6Cl_3O_4$
Formula weight	1387.80
CCDC No.	2496972
Temperature/K	200.0(2)
Crystal system	triclinic
Space group	P-1
a/Å	12.7874(4)
b/Å	15.6419(4)
c/Å	15.8714(5)
α/°	106.5730(10)
β/°	93.8370(10)
γ/°	110.7430(10)
Volume/Å^3	2795.08(14)
Z	2
ρ_{calc} g/cm^3	1.649
μ/mm^{-1}	4.497
F(000)	1376.0
Crystal size/mm^3	0.31 × 0.28 × 0.19
Radiation	Mo Kα (λ = 0.71073)
2 theta range for data collection/°	3.988 to 54.986
Index ranges	$-16 \leq h \leq 16$, $-20 \leq k \leq 20$, $-20 \leq l \leq 20$
Reflections collected	96707

Independent reflections	12816 [R_{int}= 0.0628, R_{sigma}= 0.0355]
Data/restraints/parameters	12816/0/636
Goodness-of-fit on F^2	1.022
Final R indexes [I>=2σ (I)]	R_1= 0.0370, wR_2= 0.0792
Final R indexes [all data]	R_1= 0.0613, wR_2= 0.0880

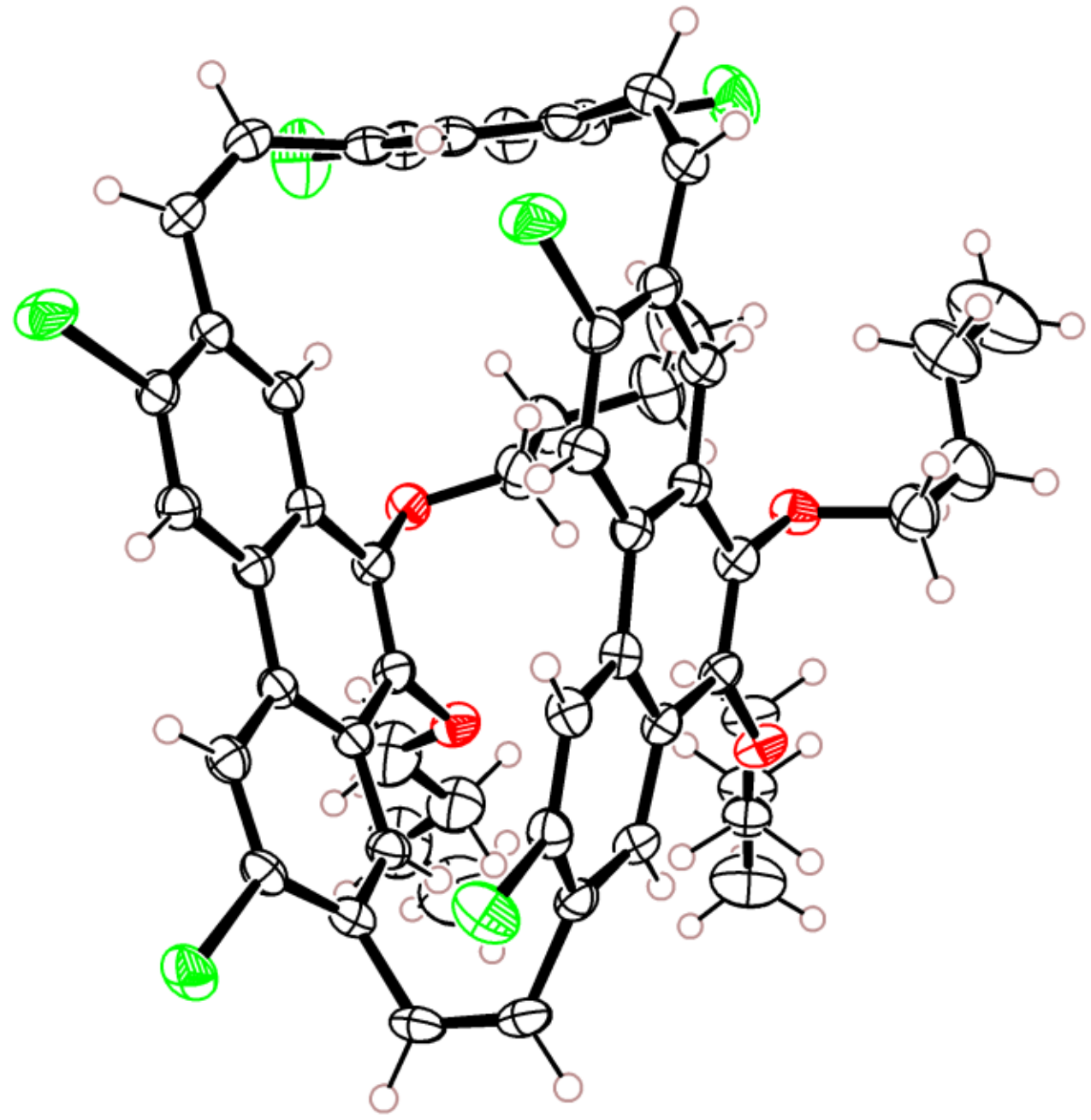

Fig. S20. X-ray structure. ORTEP drawings of the **5** showing thermal ellipsoids at the 50% probability level.

Table. S5. Crystallographic data of 1.

Empirical formula	$C_{57}H_{53}Cl_3O_4$
Formula weight	908.34
CCDC No.	2496973
Temperature/K	100.0(2)
Crystal system	monoclinic
Space group	$P2_1/n$
a/Å	21.136(2)
b/Å	7.6784(8)
c/Å	28.453(3)
α/°	90
β/°	102.958(6)
γ/°	90
Volume/Å^3	4500.0(8)
Z	4
ρ_{calc}g/cm^3	1.341
μ/mm^{-1}	1.502
F(000)	1912.0

Crystal size/mm^3	0.55 × 0.23 × 0.21
Radiation	Ga Kα (λ = 1.34138)
2 theta range for data collection/°	8.322 to 114.074
Index ranges	$-26 \leq h \leq 26$, $-9 \leq k \leq 9$, $-35 \leq l \leq 35$
Reflections collected	131772
Independent reflections	9227 [R_{int}= 0.0799, R_{sigma}= 0.0369]
Data/restraints/parameters	9227/340/679
Goodness-of-fit on F^2	1.081
Final R indexes [I>=2σ (I)]	R_1= 0.0756, wR_2= 0.2363
Final R indexes [all data]	R_1= 0.0850, wR_2= 0.2491

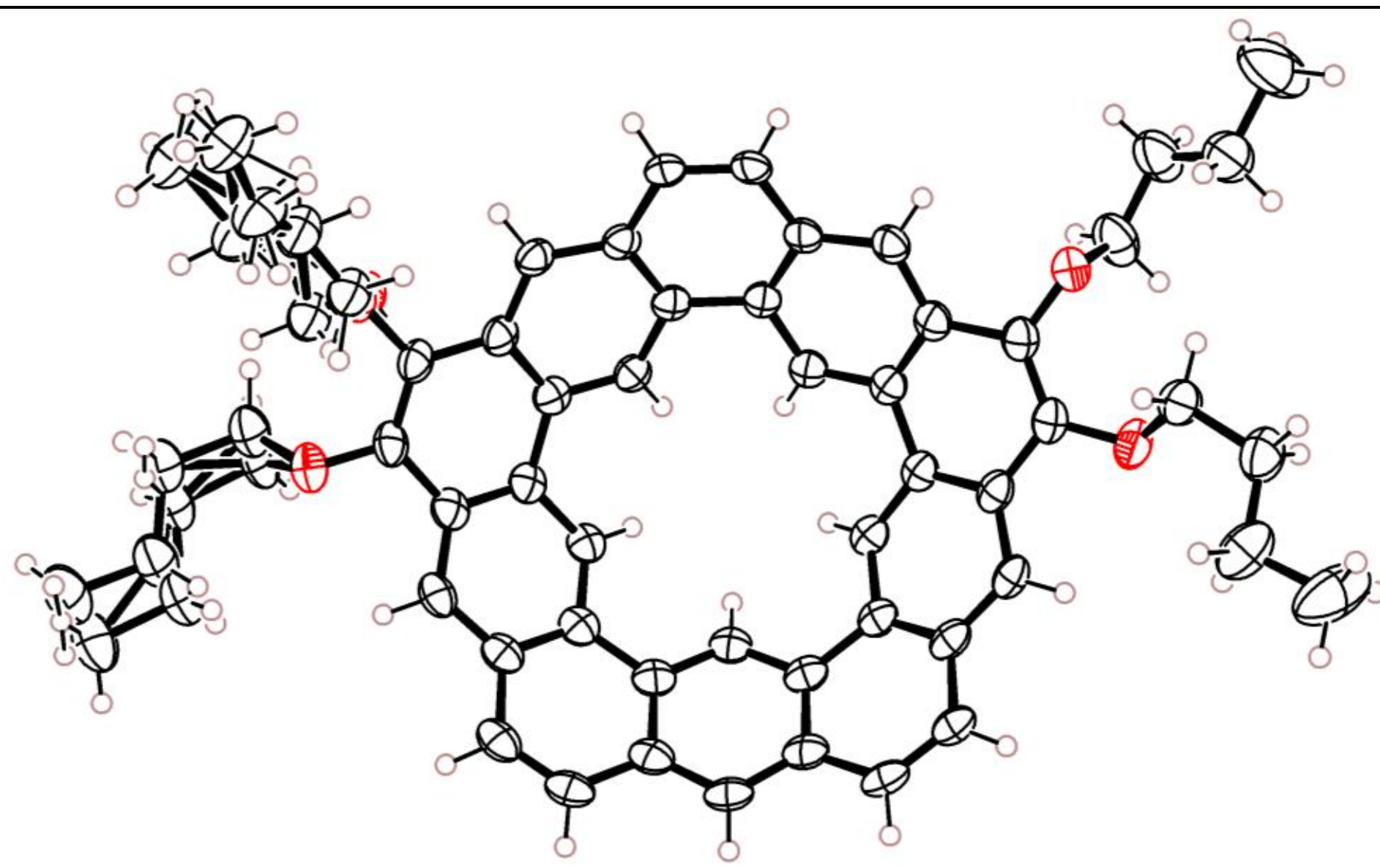

Fig. S21. X-ray structure. ORTEP drawings of the **1** showing thermal ellipsoids at the 50% probability level.

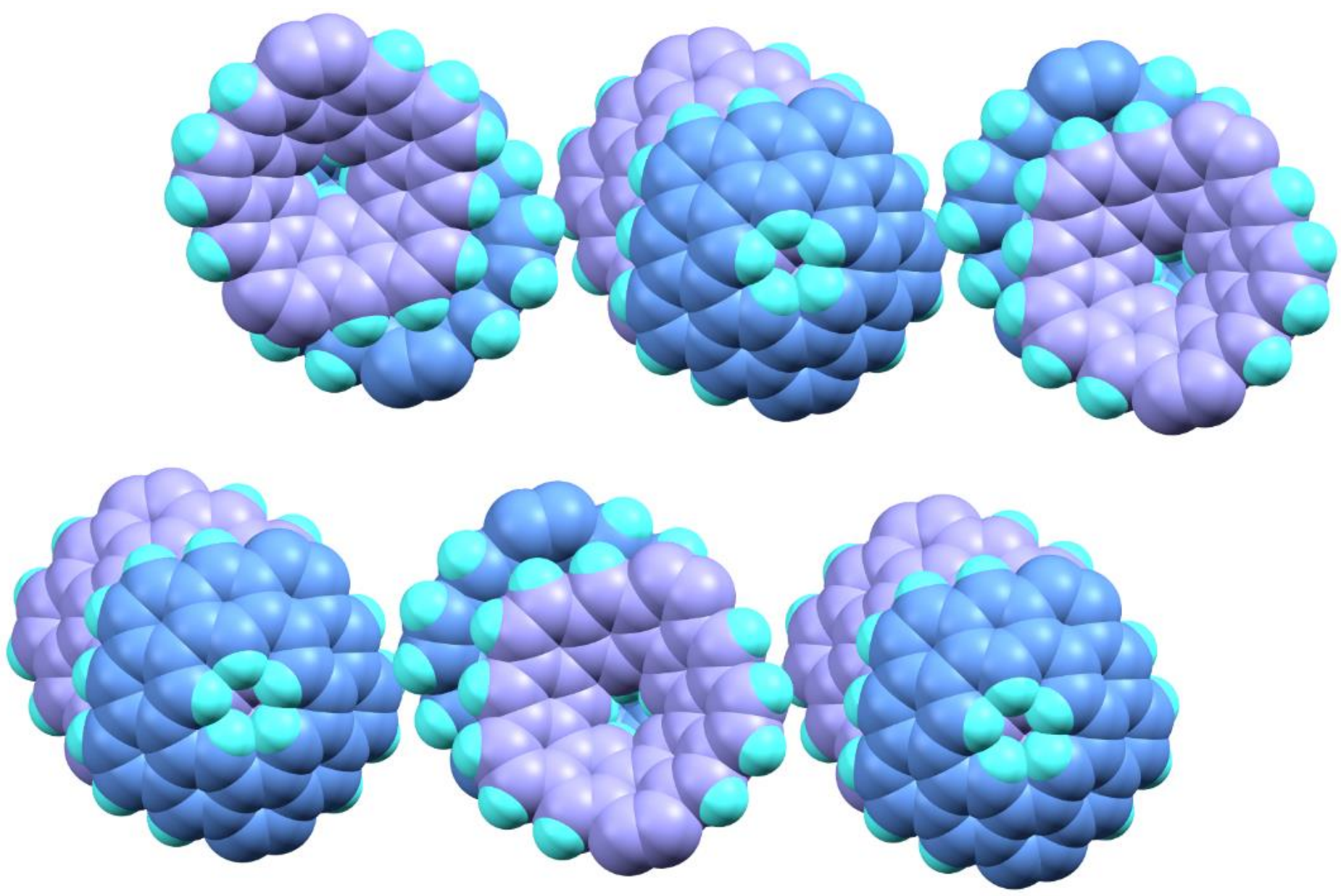

Fig. S22. Packing model. 3D packing structures of **1**, all substituents are omitted for clarity.

6. NMR and mass spectra

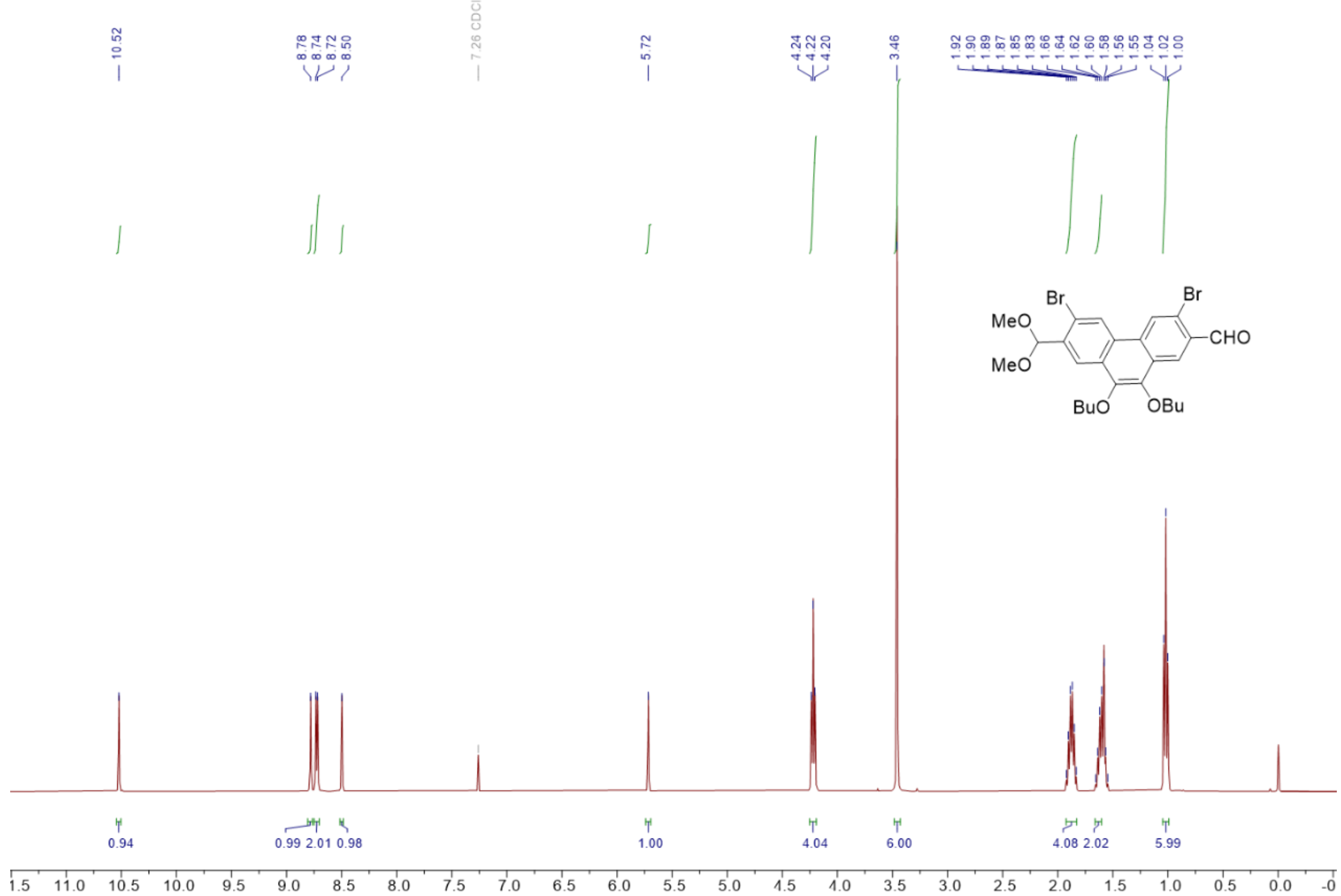

Fig. S23. ^{1}H NMR spectrum of 2 in $CDCl_3$ (400 MHz, 298 K).

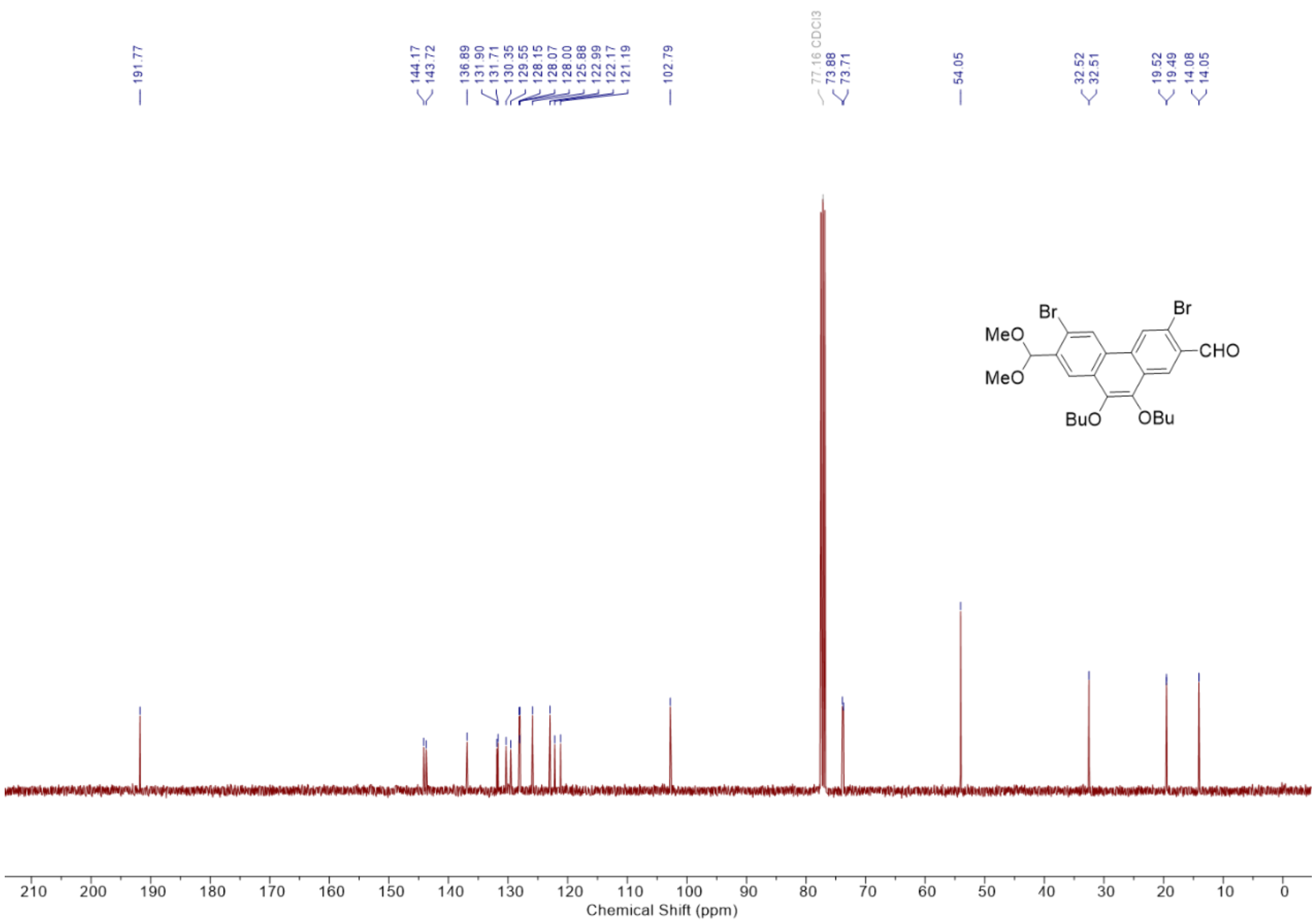

Fig. S24. ^{13}C NMR spectrum of 2 in $CDCl_3$ (100 MHz, 298 K).

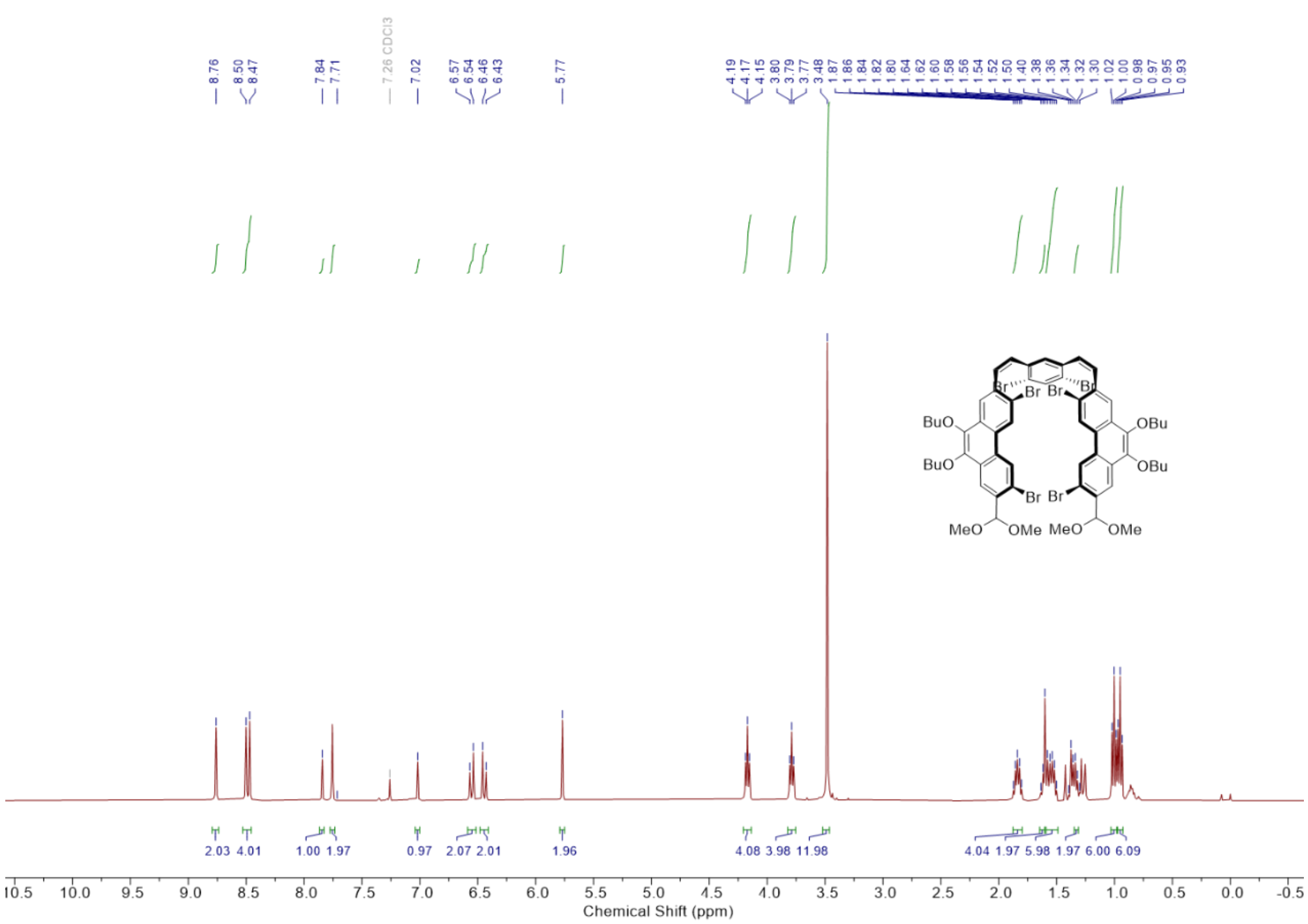

Fig. S25. ^{1}H NMR spectrum of 4 in $CDCl_3$ (400 MHz, 298 K).

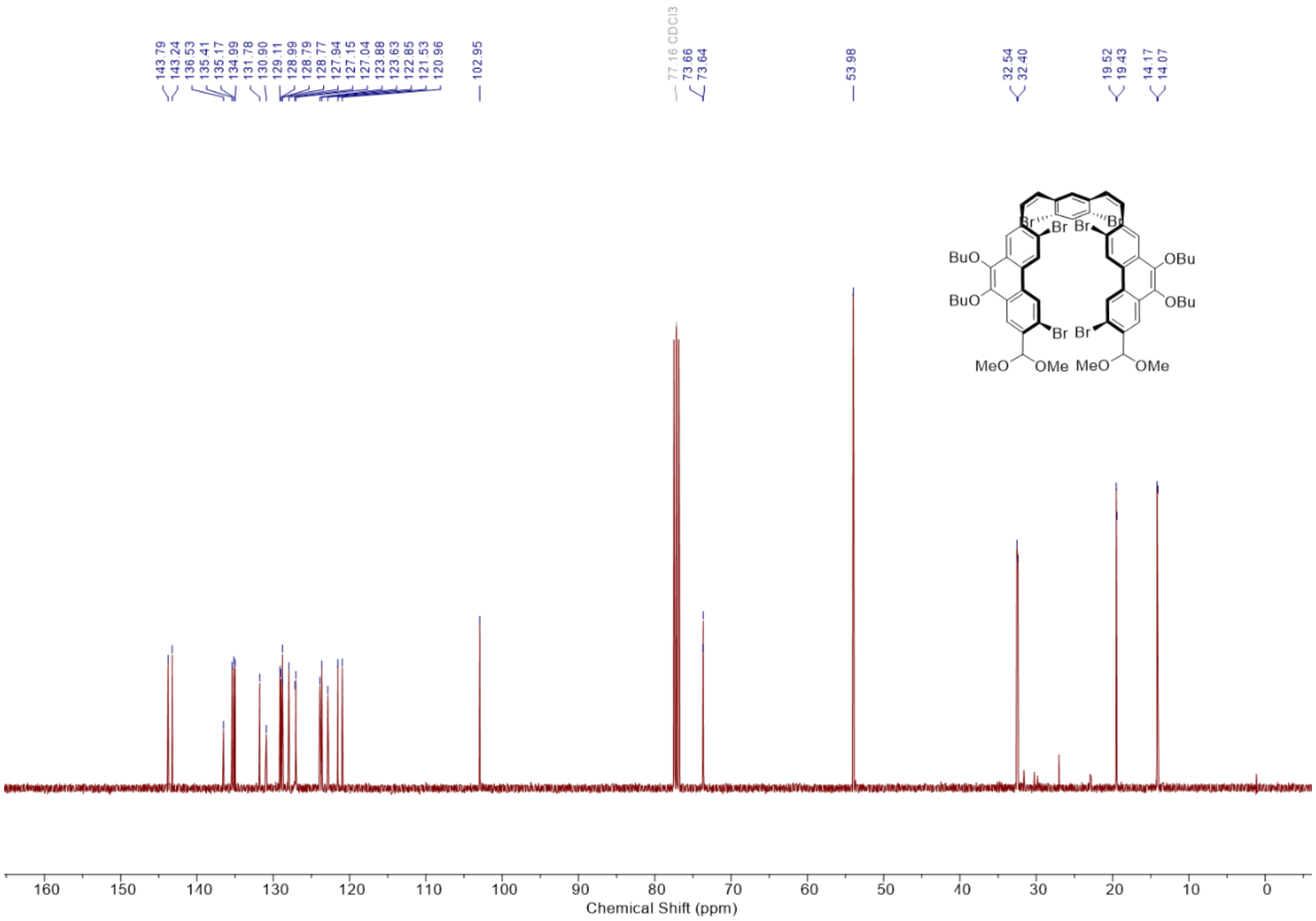

Fig. S26. ^{13}C NMR spectrum of 4 in $CDCl_3$ (100 MHz, 298 K).

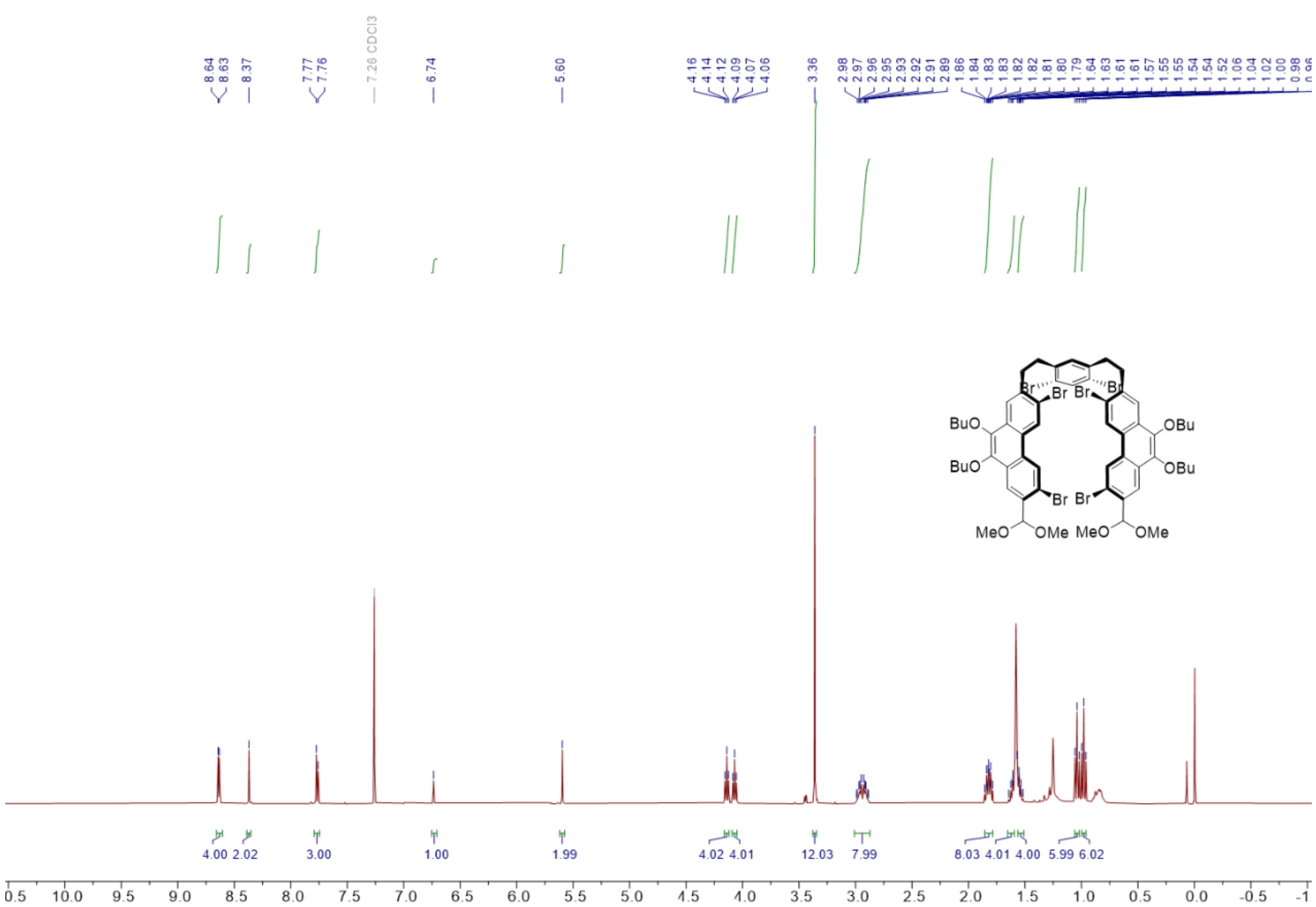

Fig. S27. ^{1}H NMR spectrum of 6 in $CDCl_3$ (400 MHz, 298 K).

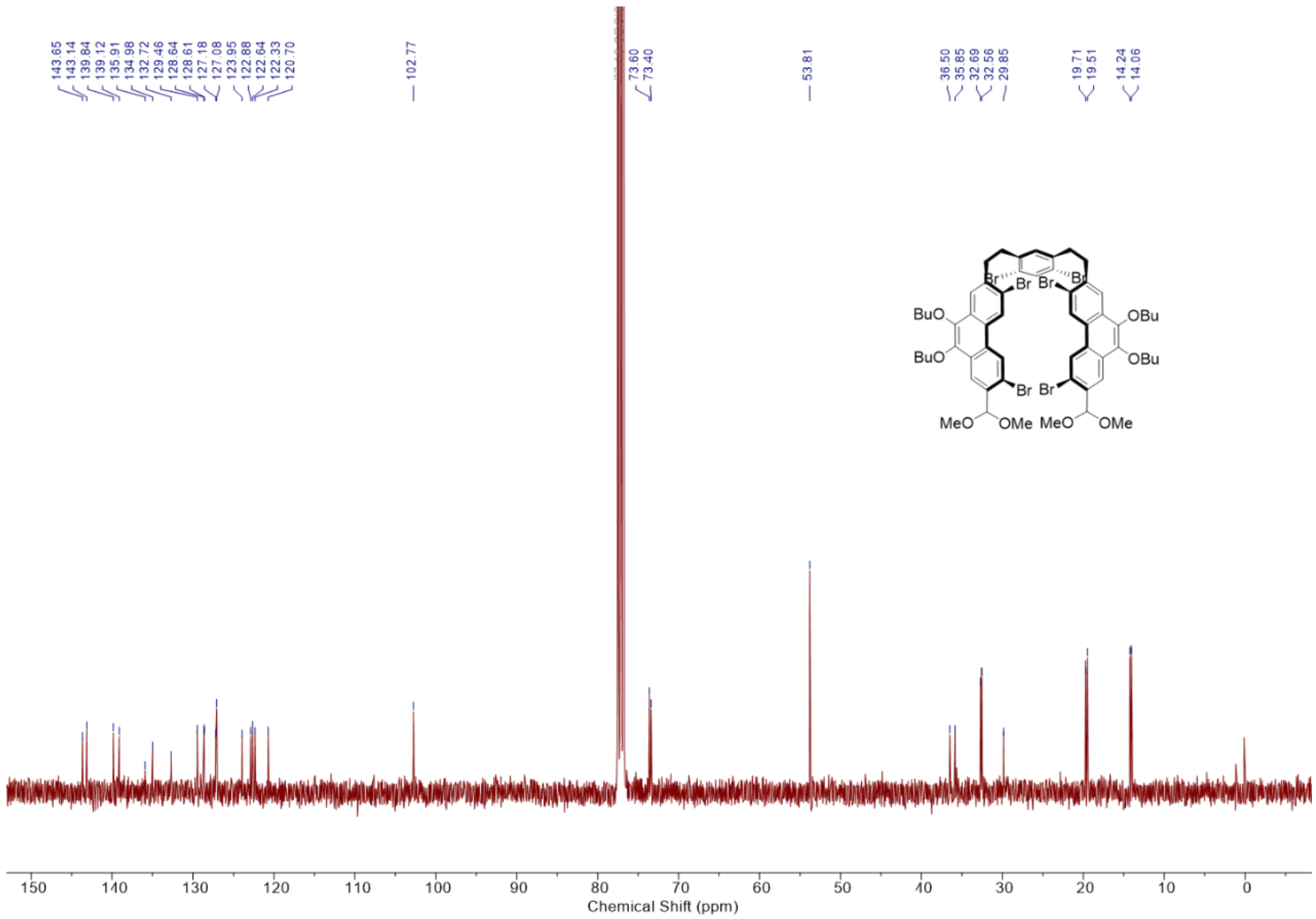

Fig. S28. ^{13}C NMR spectrum of 6 in $CDCl_3$ (100 MHz, 298 K).

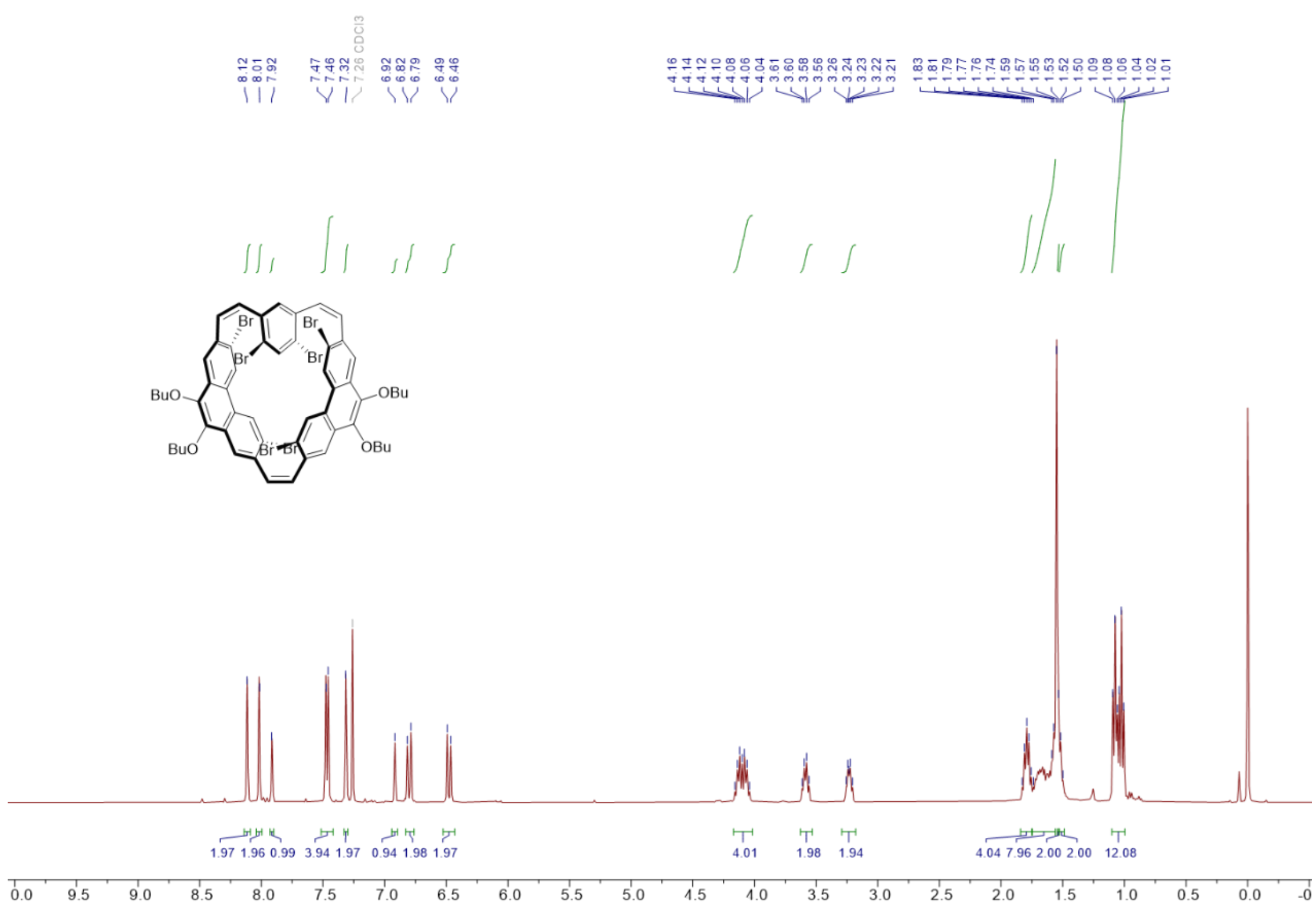

Fig. S29. ^{1}H NMR spectrum of 5 in $CDCl_3$ (400 MHz, 298 K).

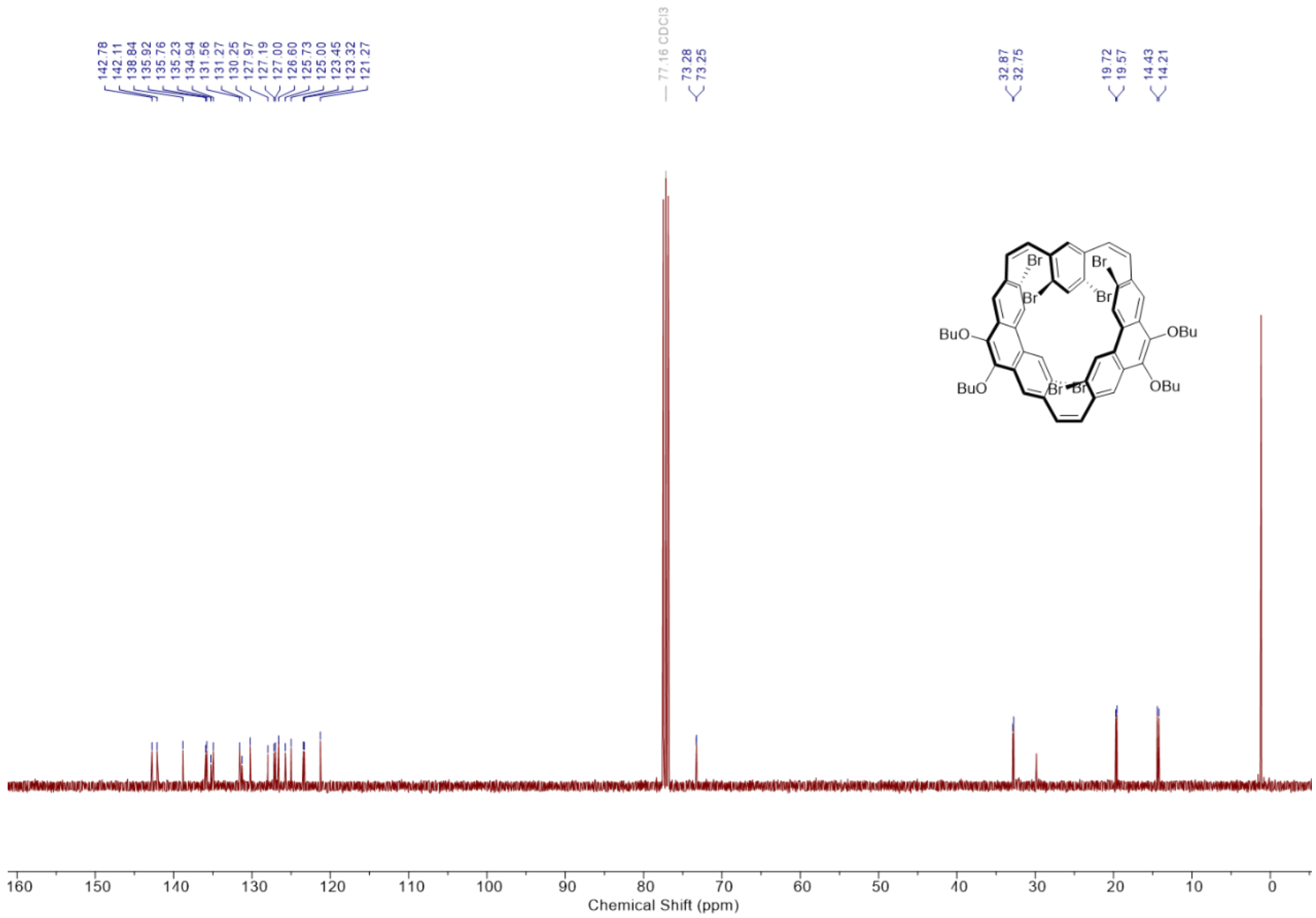

Fig. S30. ^{13}**C NMR spectrum of 5 in CDCl$_3$ (100 MHz, 298 K).**

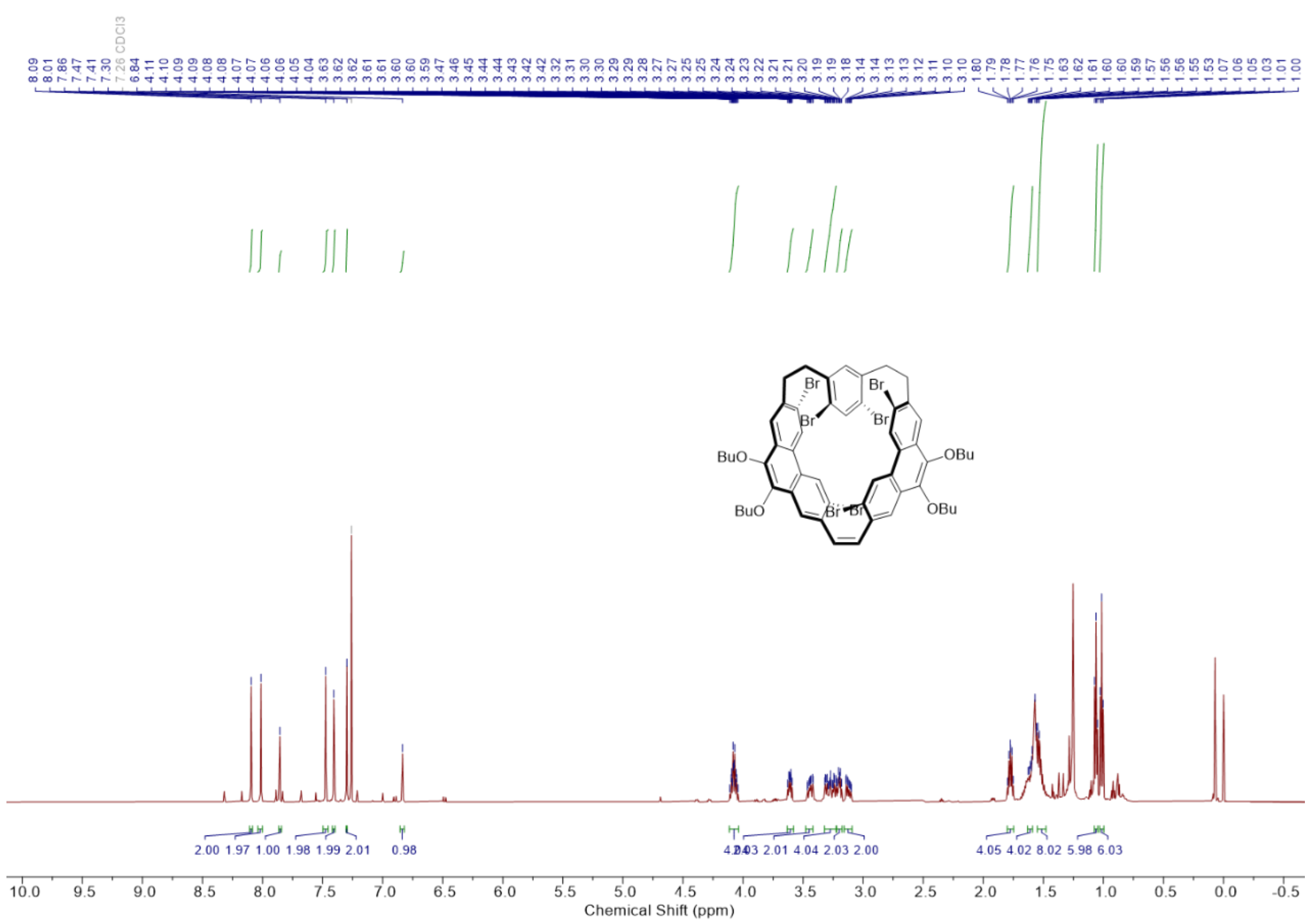

Fig. S31. ^{1}**H NMR spectrum of 7 in CDCl$_3$ (600 MHz, 298 K).**

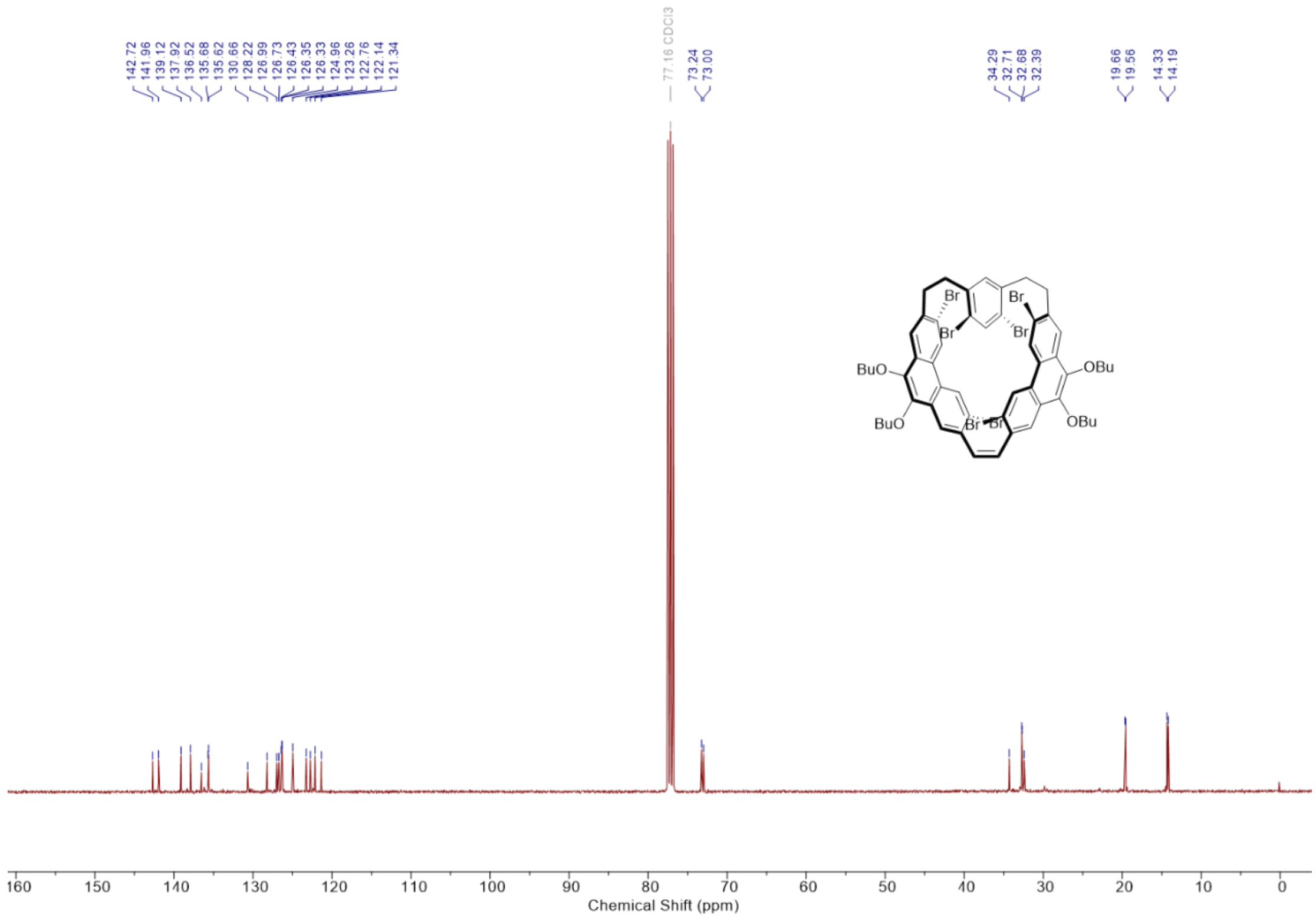

Fig. S32. ^{13}C **NMR spectrum of 7 in CDCl$_3$ (100 MHz, 298 K).**

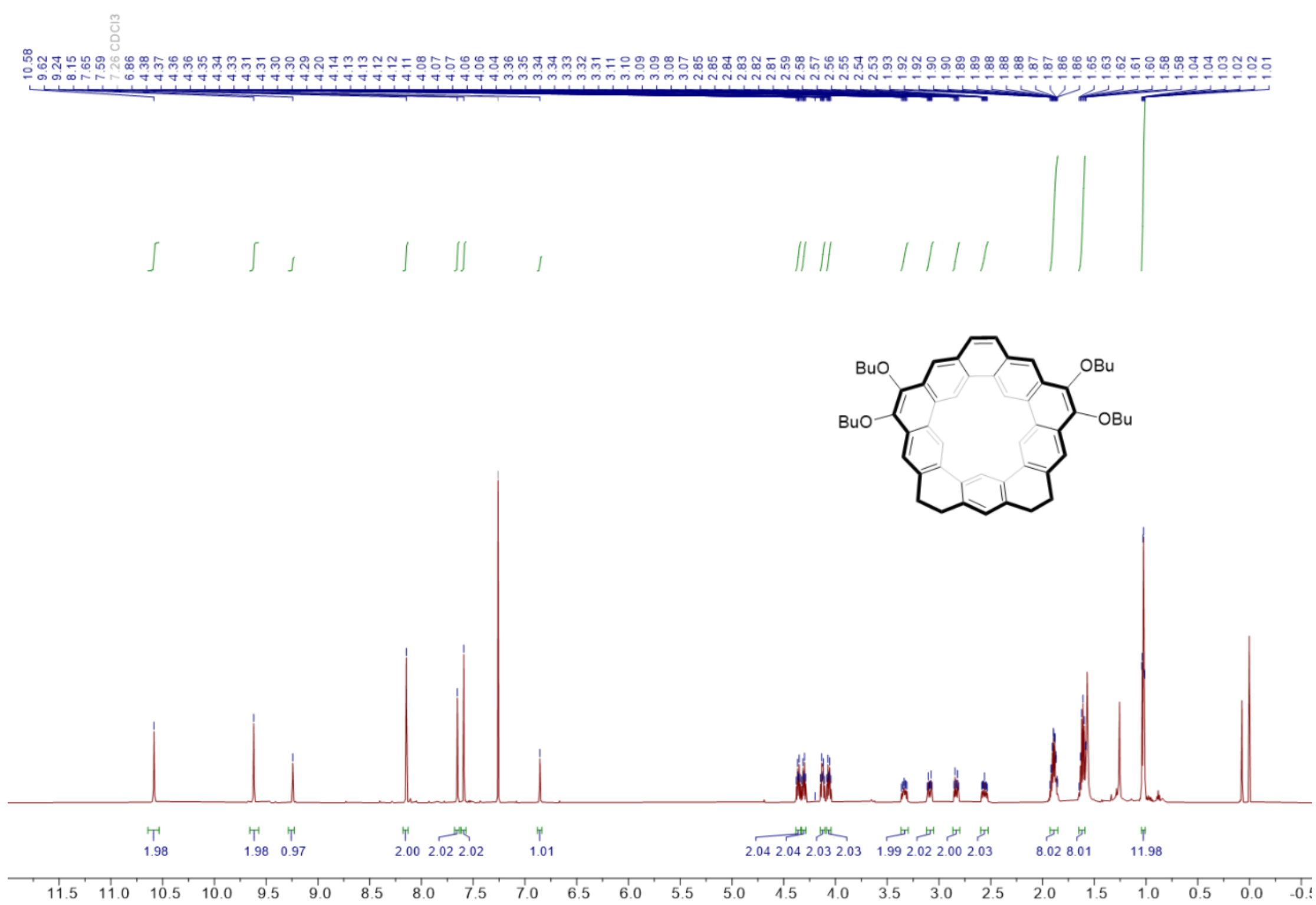

Fig. S33. ^{1}H **NMR spectrum of 8 in CDCl$_3$ (600 MHz, 298 K).**

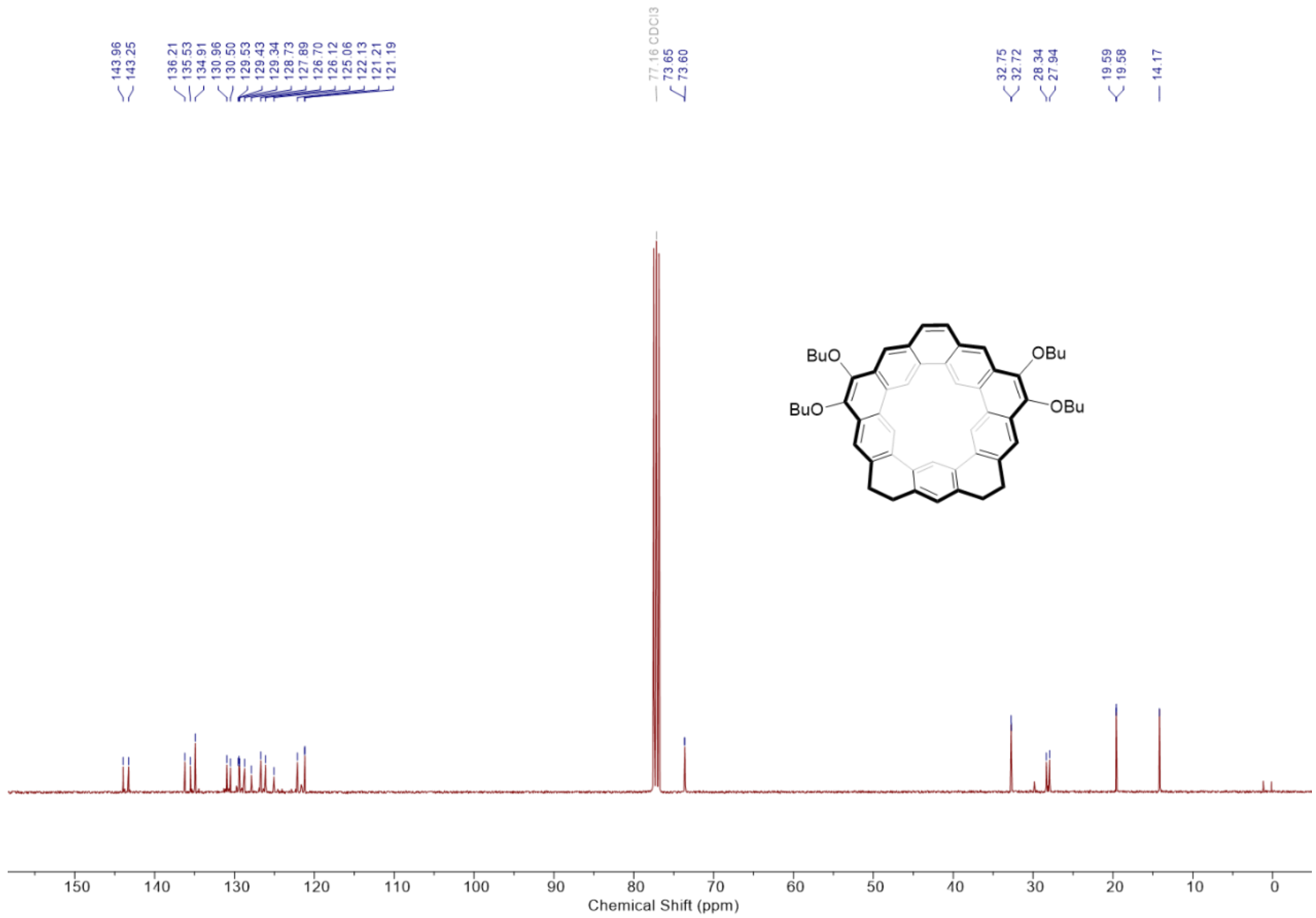

Fig. S34. ^{13}C NMR spectrum of 8 in $CDCl_3$ (100 MHz, 298 K).

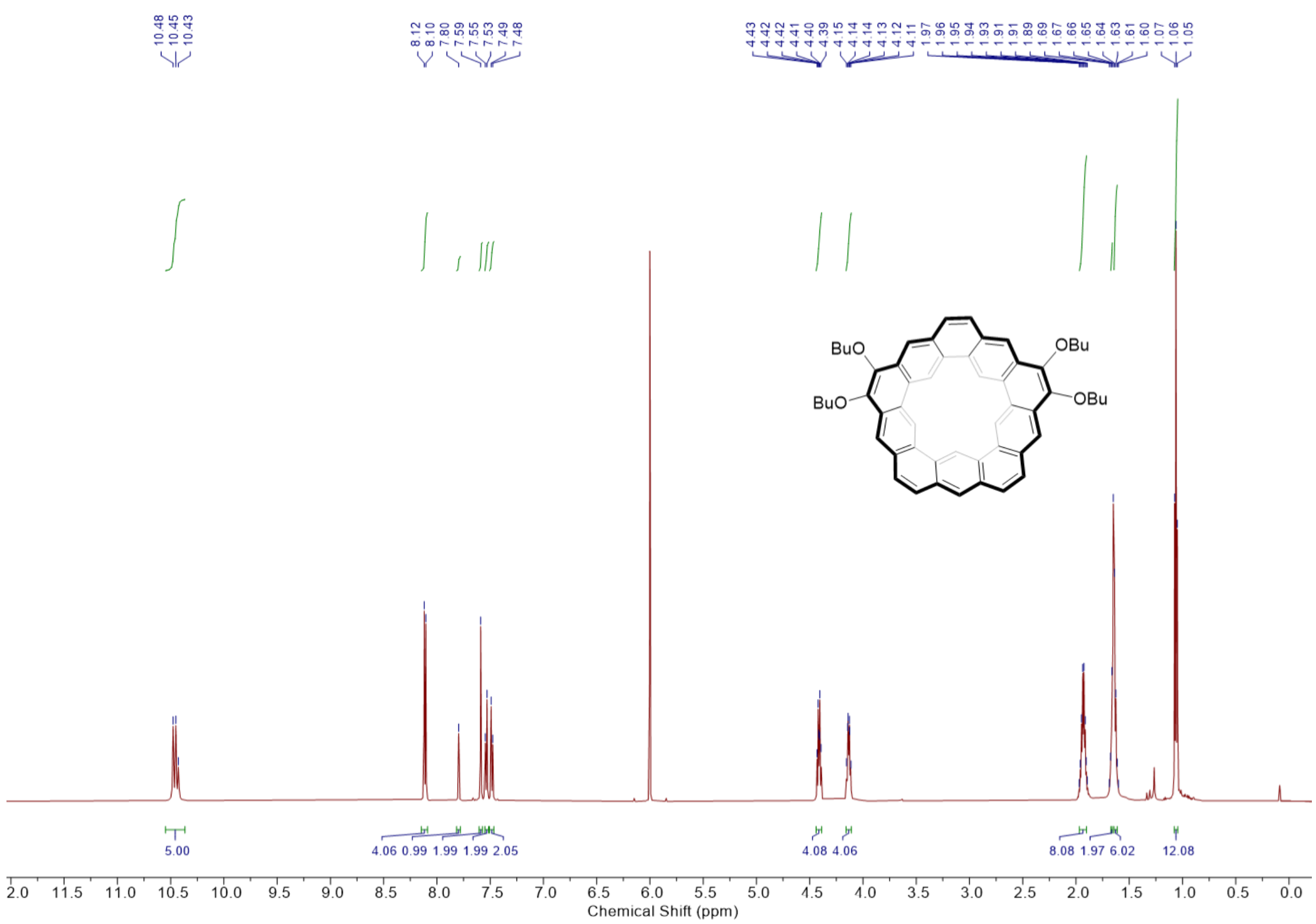

Fig. S35. ^{1}H NMR spectrum of 1 in $C_2D_2Cl_4$ (600 MHz, 298 K).

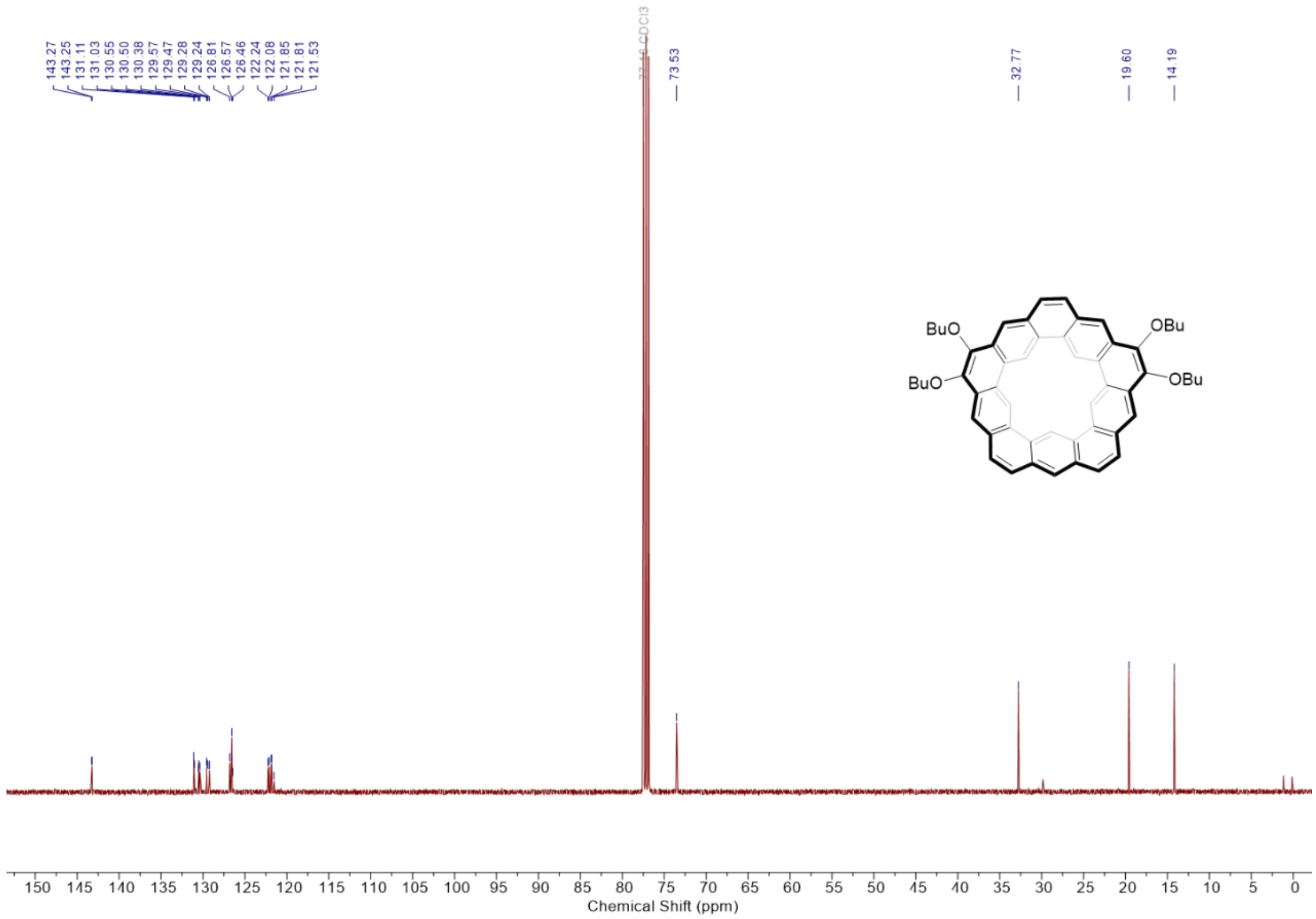

Fig. S36. ^{13}C NMR spectrum of 1 in $CDCl_3$ (100 MHz, 298 K).

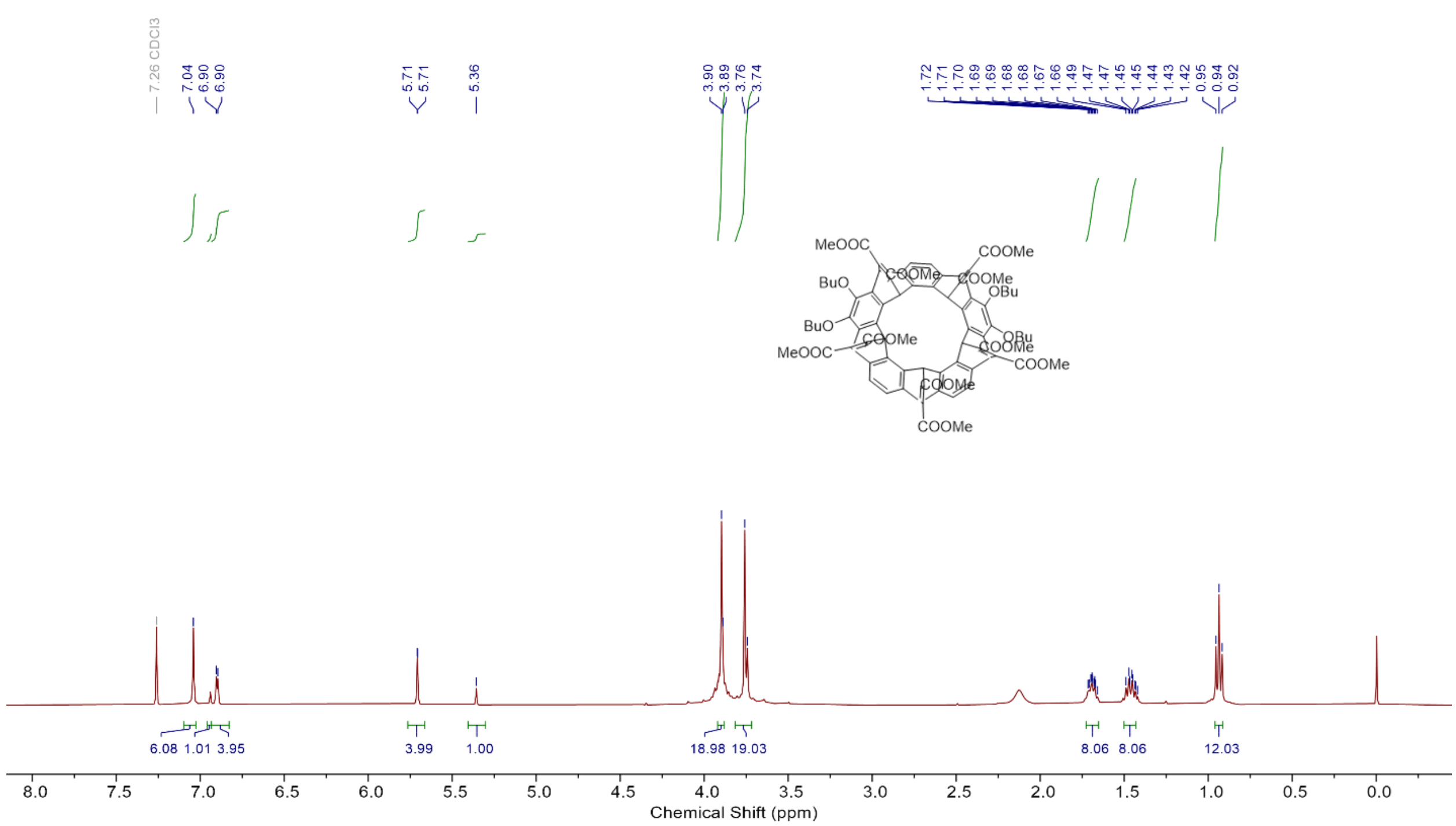

Fig. S37. ^{1}H NMR spectrum of 9 in $CDCl_3$ (400 MHz, 298 K).

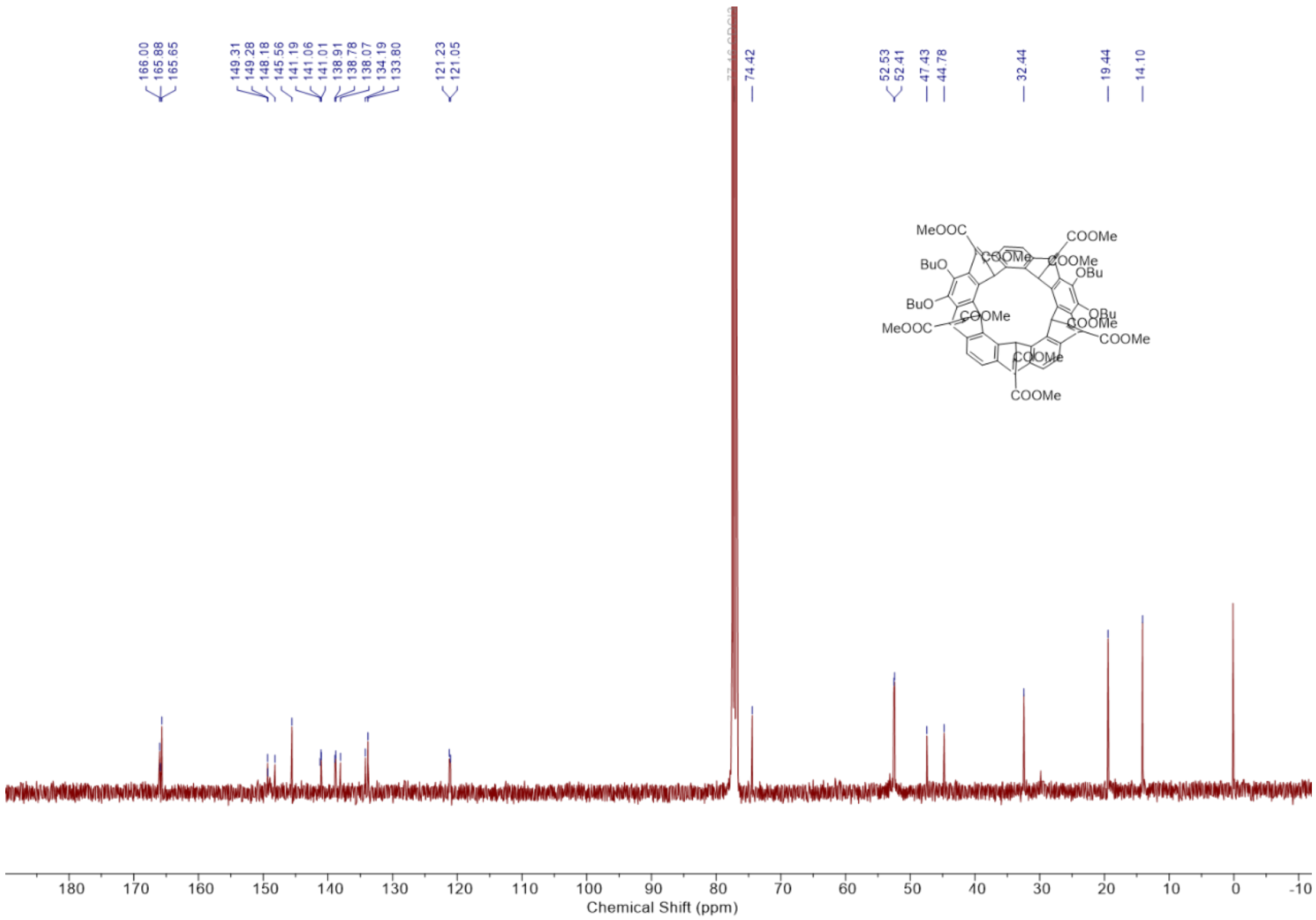

Fig. S38. ^{13}**C NMR spectrum of 9 in CDCl**$_3$ **(100 MHz, 298 K).**

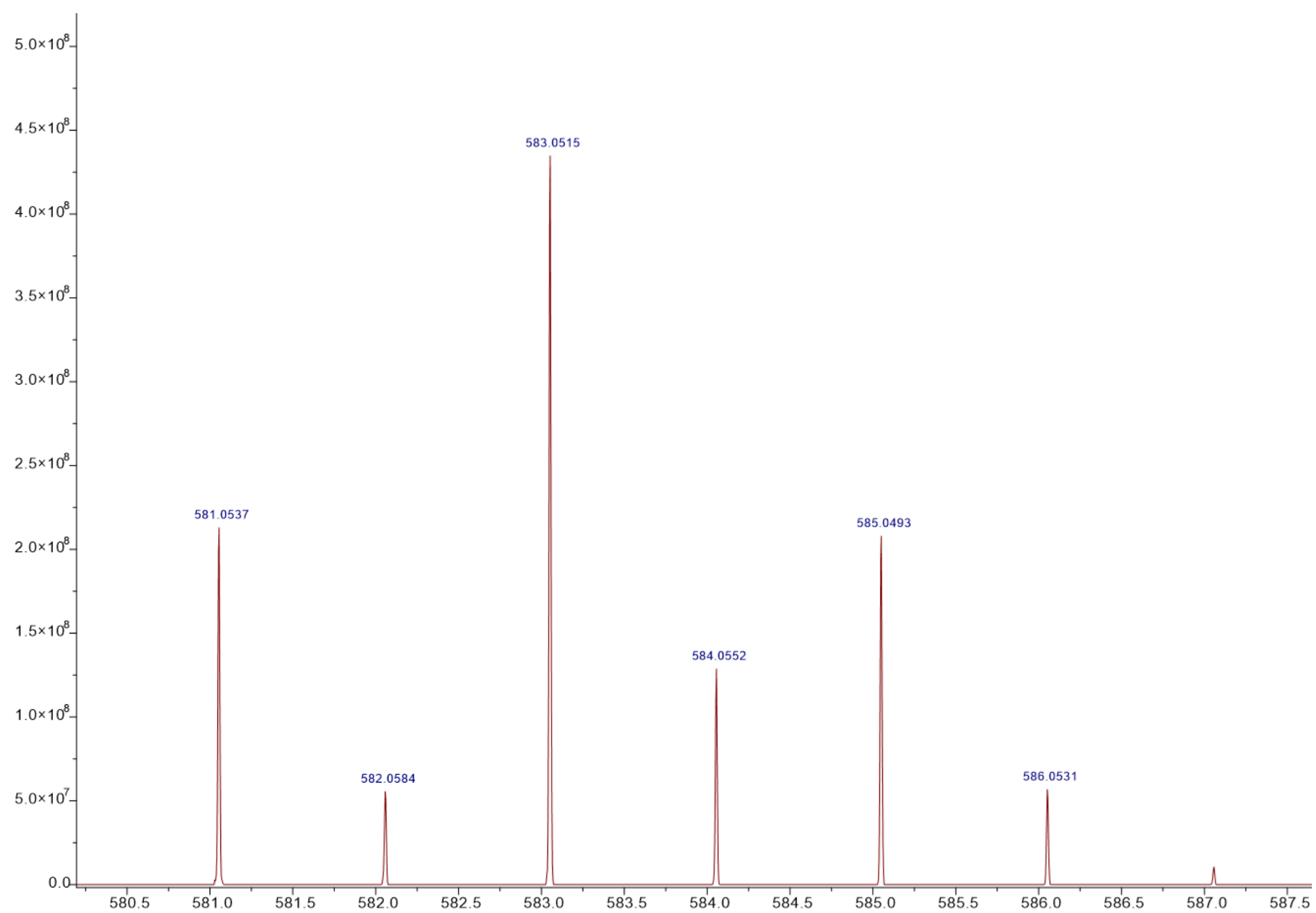

Fig. S39. HR mass spectrum (APCI) of 2.

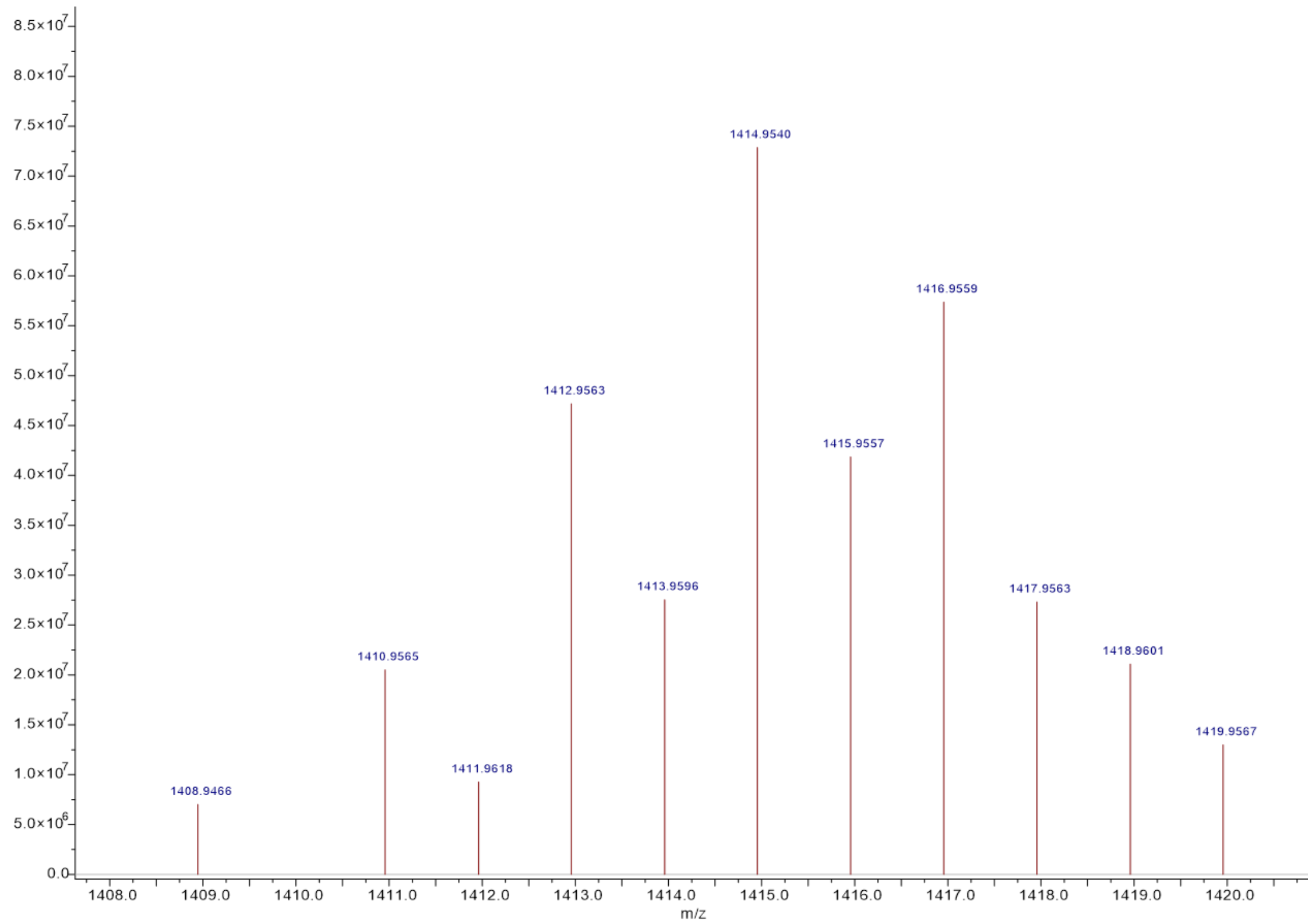

Fig. S40. HR mass spectrum (APCI) of 4.

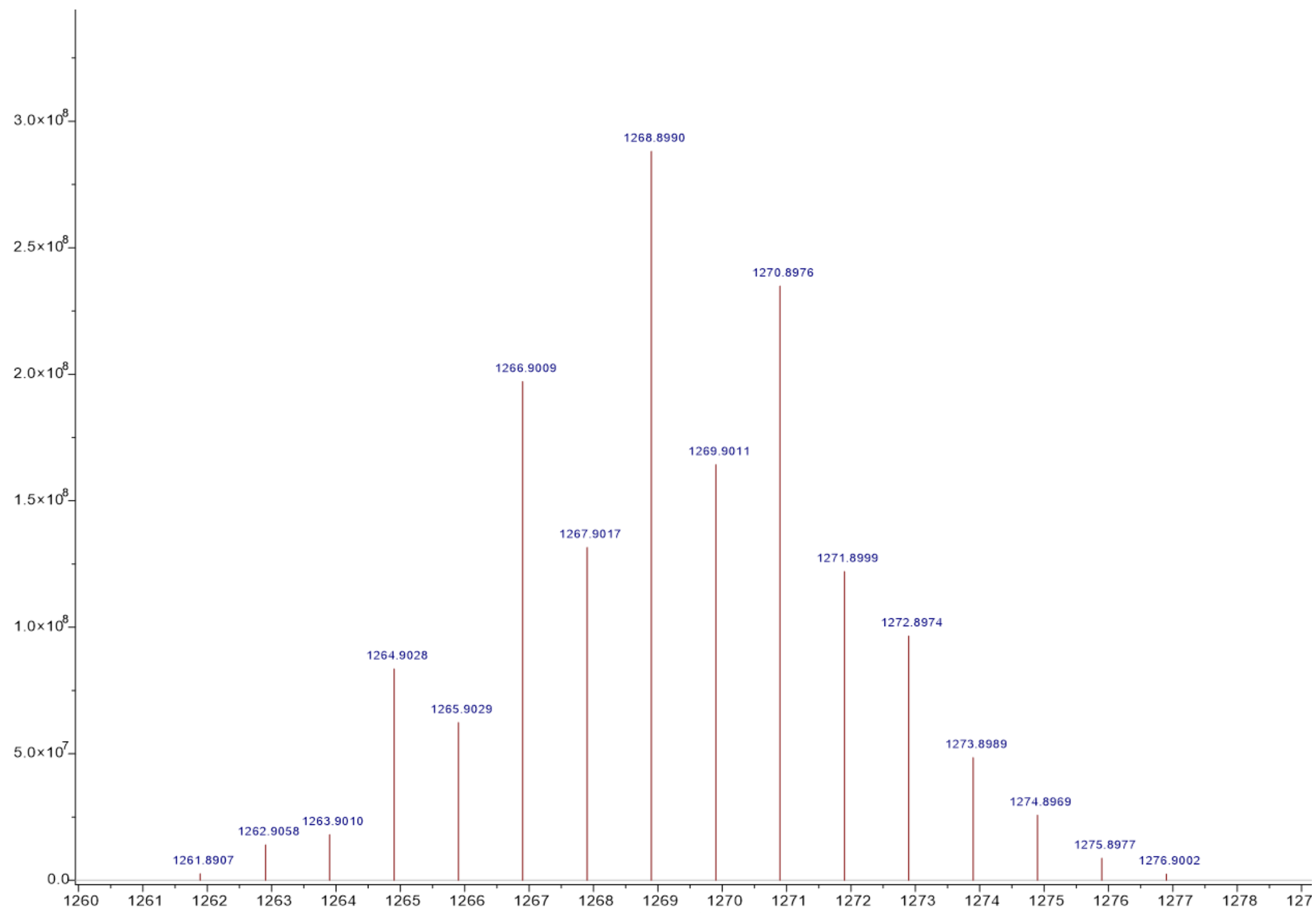

Fig. S41. HR mass spectrum (APCI) of 5.

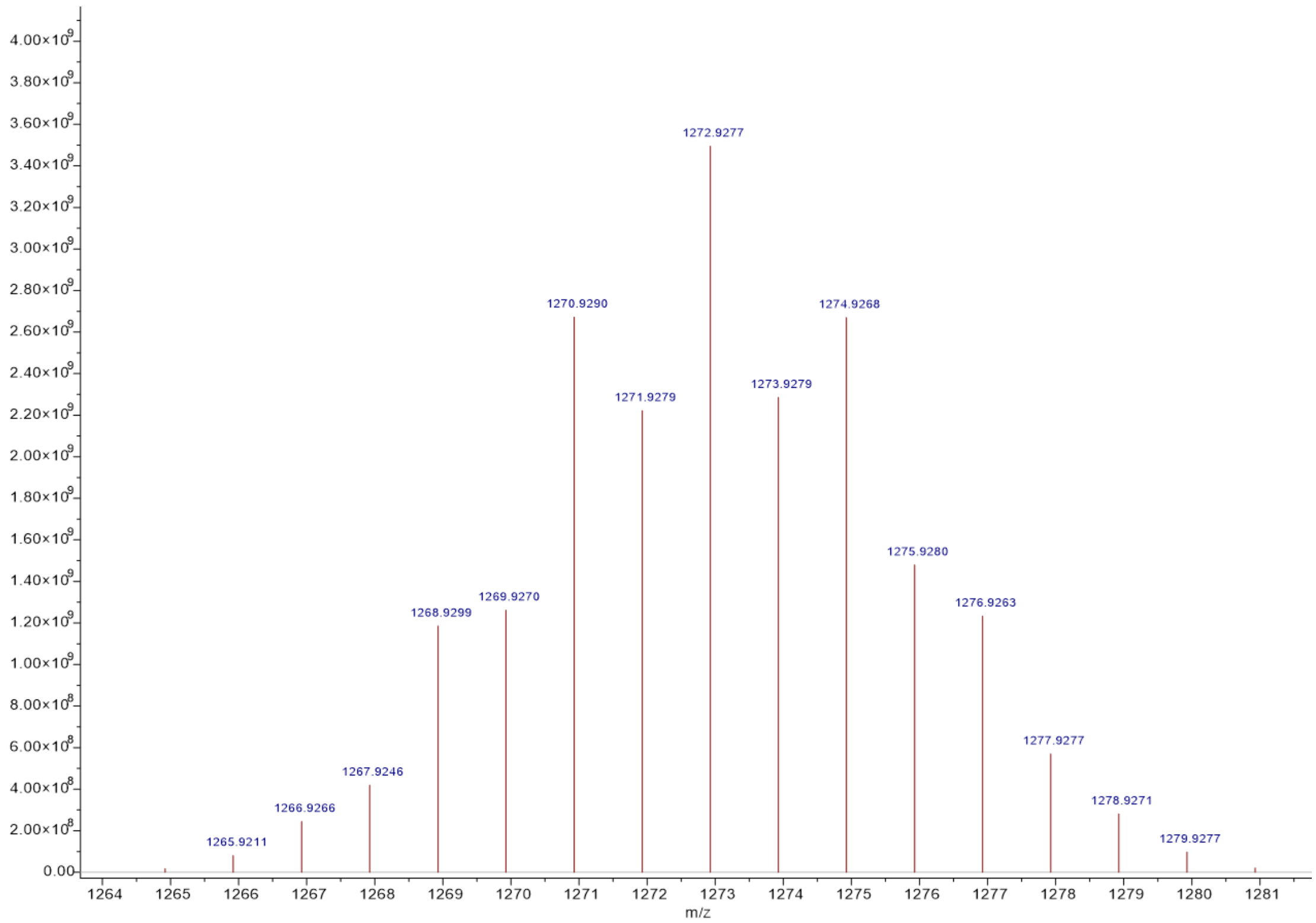

Fig. S42. HR mass spectrum (APCI) of 7.

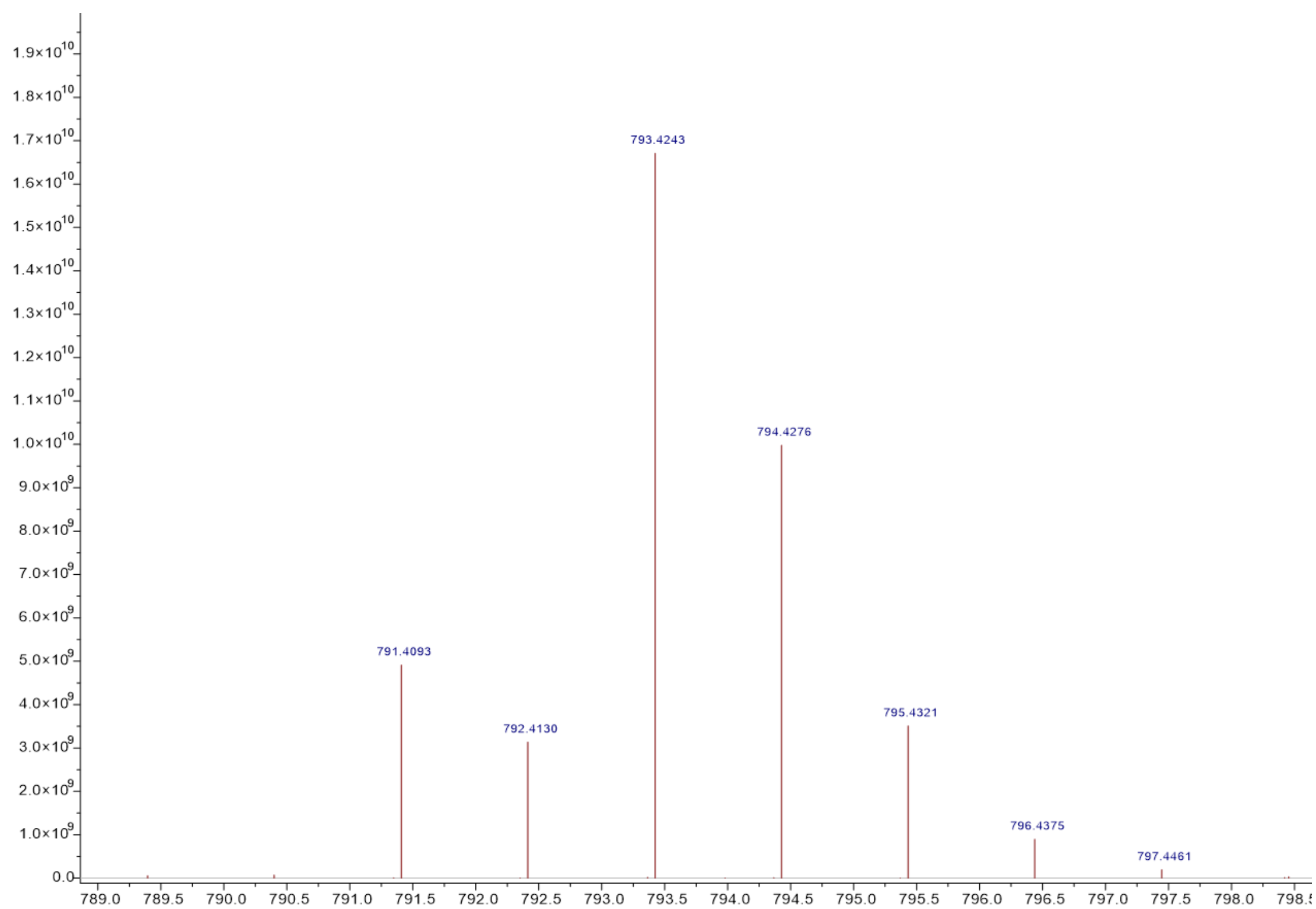

Fig. S43. HR mass spectrum (APCI) of 8.

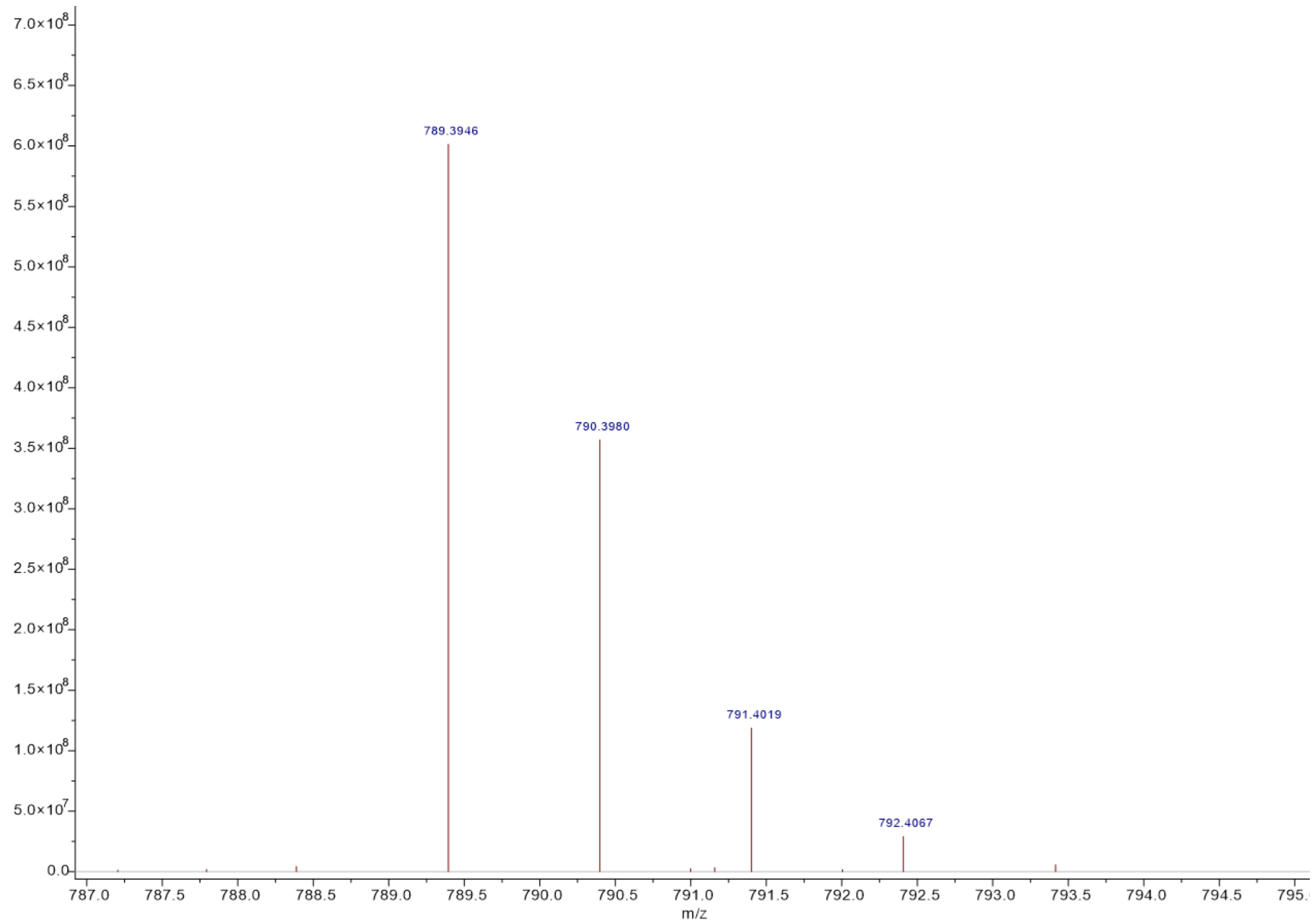

Fig. S44. HR mass spectrum (APCI) of 1.

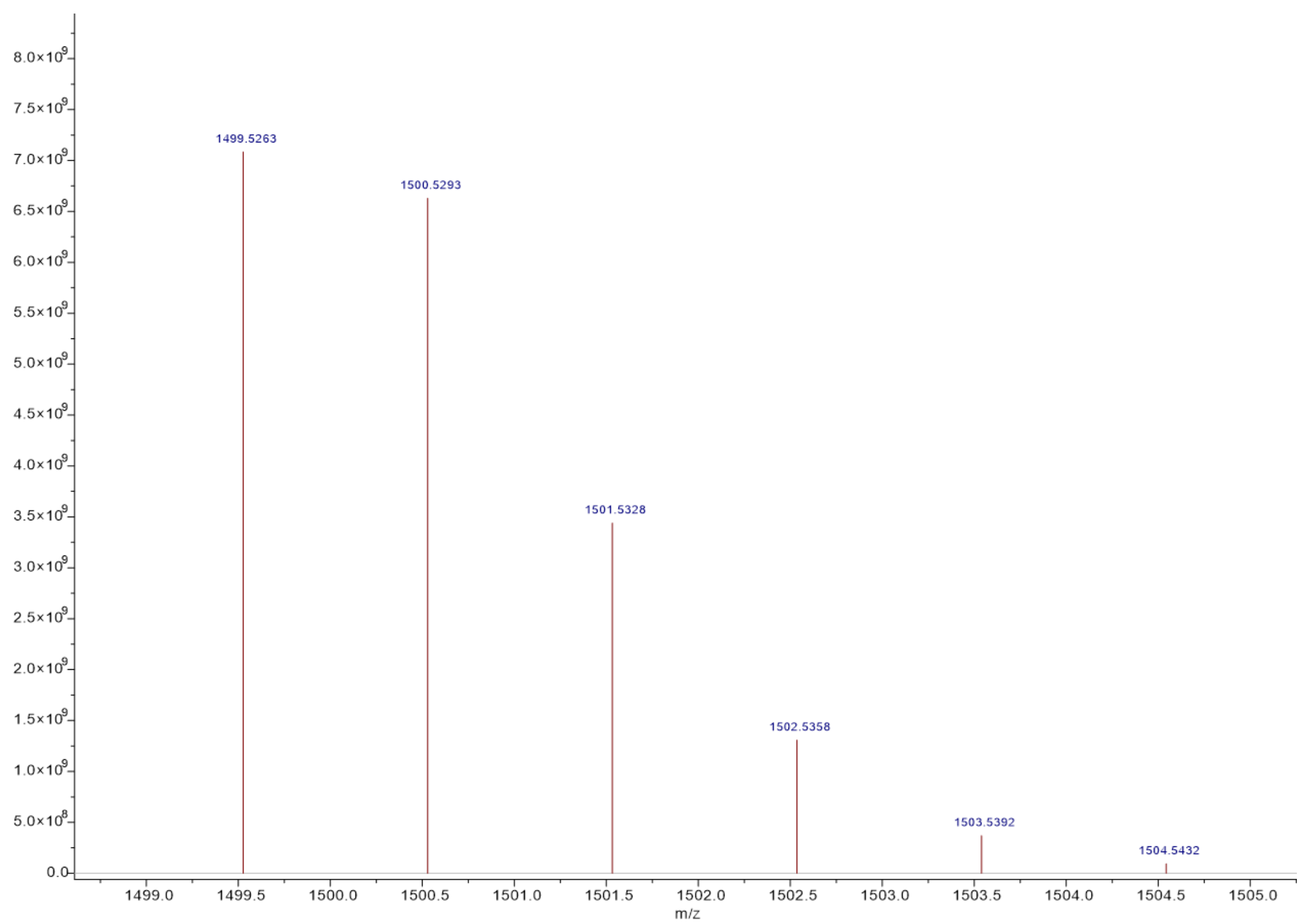

Fig. S45. HR mass spectrum (APCI) of 9.

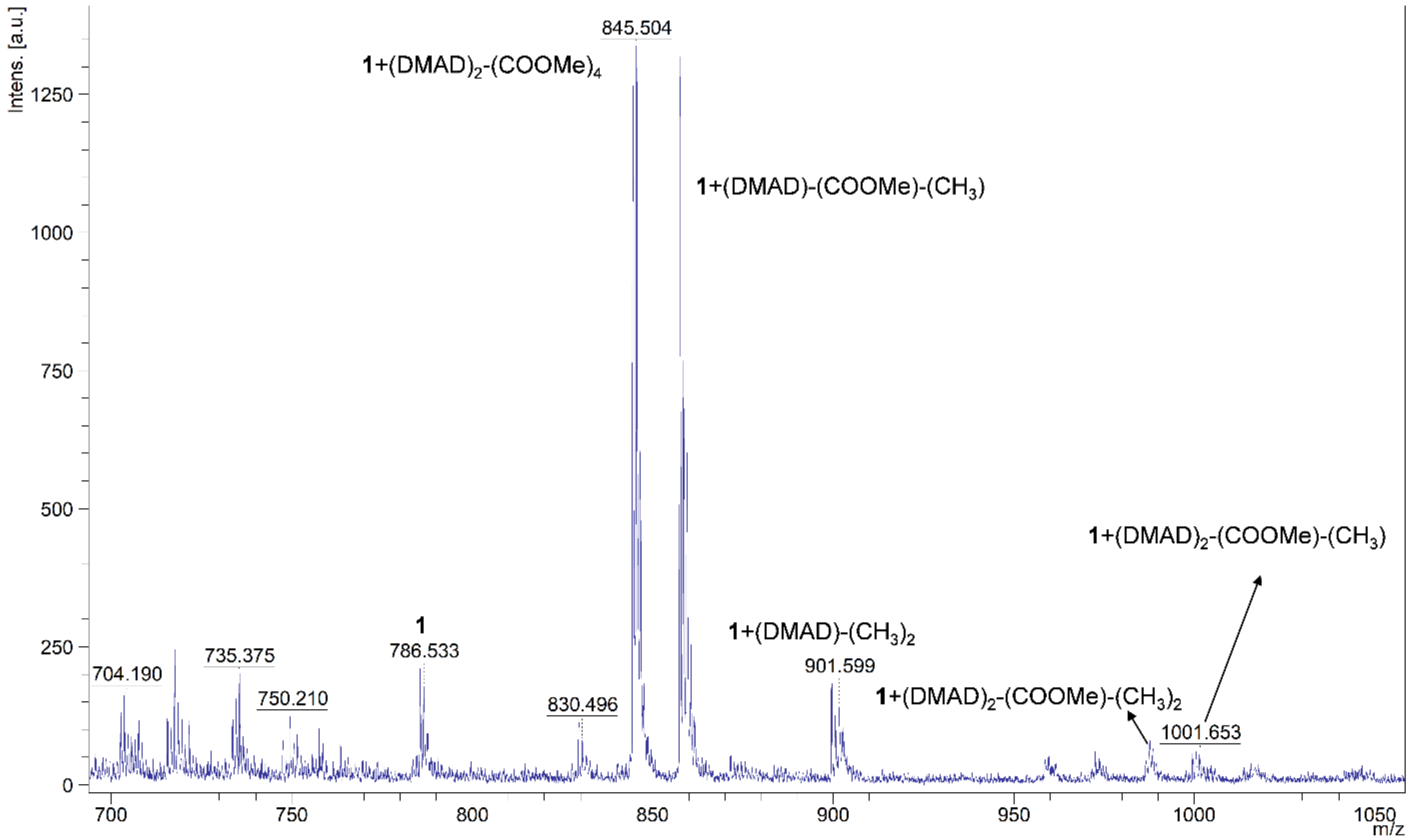

Fig. S46. MALDI mass spectrometry. MALDI spectra of the mixture generated from the reaction mixture of **1** with 2.5 equivalents of DMAD.

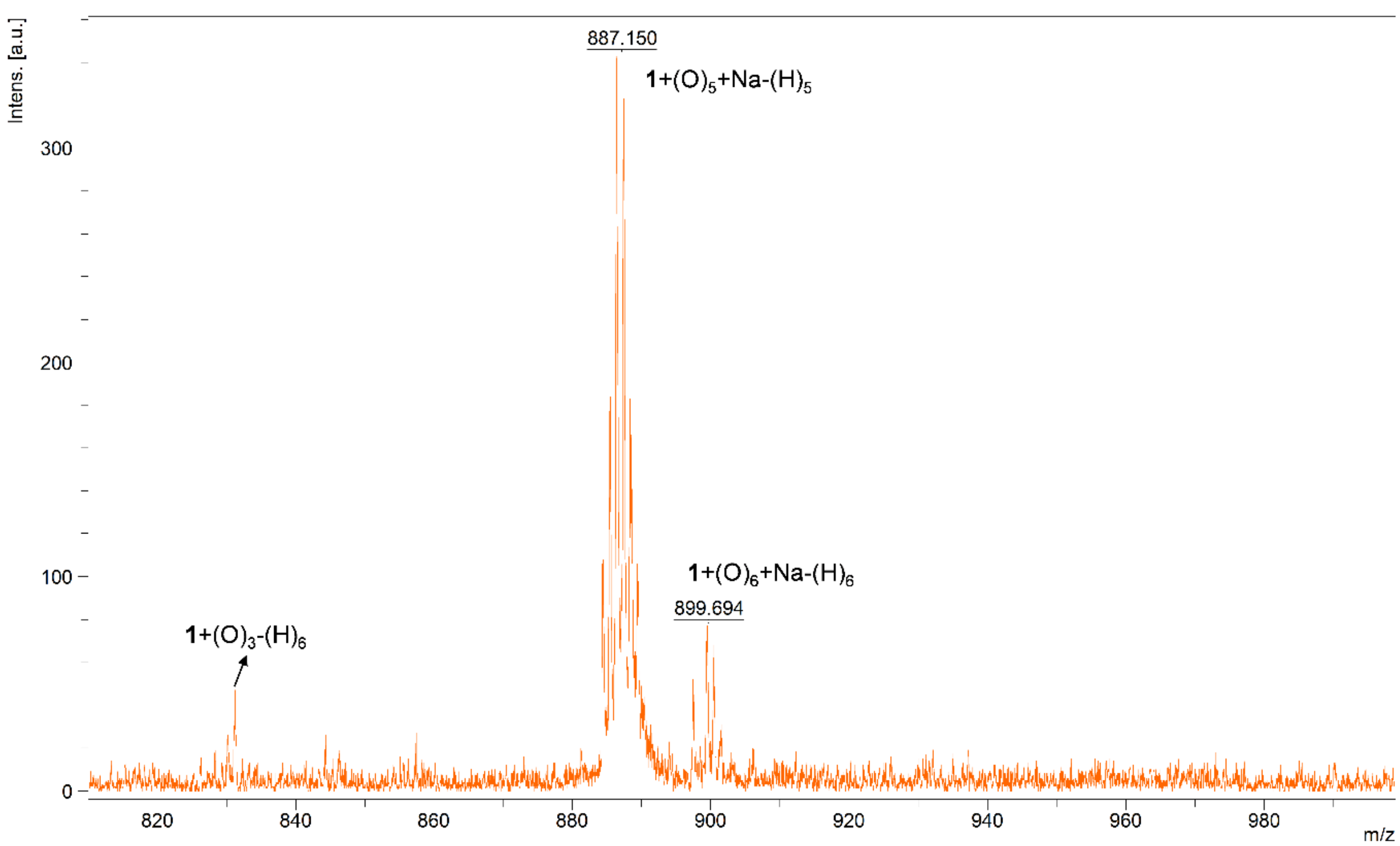

Fig. S47. MALDI MS spectra of overoxidation and the decomposition products.

7. Cartesian coordinates of optimized structures.

1

C	0.07363837	4.03439249	11.73476654	C	4.79475691	2.56364593	2.37413372
H	0.29616189	4.55413491	10.79547888	H	4.19679542	2.87766908	1.52240717
H	0.66680849	4.47111991	12.54253831	C	6.12732532	2.85448034	2.41002856
O	0.32508422	2.6266669	11.60475997	H	6.58359475	3.3985425	1.58713595
C	1.61621751	2.28838397	11.26268386	C	2.72517674	1.91675009	3.65402345
C	2.67050027	2.51181346	12.11724771	H	2.09950002	2.32669939	2.8640746
C	4.04024514	2.2529377	11.71269805	C	2.12447753	1.5474363	4.87397402
C	5.15408448	2.81387491	12.36678362	C	0.73794798	1.80880365	5.195232
H	4.98522533	3.39035865	13.27063879	H	0.07108327	2.11541226	4.39344709
C	6.43850509	2.78933305	11.78729548	C	0.27991779	1.76982541	6.47987109
C	6.6361439	2.04105355	10.58848088	H	-0.74924232	2.04536604	6.6950662
C	7.82751515	2.27374466	9.76854068	C	1.15549565	1.46292265	7.59047427
C	7.90478534	1.75580001	8.47745107	C	0.83862094	1.7526941	8.93265331
H	7.23407916	0.95765992	8.20665112	H	-0.15092628	2.12227311	9.18166301
C	8.6463473	2.3629368	7.46682428	C	1.81882339	1.71973202	9.94298597
C	8.24602148	2.21381109	6.0665635	C	3.11677717	1.21910163	9.62693534
C	8.82581094	3.04649288	5.06465988	C	3.33911855	0.73590121	8.33979243
C	9.95559844	3.87305534	5.44812653	H	4.30769613	0.32305022	8.11408765
O	10.58918114	4.57498031	4.4438627	C	2.45694868	0.96351376	7.28515535
O	2.48184095	3.11725063	13.34215397	C	2.95159049	1.01105529	5.9064832
C	1.82218157	2.29780714	14.31683978	C	4.30916573	0.83902664	5.64304548
H	2.395431	1.38272972	14.50690479	H	4.92645848	0.41761335	6.4177496
H	0.81098276	2.03500742	13.9920003	C	4.2458027	1.5111064	10.51215378
C	7.53838217	3.60680101	12.25317202	C	5.55216924	1.3429874	10.06039548
H	7.44103816	4.10299371	13.21529939	H	5.70073654	0.799208	9.14438395
C	8.63528279	3.84318274	11.47651883	C	9.68097344	3.75051962	9.18817654
H	9.40586683	4.52635577	11.82431016	H	10.40776203	4.50735499	9.46525618
C	8.76624754	3.27829082	10.15046727	C	11.04089827	6.30875168	6.89661897
C	9.58134577	3.37591728	7.83416183	H	10.13726896	6.63149667	7.42687188
C	10.29464257	4.05543124	6.76864846	H	10.92756501	6.50846111	5.82781481
C	8.2010756	3.13330504	3.80547	C	11.9385528	4.16227273	4.18818536
H	8.64363109	3.77798517	3.0529275	H	11.97322317	3.1051756	3.89923975
C	6.94241423	2.54669891	3.5657624	H	12.57306347	4.32268169	5.06499864
C	6.36736192	1.71838454	4.57558458	O	11.30141742	4.91832526	7.14304157
C	7.09929883	1.49426511	5.73930113	H	11.90356514	6.85137294	7.28829411
H	6.66038398	0.87473788	6.50132436	H	12.29265864	4.77604404	3.35749209
C	4.93871699	1.39480583	4.53149932	H	1.77471539	2.89142889	15.23199357
C	4.12393383	1.92516608	3.48624332	H	-0.99055653	4.1313467	11.95866604

9 with lowest energy

C	-6.27583976	0.34529973	-2.67008146	O	-2.18385706	-3.62252956	-5.54472154
C	-5.98556831	0.54276036	-1.18615303	C	-2.35247056	-5.04778486	-5.42823799
C	-4.91276472	-0.24388132	-0.73005733	C	-0.90843258	-1.11409675	-7.27226174
C	-4.28902249	-1.07483132	-1.85673883	O	-1.12431424	-0.34446565	-8.19029764
C	-3.89750038	-0.11062692	-2.98444116	O	-0.40798938	-2.35488078	-7.44341248
C	-4.98625058	0.67445681	-3.41768426	C	-0.20743161	-2.75111362	-8.80923515
C	-6.72420916	1.3419643	-0.3198117	C	-5.2888284	-3.39108083	-2.4589674
C	-6.434838	1.29898785	1.06652302	O	-4.23634809	-3.93322658	-2.74943706
C	-5.41832465	0.46298869	1.51643324	O	-6.39944377	-4.04211632	-2.09176352
C	-4.62181717	-0.2766022	0.62445252	C	-6.36390571	-5.47156792	-2.22343881
C	-5.10285774	0.15621743	2.97678993	C	-7.66321969	-1.7023795	-3.60029299
C	-3.62298283	0.4672563	3.1831456	O	-7.69092021	-2.72815897	-4.24878158
C	-2.79526982	-0.26157431	2.304742	O	-8.72102823	-0.86684513	-3.48399271
C	-3.59897007	-1.15232734	1.35333031	C	-9.91210123	-1.29051463	-4.16608123
C	-4.85957641	1.5705801	-4.46656826	H	-7.13517953	0.91852288	-3.00858696
C	-3.08832384	1.28596246	4.16510357	H	-3.47253374	-1.71410898	-1.52798448

C	-3.63374567	1.66532344	-5.14673095	H	-5.75642552	0.68009408	3.66987646
C	-2.57678868	0.8616153	-4.75320612	H	-2.97609309	-1.73577594	0.68149431
C	-2.68686573	-0.0129563	-3.65156547	H	-5.71187377	2.16192614	-4.78992443
C	-1.41965383	-0.85345276	-3.4516256	H	-3.74060926	1.83645836	4.83719617
C	-0.22329277	0.10179473	-3.34903578	H	-3.5301569	2.32789002	-6.00107855
C	-0.14403421	0.97564442	-4.45254711	H	-1.4836376	-1.55524417	-2.62279293
C	-1.24246022	0.72170089	-5.47766769	H	-1.1667478	1.35340975	-6.36198638
C	0.894431	1.88383245	-4.58500806	H	0.93419847	2.55063249	-5.44162713
C	1.9252558	1.89122691	-3.63224199	H	2.77127824	2.56326142	-3.74467871
C	1.87937147	0.99285523	-2.5780407	H	0.1862326	-1.53746166	-1.07876348
C	0.79310427	0.11165111	-2.40397251	H	3.8597675	1.39330184	-1.73230701
C	1.0072084	-0.84252466	-1.22410068	H	-0.74168803	-1.68848381	0.97759092
C	1.32524512	-0.00995816	0.02322927	H	1.03846532	0.95167287	4.49448458
C	2.41911494	0.85049263	-0.17528264	H	-1.25917871	1.94877543	5.11878528
C	3.00431715	0.7377369	-1.58070452	H	-9.64075073	7.05829304	-3.30401817
C	0.75921319	-0.10117547	1.28512844	H	-10.29807682	5.45817868	-3.67251845
C	1.28756728	0.67021604	2.33553862	H	-10.55233707	6.17062424	-2.07521717
C	2.35200491	1.54745665	2.14153938	H	-8.0660011	6.05994936	-1.63725005
C	2.93238648	1.64116438	0.84966541	H	-7.81376024	5.35166848	-3.2225742
C	-0.34761875	-1.04717346	1.76242818	H	-9.21094916	3.358444	-2.55418886
C	-1.42053671	-0.2107694	2.46957038	H	-9.45849923	4.0668074	-0.96451017
C	-0.88214274	0.58039735	3.50425093	H	-6.74092359	3.26577817	-2.13360692
C	0.61982826	0.37188399	3.67490485	H	-6.99220785	3.99195209	-0.53852219
C	-1.69351611	1.34574341	4.32649407	H	-7.82319918	6.70963017	5.41427951
C	-6.48106158	-1.17109128	-2.87006295	H	-9.15736811	5.94417792	4.54080622
C	-1.12737226	-0.76921327	-5.84887098	H	-8.3554166	5.0825791	5.8591258
C	0.77622112	-1.14958396	3.89681517	H	-6.22048819	5.01638478	4.50842329
C	-5.2324581	-1.37824045	3.11429474	H	-7.01665611	5.87221802	3.19998989
C	3.35687508	-0.75546099	-1.76152794	H	-8.63151859	3.95343512	2.92356375
C	-5.44901037	-1.91225311	-2.42139174	H	-7.83723889	3.09738213	4.23744865
C	-1.24753272	-1.59588899	-4.79330831	H	-6.50327385	3.91642882	1.60312107
C	2.31814485	-1.58449457	-1.54962478	H	-5.70510543	3.04303863	2.91944927
C	0.293825	-1.89095843	2.87935491	H	5.96481525	2.75591952	8.11214001
C	-4.42990734	-2.06232746	2.27753606	H	5.62861466	4.12109719	7.03856378
O	-7.79139356	2.08174403	-0.77518601	H	4.31146584	3.35917681	7.93763482
C	-9.82712341	6.06263986	-2.88934986	H	4.65241998	1.22746578	6.62167493
C	-8.5315106	5.41354692	-2.39343893	H	5.96440567	1.98331728	5.73311536
C	-8.75374266	4.01547945	-1.80351915	H	4.34494984	3.68113596	4.79668441
C	-7.46578392	3.37195272	-1.31303912	H	3.03878384	2.92085304	5.6965906
O	-7.22776719	1.99392101	1.9514225	H	4.71031192	1.56221477	3.52133999
C	-8.18648666	5.75820445	5.0132601	H	3.40180958	0.78877625	4.4347212
C	-7.19179854	5.15757452	4.01543839	H	9.65826507	3.0827062	-0.74761675
C	-7.6678802	3.82200218	3.43090136	H	8.33710284	3.55798481	-1.82282564
C	-6.67814476	3.22677277	2.44119815	H	8.61158501	4.44239682	-0.31746551
O	2.80833053	2.34246153	3.16451963	H	8.0170767	2.37015038	1.00075002
C	5.24251468	3.15954193	7.39524179	H	7.73871756	1.49084464	-0.49330998
C	5.00827149	2.19022094	6.23279537	H	5.87571895	3.0663672	-1.08890564
C	4.000773	2.72312165	5.2063484	H	6.13935246	3.9561825	0.40463473
C	3.77232292	1.74990873	4.06027421	H	5.33933314	1.00250727	0.23064989
O	3.95440457	2.52543566	0.60566471	H	5.59805959	1.91291658	1.73235585
C	8.6254938	3.44513666	-0.77158149	H	-3.40129113	-5.43053298	-0.26391615
C	7.68104566	2.48798491	-0.03841434	H	-2.599536	-5.46612999	1.34210921
C	6.22222617	2.96191728	-0.0523786	H	-4.3053796	-5.97188712	1.1954239
C	5.29745538	2.00125875	0.67982254	H	-7.2813629	-0.99280368	6.94832324
C	-4.20758822	-3.53637417	2.23624285	H	-8.28846219	-1.79093867	5.69521728
O	-4.19705744	-4.28038158	3.19070292	H	-6.98351633	-2.70122921	6.4861828
O	-3.94502855	-3.92622638	0.96660249	H	2.19215944	-5.72919681	3.8258613
C	-3.53768644	-5.29699986	0.80915331	H	0.41300241	-5.84085553	3.6128344
C	-6.20872141	-1.98656294	4.05778293	H	1.47027595	-5.70804903	2.18676511
O	-6.82569901	-3.01197941	3.87224235	H	0.62310654	-3.86455258	7.3349123
O	-6.35273398	-1.21446488	5.16343006	H	2.29993729	-3.50563586	6.80471035
C	-7.28945947	-1.71417815	6.13148169	H	1.38287898	-2.28329664	7.71372489

C	0.29716743	-3.36407291	2.70177778	H	6.15526625	-2.56965851	-4.59486582
O	-0.51391432	-3.95156069	2.00596021	H	6.94046982	-1.36518123	-3.51821441
O	1.31121599	-3.97459416	3.33987473	H	6.51670527	-2.97793096	-2.88771459
C	1.33948692	-5.40746746	3.22899557	H	0.3844506	-5.28881035	-0.477853
C	1.4044256	-1.60175746	5.16671242	H	0.64429824	-5.32095693	-2.25025266
O	2.38367815	-1.06696786	5.65401231	H	2.04503681	-5.50060689	-1.14272705
O	0.73452884	-2.60745174	5.75386615	H	-3.06555345	-5.31841275	-6.20649849
C	1.30460082	-3.0911639	6.9826317	H	-1.40104747	-5.56224985	-5.57999126
C	4.76854559	-1.12758143	-2.06428655	H	-2.75380243	-5.28888046	-4.44182702
O	5.69982541	-0.84394395	-1.33382001	H	0.21440313	-3.75469916	-8.76372802
O	4.89637113	-1.72527029	-3.25529669	H	-1.15623689	-2.75719426	-9.35153075
C	6.22069198	-2.18467158	-3.57796597	H	0.48116483	-2.06669895	-9.31038045
C	2.40226731	-3.06626958	-1.53938339	H	-7.33722401	-5.81911677	-1.87972701
O	3.41774745	-3.71145694	-1.7165839	H	-6.20655538	-5.75024467	-3.26812653
O	1.19414618	-3.60119724	-1.26326776	H	-5.56709198	-5.89767324	-1.61003687
C	1.07025214	-5.03263301	-1.28682628	H	-10.65296489	-0.51600487	-3.97045267
C	-1.26011182	-3.0865149	-4.73517374	H	-9.72695799	-1.38720972	-5.23876892
O	-0.56259575	-3.71930865	-3.9667974	H	-10.2524836	-2.25399793	-3.77929681

The transition state of **1**

C	4.63212047	2.01329761	0.10767034	C	1.2941489	-1.31311123	-0.05034223
C	4.39407527	0.61804633	0.19743095	C	1.11093157	3.29271515	0.59015975
C	3.57252591	2.95659223	0.2714972	C	-0.1106131	2.63840396	0.54792476
C	5.31711489	-0.49209952	-0.03223912	C	-1.26451082	3.25696783	0.12708915
C	4.92393131	-1.82918595	0.03763216	C	-2.3810568	2.41507563	-0.22072241
C	3.52117052	-2.22908302	0.11542995	C	-2.1067276	1.12592429	-0.61691377
C	2.95115569	-3.5301357	0.03008995	C	-3.07710098	0.13717123	-0.65501588
C	1.54198319	-3.71535215	-0.08737528	C	-2.63296452	-1.23947349	-0.75502557
C	3.61168571	4.38506442	0.00163406	C	-1.2974969	-1.485605	-1.03794144
C	0.75699318	-4.93641879	0.05245052	C	-0.66700553	-2.62477091	-0.57804339
C	2.49180328	5.18791421	0.01549418	H	5.62713899	2.36277762	-0.15131408
C	1.15255852	4.66872618	0.23677001	H	3.60943255	-4.39343215	0.05497878
C	-1.33026492	4.66739181	0.03434273	H	4.56863648	4.83616973	-0.24932286
C	-0.08998135	5.35817635	0.06584822	H	1.27926436	-5.87366505	0.22806599
C	-2.70382848	5.16157012	-0.06303871	H	2.60815678	6.24355762	-0.21766361
C	-3.80833453	4.33218833	-0.06830574	H	-0.07302855	6.43502782	-0.0883996
C	-3.71692046	2.87391706	-0.1457288	H	-2.86978277	6.23603558	-0.07766889
C	-4.40889492	0.49343181	-0.31270294	H	-4.79393675	4.79116939	-0.06803265
C	-4.72971381	1.87856316	-0.16596335	H	-5.76905142	2.15320079	-0.01390884
C	-3.44230425	-2.30847406	-0.28289975	H	-3.46918882	-4.41947885	0.20042824
C	-4.8242435	-1.94619844	0.00110071	H	-1.13204897	-5.87766516	0.28328291
C	-5.29305622	-0.63846988	-0.07531816	H	8.16705136	-0.12804297	-1.58808304
C	-1.43192421	-3.75486717	-0.1880326	H	7.15816493	-1.60327724	-1.68247979
C	-2.83819956	-3.58492471	-0.08980089	H	6.55241536	-0.05821291	-2.34656983
C	-0.6222003	-4.94033969	0.07437261	H	7.41843727	-3.82081001	0.73035255
O	6.63242241	-0.15300478	-0.2605899	H	7.44250578	-2.05266846	1.00421555
C	7.14808447	-0.51805207	-1.54956151	H	6.31064777	-3.08734364	1.92285178
C	6.80972648	-2.94339544	0.95748839	H	-6.73966669	-2.06338879	1.90308576
C	-6.13863971	-2.95492355	1.70428754	H	-6.74953225	-3.85126335	1.82872032
O	5.84498444	-2.84469148	-0.09989369	H	-5.29430529	-2.99119546	2.40259673
O	-5.67242879	-2.97680355	0.34681299	H	-7.54828816	-1.922674	-0.81062293
O	-6.61140947	-0.3478436	0.2056893	H	-8.53677432	-0.48296431	-0.42218824
C	-7.55772328	-0.83027951	-0.75830998	H	-7.34605439	-0.41421708	-1.7503878
C	3.08292855	0.20153731	0.54043819	H	1.2376555	0.75061965	1.39833135
C	2.15455056	1.1242146	0.97231142	H	0.65330299	-0.44971542	-0.07579024
C	2.30550847	2.47712378	0.70353552	H	-0.17054798	1.58723407	0.75926914
C	2.63042542	-1.13727041	0.25378	H	-1.07958262	0.88162194	-0.81493058
C	0.75061282	-2.55974497	-0.30543465	H	-0.69154624	-0.71967852	-1.49533854

Kekulene

C	-2.2494888	9.79598636	10.51360981	H	2.31254797	7.52044782	15.7189502

C	-1.95474575	10.09682711	11.85364102	C	2.82193456	6.23389199	14.07068853
H	-2.51081968	10.89019838	12.34822506	C	3.84811656	5.48330714	14.75413778
C	-0.96352409	9.41178748	12.57556727	C	4.53053935	4.47664556	14.14093734
C	-0.22862336	8.37544575	11.92050214	C	5.43034718	1.69848602	10.07598949
C	-0.52178681	8.09514919	10.58692521	H	6.19466694	1.14979665	10.6200354
H	0.05848574	7.33772768	10.08405754	C	5.1719468	1.3957497	8.77343996
C	-1.51130236	8.75885483	9.86349191	H	5.73012461	0.6055146	8.27825081
C	-1.80055722	8.42240848	8.47236065	C	0.81945208	3.90780041	4.5217717
C	-1.12373522	7.39930259	7.81040822	C	-0.18192743	4.62367853	3.84515441
H	-0.39260558	6.82940224	8.36168709	H	-0.39946835	4.3710599	2.80972578
C	-1.35917133	7.06030185	6.47901948	C	-0.90888947	5.6575651	4.45800791
C	-2.36145935	7.78246121	5.75990311	C	-0.61502724	5.99310613	5.81603305
C	-3.05948525	8.80747833	6.41949091	C	0.38960238	5.28392777	6.47236709
H	-3.82708697	9.35404318	5.8760645	H	0.63453519	5.56279295	7.48504603
C	-2.80381743	9.14798574	7.75805455	C	1.10909227	4.24756478	5.8796619
C	-3.51744401	10.19277462	8.45276537	C	2.14165029	3.51598581	6.60819885
C	-3.25900431	10.49554603	9.75532623	C	2.43488302	3.79633061	7.94176997
C	-0.64263716	9.71410581	13.9500603	H	1.85469598	4.55381573	8.44463319
H	-1.20057816	10.50436673	14.44547059	C	3.42435423	3.13259029	8.66519501
C	0.33207794	9.04308657	14.62439595	C	4.16246483	2.09539714	8.01512102
H	0.55185264	9.29800553	15.65781165	C	3.86773656	1.79454997	6.67511035
C	4.27436467	4.1088513	12.76884614	H	4.42378097	1.00115912	6.18052229
C	4.97235932	3.08378603	12.10925141	C	2.87653535	2.47962607	5.95314584
H	5.73990344	2.53717702	12.65271671	C	2.55568202	2.17735466	4.57865994
C	4.71672716	2.743348	10.77068656	C	1.58101263	2.84844293	3.90429628
C	3.7136044	3.46903995	10.05633701	C	-1.93514977	6.40809677	3.77455744
C	3.03686743	4.49222544	10.7182298	H	-2.15505442	6.15337084	2.74112037
H	2.30597475	5.06232892	10.16684166	C	-2.61766657	7.41465042	4.38779179
C	3.27217318	4.83111216	12.04968574	H	-3.3821092	7.96306364	3.84363351
C	2.52801919	5.89829751	12.71267112	H	1.36132265	2.59353038	2.87084346
C	1.52329285	6.60740328	12.05638857	H	3.11358716	1.38709035	4.08318782
H	1.27811743	6.3283615	11.04382245	H	-4.28183338	10.74150285	7.90882122
C	0.80391959	7.64386281	12.6490574	H	-3.81726676	11.28578055	10.25044812
C	1.0936275	7.98369308	14.00690373	H	4.06809186	5.73799829	15.78758496
C	2.09499543	7.26782697	14.6835186	H	5.29488585	3.92817145	14.68520043

Septulene

C	5.56652947	20.17865052	8.23364794	C	5.63119048	22.6050016	8.02400083
H	6.38708287	20.24934553	8.94200323	H	6.42288674	22.6050016	8.76924332
C	5.10680519	21.38615082	7.60402481	C	5.56652947	25.03135269	8.23364789
C	4.04558116	21.36773159	6.63674246	H	6.38708293	24.96065773	8.94200318
C	3.47181238	20.06311493	6.27242588	C	5.10680521	23.82385244	7.60402476
C	2.55640808	19.8533877	5.22413382	C	4.04558117	23.84227167	6.63674246
H	2.30765405	20.69127923	4.58167826	C	3.47181242	25.14688833	6.27242588
C	1.96985018	18.60101343	4.95788406	C	2.5564081	25.35661556	5.22413381
C	1.12670516	18.30007287	3.79026363	H	2.30765405	24.51872403	4.58167826
C	0.91685989	19.18142097	2.71157157	C	1.96985021	26.60898982	4.95788405
H	1.35801139	20.17144101	2.75574411	C	1.12670517	26.90993039	3.79026363
C	0.24884089	18.80376008	1.53214471	C	0.91685988	26.02858229	2.71157157
C	0.07962822	19.67337636	0.35794076	H	1.35801139	25.03856225	2.75574411
C	0.26122176	21.06848916	0.36398794	C	0.24884087	26.40624318	1.53214473
H	0.48939112	21.55104208	1.30829753	C	0.07962818	25.53662689	0.35794078
C	0.09084474	21.86924652	-0.78266805	C	0.26122175	24.1415141	0.36398795
C	-0.18122213	21.20011607	-2.02456071	H	0.48939114	23.65896118	1.30829753
C	-0.31841863	21.92981345	-3.25453884	C	0.09084472	23.34075674	-0.78266805
H	-0.46041561	21.36641717	-4.17243238	C	-0.18122217	24.00988719	-2.0245607
C	-0.74520793	17.68168179	-0.91619461	C	-0.31841865	23.28018981	-3.25453883
H	-1.080784	17.27723093	-1.86694653	H	-0.46041565	23.84358609	-4.17243237
C	-0.33302799	19.05754036	-0.87184583	C	-0.74520802	27.52832147	-0.91619457
C	-0.39114908	19.82391618	-2.03132414	H	-1.0807841	27.93277227	-1.86694649
H	-0.66094423	19.34036406	-2.96704694	C	-0.33302805	26.1524629	-0.87184581

C	0.5550631	16.98562537	3.6967011	C	-0.39114914	25.38608708	-2.03132411
C	0.75641349	16.00570754	4.72836541	H	-0.66094431	25.86963919	-2.96704692
H	0.26130706	15.04412485	4.62680024	C	0.55506311	28.22437788	3.6967011
C	1.59667867	16.25560551	5.75574081	C	0.75641352	29.20429572	4.72836541
H	1.80291633	15.50243627	6.51094999	H	0.26130709	30.16587841	4.62680024
C	2.25615709	17.52692274	5.8679112	C	1.59667872	28.95439774	5.75574076
C	3.56485362	22.6050016	6.1674867	H	1.8029164	29.70756704	6.51094993
H	2.72588024	22.60500166	5.47978456	C	2.25615713	27.68308052	5.8679112
C	4.93768028	19.00505037	8.0055172	C	4.93768033	26.20495289	8.00551714
H	5.23319273	18.09746106	8.52427172	H	5.23319281	27.11254219	8.52427167
C	3.86920924	18.92194176	7.048726	C	3.86920929	26.2880615	7.04872594
C	3.21585522	17.70825273	6.85946374	C	3.21585529	27.50175053	6.85946369
H	3.47727609	16.86651763	7.49612975	H	3.47727617	28.34348563	7.49612975
C	-0.12402756	16.61199232	2.54034452	C	-0.12402758	28.59801094	2.54034454
H	-0.51155449	15.59885813	2.46583817	H	-0.51155451	29.61114513	2.46583819
C	-0.24020091	17.45686232	1.44106494	C	-0.24020095	27.75314089	1.44106496
C	-0.76071679	16.93271257	0.20818598	C	-0.76071686	28.27729064	0.20818601
H	-1.11010724	15.90420261	0.19480883	H	-1.11010732	29.30580059	0.19480888

s

Octulene

C	10.59179554	2.5841012	8.48838488	C	6.69539054	9.44705196	7.00209405
C	8.35748876	1.76917207	6.95268128	C	1.80842927	7.16724027	7.67947564
C	3.44421019	0.69254878	7.00209405	H	2.72317358	6.58307526	7.6927863
C	8.33117145	2.97236047	7.67947564	C	0.69254544	6.69538593	8.39232603
H	7.41642714	3.55652549	7.6927863	C	1.39714871	4.27743662	8.63112769
C	9.44705529	3.44421479	8.39232603	H	2.16834483	4.43947743	7.88543185
C	8.74245203	5.86216413	8.63112769	C	2.95856618	9.01696876	6.35935542
H	7.97125588	5.70012328	7.88543185	C	1.76916604	1.78211017	8.44174108
C	7.18103454	1.12263198	6.35935542	C	7.16724323	8.33117245	7.71494459
C	8.37043471	8.35749055	8.44174108	H	6.5830787	7.41642745	7.70163684
C	2.97235749	1.80842825	7.71494459	C	7.55550243	10.59179734	6.90603399
H	3.55652202	2.72317328	7.70163679	C	0.59219489	5.36703541	9.0082566
C	2.5840983	-0.45219663	6.90603399	C	1.12262675	2.95856284	9.03506535
C	9.54740582	4.77256532	9.0082566	C	5.36703822	9.54740148	6.38616179
C	9.016974	7.18103788	9.03506535	C	3.83668044	10.83491761	4.98329751
C	4.77256254	0.59219924	6.38616179	H	3.65904809	11.67468942	4.31570545
C	6.3029203	-0.69531686	4.98329751	C	-0.53567143	8.66386915	7.64711762
H	6.48055262	-1.53508869	4.31570545	C	7.27371824	11.64021865	5.96266134
C	10.67527214	1.47573156	7.64711762	H	8.00060627	12.44011891	5.85207203
C	2.86588249	-1.50061793	5.96266134	C	0.52235352	9.04561529	6.82353065
H	2.13899445	-2.30051817	5.85207203	C	4.27743991	8.74244917	6.76329604
C	9.61724721	1.09398544	6.82353065	H	4.43948363	7.97125837	7.50899653
C	5.86216079	1.39715154	6.76329604	C	1.0939815	0.52235456	8.57088583
H	5.70011709	2.16834237	7.50899653	C	5.12334858	10.63301141	5.48069586
C	9.04561921	9.61724616	8.57088583	C	2.74396706	10.10523733	5.44964416
C	5.01625215	-0.4934107	5.48069586	C	-1.47869346	6.14078786	10.17146709
C	7.39563364	0.03436341	5.44964416	H	-2.25905671	5.92994348	10.89727501
C	11.6182942	3.99881288	10.17146709	C	-0.69532785	3.83667709	10.41111575
H	12.39865745	4.20965725	10.89727501	H	-1.53510372	3.65904451	11.07870365
C	10.83492857	6.30292364	10.41111575	C	-0.49341913	5.1233456	9.9137202
H	11.67470445	6.48055622	11.07870365	C	6.14079092	11.61828664	5.22294461
C	10.63301988	5.01625513	9.91372025	H	5.92994628	12.39864453	4.49713174
C	3.99880981	-1.47868591	5.22294461	C	1.47573014	-0.5356733	7.74729817
H	4.20965443	-2.25904382	4.49713173	C	-1.50062111	7.27371666	9.43175112
C	8.66387058	10.67527405	7.74729817	H	-2.30052142	8.00060389	9.54233725
C	11.64022183	2.86588406	9.43175112	C	1.41249557	10.56728765	5.15632301
H	12.44012214	2.13899681	9.54233731	H	1.2924121	11.3621249	4.42554733
C	8.72710515	-0.42768692	5.15632301	C	0.35781365	10.10946688	5.86979338
H	8.84718864	-1.22252417	4.42554733	H	-0.6359947	10.52651105	5.73248638
C	9.7817871	0.03013384	5.86979338	C	0.03435162	2.74396279	9.94477073

H	10.77559542	-0.38691032	5.73248638	C	0.03012297	0.35781163	9.52461447
C	10.10524913	7.39563793	9.94477073	H	-0.38692355	-0.63599678	9.66191385
C	10.10947778	9.78178911	9.52461447	C	-0.42770203	1.41249214	10.23808521
H	10.52652428	10.77559748	9.66191385	H	-1.22254464	1.29240792	10.96885492
C	10.56730278	8.72710859	10.23808521	H	0.890223	-1.45197054	7.7597213
H	11.36214537	8.84719282	10.96885492	H	11.59157435	0.89023211	7.63469349
C	-0.45219481	7.55549952	8.48838488	H	-1.45197361	9.24936859	7.63469349
C	1.78211198	8.37042867	6.95268128	H	9.24937774	11.59157128	7.7597213

Sumanene

C	1.2488099	8.95467814	4.09714346	C	1.20275996	6.43238928	2.97495965
C	0.08473702	8.20131887	4.09721978	H	3.69256352	9.79625925	2.20516512
C	0.00397291	6.94896074	3.4795143	H	4.16529729	9.82026257	3.9007771
C	-1.50439063	6.66753184	3.22695157	H	3.26675808	6.79947362	2.4552743
C	-2.15901209	8.05542904	3.4793709	H	1.22399826	5.47752281	2.4554351
C	-3.2793045	8.72520757	2.97462661	C	-1.1908647	8.85377732	4.09724398
C	-3.35203741	10.15478959	2.97475361	C	-1.26125678	10.23858433	4.09733652
H	-1.68398672	6.31639956	2.20539589	C	-2.30539325	10.93469107	3.47951934
H	-1.89942738	5.89530602	3.90109848	C	-1.79494566	12.3816998	3.22701056
H	-4.06606337	8.18378267	2.45495744	C	-0.26568265	12.25469382	3.4794826
H	-4.18949232	10.61384292	2.45507678	C	0.87451493	12.89007123	2.97482211
C	-0.05837707	11.01705217	4.09727596	C	2.14893057	12.23826869	2.97485865
C	1.17610235	10.38561761	4.09728444	H	-2.00919333	12.71280146	2.20544546
C	2.30100972	10.94183031	3.47946502	H	-2.26624423	13.10990707	3.90114357
C	3.2988796	9.77624307	3.22679914	H	0.79900363	13.84220456	2.45527928
C	2.42427597	8.51536651	3.47933333	H	2.96521203	12.7340847	2.45526551
C	2.40444632	7.21017453	2.97482207	C	1.20275996	6.43238928	2.97495965

Corannulene

C	1.01871207	3.72103349	4.98128197	C	-0.28634012	3.19387234	5.14193485
C	-1.42632461	3.86208754	4.72800542	C	1.26895772	4.95091612	4.3962416
C	2.55452651	5.52175615	4.74005544	C	2.97171037	3.79985465	6.47864356
C	3.36305584	4.97518283	5.72885692	C	1.32868739	1.99965671	8.09737454
C	3.39625488	3.33693677	7.78336912	C	-1.38939271	2.03800292	7.01534327
C	2.6160518	2.48212201	8.55198838	H	2.86484264	6.4556824	4.27786549
C	0.19518373	1.50420938	8.84996328	H	4.27388317	5.50328887	6.0007547
C	-1.09552315	1.52239196	8.33614529	H	4.3095379	3.73397151	8.21964226
C	-2.62481867	2.55617679	6.46578661	H	2.95007471	2.24452208	9.55915932
C	-2.64233975	3.42238604	5.3796901	H	0.33554909	1.18841785	9.88085416
C	-1.1667182	5.03935501	3.92592838	H	-1.91345989	1.22010216	8.9855151
C	1.84314188	3.16379653	5.989646	H	-3.56509942	2.33656041	6.96559691
C	0.11319644	5.55635937	3.76836345	H	-3.59562251	3.84586848	5.07299614
C	1.04762148	2.29211301	6.77340116	H	-2.00191985	5.59225261	3.50305307
C	-0.26846644	2.31068303	6.24949134	H	0.22813877	6.49314852	3.22856588

Data S1. CheckCIF validation report for compound 1, no A- and B- alters were observed. Crystallographic data have been deposited at the Cambridge Crystallographic Data Centre, under deposition no. CCDC 2496973.

Data S2. CheckCIF validation report for compound 5, no A- and B- alters were observed. Crystallographic data have been deposited at the Cambridge Crystallographic Data Centre, under deposition no. CCDC 2496972.

Data S3. CheckCIF validation report for compound 7, no A- and B- alters were observed. Crystallographic data have been deposited at the Cambridge Crystallographic Data Centre, under deposition no. CCDC 2496960.
