# Supplementary material for: Tetrabutoxy-substituted quintulene: A missing bowl-shaped cycloarene
Source: Sci Adv. 2026 Sep 2;12(36):eaee3923. doi: 10.1126/sciadv.aee3923 (PMC13537259; doi:10.1126/sciadv.aee3923)

checkCIF/PLATON report

Structure factors have been supplied for datablock(s) cxy5920_0ma

THIS REPORT IS FOR GUIDANCE ONLY. IF USED AS PART OF A REVIEW PROCEDURE FOR PUBLICATION, IT SHOULD NOT REPLACE THE EXPERTISE OF AN EXPERIENCED CRYSTALLOGRAPHIC REFEREE.

No syntax errors found. CIF dictionary Interpreting this report

Datablock: cxy5920_0ma

Bond precision:	C-C = 0.0040 A		Wavelength=1.34138
Cell:	a=21.136(2)	b=7.6784(8)	c=28.453(3)
	alpha=90	beta=102.958(6)	gamma=90
Temperature:	100 K		

	Calculated	Reported
Volume	4500.1(8)	4500.0(8)
Space group	P 21/n	P 1 21/n 1
Hall group	-P 2yn	-P 2yn
Moiety formula	C56 H52 O4, C H Cl3	C H Cl3, C56 H52 O4
Sum formula	C57 H53 Cl3 O4	C57 H53 Cl3 O4
Mr	908.34	908.34
Dx,g cm-3	1.341	1.341
Z	4	4
Mu (mm-1)	1.471	1.502
F000	1912.0	1912.0
F000'	1919.51	
h,k,lmax	26,9,35	26,9,35
Nref	9244	9227
Tmin,Tmax	0.694,0.729	0.688,0.745
Tmin'	0.417	

Correction method= # Reported T Limits: Tmin=0.688 Tmax=0.745
AbsCorr = MULTI-SCAN

Data completeness= 0.998 Theta(max)= 57.037

R(reflections)= 0.0756(7663) wR2(reflections)= 0.2491(9227)

S = 1.081 Npar= 679

The following ALERTS were generated. Each ALERT has the format
test-name_ALERT_alert-type_alert-level.
Click on the hyperlinks for more details of the test.

Alert level C

```
PLAT042_ALERT_1_C Calc. and Reported MoietyFormula Strings  Differ     Please Check
              Calc: C56 H52 O4, C H Cl3
              Rep.: C H Cl3, C56 H52 O4
PLAT051_ALERT_1_C Mu(calc) and Mu(cif) Ratio Differs from 1.0 by .       2.08 %
PLAT220_ALERT_2_C NonSolvent   Resd 1  C   Ueq(max)/Ueq(min) Range        4.6 Ratio
PLAT222_ALERT_3_C NonSolvent Resd 1  H   Uiso(max)/Uiso(min) Range        5.5 Ratio
PLAT242_ALERT_2_C Low    'MainMol' Ueq as Compared to Neighbors of        C27 Check
PLAT260_ALERT_2_C Large Average Ueq of Residue Including      Cl1A      0.129 Check
PLAT340_ALERT_3_C Low Bond Precision on  C-C Bonds ...............    0.00402 Ang.
PLAT410_ALERT_2_C Short Intra H...H Contact  H14      ..H55      .       1.99 Ang.
                                                     x,y,z  =      1_555 Check
PLAT910_ALERT_3_C Missing # of FCF Reflection(s) Below Theta(Min).          5 Note
                2  0  0,  -1  0  1,   1  0  1,  -2  0  2,   0  0  2,
PLAT911_ALERT_3_C Missing FCF Refl Between Thmin & STh/L=    0.600          2 Report
                3  1  2,   1  0  3,
PLAT918_ALERT_3_C Reflection(s) with I(obs) much Smaller I(calc) .          2 Check
```

Alert level G

```
ABSMU01_ALERT_1_G  Calculation of _exptl_absorpt_correction_mu
                not performed for this radiation type.
PLAT002_ALERT_2_G Number of Distance or Angle Restraints on AtSite         29 Note
PLAT003_ALERT_2_G Number of Uiso or U(i,j)  Restrained non-H-Atoms         27 Report
PLAT072_ALERT_2_G SHELXL First  Parameter in WGHT  Unusually Large       0.16 Report
PLAT171_ALERT_4_G The CIF-Embedded .res File Contains EADP Records          4 Report
PLAT172_ALERT_4_G The CIF-Embedded .res File Contains DFIX Records          4 Report
PLAT173_ALERT_4_G The CIF-Embedded .res File Contains DANG Records          2 Report
PLAT176_ALERT_4_G The CIF-Embedded .res File Contains SADI Records          7 Report
PLAT178_ALERT_4_G The CIF-Embedded .res File Contains SIMU Records          3 Report
PLAT188_ALERT_3_G A Non-default SIMU Restraint Value has been used     0.0100 Report
PLAT188_ALERT_3_G A Non-default SIMU Restraint Value has been used     0.0100 Report
PLAT188_ALERT_3_G A Non-default SIMU Restraint Value has been used     0.0100 Report
PLAT191_ALERT_3_G A Non-default SADI Restraint Value has been used     0.0400 Report
PLAT191_ALERT_3_G A Non-default SADI Restraint Value has been used     0.0400 Report
PLAT191_ALERT_3_G A Non-default SADI Restraint Value has been used     0.0400 Report
PLAT301_ALERT_3_G Main Residue  Disorder ..............(Resd    1)        15% Note
PLAT302_ALERT_4_G Anion/Solvent/Minor-Residue Disorder (Resd    2)       100% Note
PLAT302_ALERT_4_G Anion/Solvent/Minor-Residue Disorder (Resd    3)       100% Note
PLAT304_ALERT_4_G Non-Integer Number of Atoms in ..... (Resd    2)       4.28 Check
PLAT304_ALERT_4_G Non-Integer Number of Atoms in ..... (Resd    3)       0.72 Check
PLAT860_ALERT_3_G Number of Least-Squares Restraints ............         340 Note
PLAT883_ALERT_1_G Absent Datum for _atom_sites_solution_primary ..     Please Do !
PLAT912_ALERT_4_G Missing # of FCF Reflections Above STh/L=  0.600          9 Note
PLAT933_ALERT_2_G Number of HKL-OMIT Records in Embedded .res File          4 Note
               -1  0  1,   1  0  1,   1  0  3,   2  0  0,
PLAT969_ALERT_5_G The 'Henn et al.' R-Factor-gap value ..........      7.182 Note
              Predicted wR2: Based on SigI**2  3.47 or SHELX Weight 23.04
PLAT978_ALERT_2_G Number C-C Bonds with Positive Residual Density.          4 Info
```

```
 0 ALERT level A = Most likely a serious problem - resolve or explain
 0 ALERT level B = A potentially serious problem, consider carefully
11 ALERT level C = Check. Ensure it is not caused by an omission or oversight
26 ALERT level G = General information/check it is not something unexpected

 4 ALERT type 1 CIF construction/syntax error, inconsistent or missing data
 9 ALERT type 2 Indicator that the structure model may be wrong or deficient
13 ALERT type 3 Indicator that the structure quality may be low
10 ALERT type 4 Improvement, methodology, query or suggestion
 1 ALERT type 5 Informative message, check
```

It is advisable to attempt to resolve as many as possible of the alerts in all categories. Often the minor alerts point to easily fixed oversights, errors and omissions in your CIF or refinement strategy, so attention to these fine details can be worthwhile. In order to resolve some of the more serious problems it may be necessary to carry out additional measurements or structure refinements. However, the purpose of your study may justify the reported deviations and the more serious of these should normally be commented upon in the discussion or experimental section of a paper or in the "special_details" fields of the CIF. checkCIF was carefully designed to identify outliers and unusual parameters, but every test has its limitations and alerts that are not important in a particular case may appear. Conversely, the absence of alerts does not guarantee there are no aspects of the results needing attention. It is up to the individual to critically assess their own results and, if necessary, seek expert advice.

Publication of your CIF in IUCr journals

A basic structural check has been run on your CIF. These basic checks will be run on all CIFs submitted for publication in IUCr journals (*Acta Crystallographica*, *Journal of Applied Crystallography*, *Journal of Synchrotron Radiation*); however, if you intend to submit to *Acta Crystallographica Section C* or *E* or *IUCrData*, you should make sure that full publication checks are run on the final version of your CIF prior to submission.

Publication of your CIF in other journals

Please refer to the *Notes for Authors* of the relevant journal for any special instructions relating to CIF submission.

PLATON version of 02/02/2025; check.def file version of 02/02/2025

Datablock cxy5920_0ma - ellipsoid plot

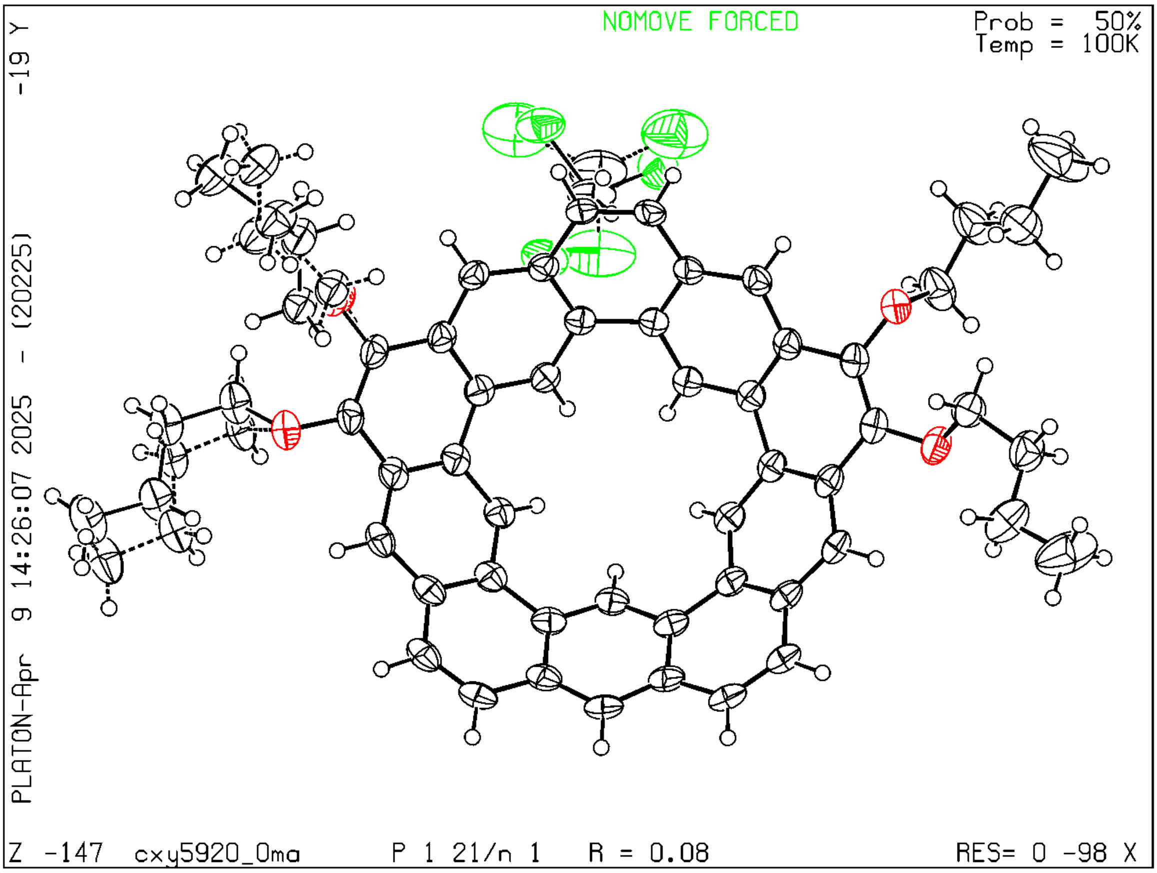

Supplement: Supplementary file 2 [file sciadv.aee3923_data_s1_to_s3.zip › Data S1. CheckCIF validation report for compound 1.pdf]
