# Supplementary material for: Tetrabutoxy-substituted quintulene: A missing bowl-shaped cycloarene
Source: Sci Adv. 2026 Sep 2;12(36):eaee3923. doi: 10.1126/sciadv.aee3923 (PMC13537259; doi:10.1126/sciadv.aee3923)

checkCIF/PLATON report

Structure factors have been supplied for datablock(s) 11

THIS REPORT IS FOR GUIDANCE ONLY. IF USED AS PART OF A REVIEW PROCEDURE FOR PUBLICATION, IT SHOULD NOT REPLACE THE EXPERTISE OF AN EXPERIENCED CRYSTALLOGRAPHIC REFEREE.

No syntax errors found.   CIF dictionary   Interpreting this report

Datablock: 11

Bond precision:	C-C = 0.0063 A		Wavelength=1.34139
Cell:	a=12.8818(9)	b=16.3007(12)	c=27.9818(19)
	alpha=74.260(3)	beta=89.853(3)	gamma=67.856(3)
Temperature:	100 K		

	Calculated	Reported
Volume	5205.4(7)	5205.4(6)
Space group	P -1	P -1
Hall group	-P 1	-P 1
Moiety formula	C56 H56 Br6 O4, C55 H53 Br6 O4, C H3 [+ solvent]	2(C56 H56 Br6 O4)
Sum formula	C112 H112 Br12 O8 [+ solvent]	C112 H112 Br12 O8
Mr	2544.82	2544.93
Dx,g cm-3	1.624	1.624
Z	2	2
Mu (mm-1)	3.922	3.923
F000	2536.0	2536.0
F000'	2517.05	
h,k,lmax	15,19,32	15,19,32
Nref	17763	17732
Tmin,Tmax	0.597,0.625	0.005,0.050
Tmin'	0.529	

Correction method= # Reported T Limits: Tmin=0.005 Tmax=0.050
AbsCorr = MULTI-SCAN

Data completeness= 0.998   Theta(max)= 52.096

R(reflections)= 0.0383(15699)

wR2(reflections)= 0.0975(17732)

S = 1.056

Npar= 1197

The following ALERTS were generated. Each ALERT has the format
test-name_ALERT_alert-type_alert-level.
Click on the hyperlinks for more details of the test.

Alert level C

```
THETM01_ALERT_3_C  The value of sine(theta_max)/wavelength is less than 0.590
            Calculated sin(theta_max)/wavelength =    0.5882
PLAT094_ALERT_2_C Ratio of Maximum / Minimum Residual Density ....       2.31 Report
PLAT241_ALERT_2_C High   'MainMol' Ueq as Compared to Neighbors of       C03L Check
PLAT242_ALERT_2_C Low    'MainMol' Ueq as Compared to Neighbors of       O00J Check
PLAT260_ALERT_2_C Large Average Ueq of Residue Including      C03P      0.171 Check
PLAT329_ALERT_4_C Carbon Atom Hybridisation Unclear for .........       C03O Check
PLAT341_ALERT_3_C Low Bond Precision on  C-C Bonds ...............    0.00632 Ang.
PLAT360_ALERT_2_C Short  C(sp3)-C(sp3) Bond  C03L     - C03M     .       1.41 Ang.
PLAT413_ALERT_2_C Short Inter XH3 .. XHn     H14      ..Hi       .       2.11 Ang.
                                                      x,y,z  =      1_555 Check
PLAT906_ALERT_3_C Large K Value in the Analysis of Variance ......      3.110 Check
PLAT911_ALERT_3_C Missing FCF Refl Between Thmin & STh/L=    0.588         30 Report
                0  0  2, -12-15  3,  12 14  3,  12 14  4,  -3 12  5, -13-10  6,
                0  0  6,  13 12  6,  12 14  6,  13 15  7,  13 10 10,  -7-13 13,
               10  9 14,  12 11 14,  -2 12 14,   8  9 15,   6 -3 16,   9  2 16,
                6 -4 17,   8 -2 17,   9  9 17,  -4 10 18,  -2 11 18,  -7  2 20,
               -4  8 20,  -3  9 20,  -6 10 21,  -6  9 23, -10  1 24,  -8  5 25,
PLAT971_ALERT_2_C Check Calcd Resid. Dens.  1.02Ang From Br0C            2.46 eA-3
PLAT971_ALERT_2_C Check Calcd Resid. Dens.  2.09Ang From C03P            1.53 eA-3
PLAT975_ALERT_2_C Check Calcd Resid. Dens.  0.74Ang From C03P    .       1.41 eA-3
PLAT976_ALERT_2_C Check Calcd Resid. Dens.  0.63Ang From C03O    .      -0.56 eA-3
```

Alert level G

```
ABSMU01_ALERT_1_G  Calculation of _exptl_absorpt_correction_mu
                not performed for this radiation type.
PLAT042_ALERT_1_G Calc. and Reported MoietyFormula Strings  Differ     Please Check
              Calc: C56 H56 Br6 O4, C55 H53 Br6 O4, C H3
              Rep.: 2(C56 H56 Br6 O4)
PLAT083_ALERT_2_G SHELXL Second Parameter in WGHT  Unusually Large      14.83 Why ?
PLAT154_ALERT_1_G The s.u.'s on the Cell Angles are Equal ..(Note)      0.003 Degree
PLAT333_ALERT_2_G Large Aver C6-Ring C-C Dist C00M     -C00Z     .       1.42 Ang.
PLAT333_ALERT_2_G Large Aver C6-Ring C-C Dist C00O     -C01D     .       1.42 Ang.
PLAT333_ALERT_2_G Large Aver C6-Ring C-C Dist C00Q     -C01I     .       1.42 Ang.
PLAT343_ALERT_2_G Unusual sp?      Angle Range in Main Residue for       C03O Check
PLAT344_ALERT_2_G Unusual sp?       Angle Range in Solvent/Ion for       C03P Check
PLAT367_ALERT_2_G Long?  C(sp?)-C(sp?) Bond  C03M     - C03O     .       1.59 Ang.
PLAT432_ALERT_2_G Short Inter X...Y Contact  C03M     ..C03P     .       2.97 Ang.
                                                      x,y,z  =      1_555 Check
PLAT432_ALERT_2_G Short Inter X...Y Contact  C03O     ..C03P     .       1.79 Ang.
                                                      x,y,z  =      1_555 Check
PLAT605_ALERT_4_G Largest Solvent Accessible VOID in the Structure         71 A**3
PLAT720_ALERT_4_G Number of Unusual/Non-Standard Labels .........         229 Note
              Br01     Br02     Br03     Br04     Br05     Br06     Br07     Br08
```

```
              Br09    Br0A    Br0B    Br0C    O00D    O00E    O00F    O00G
              O00H    O00I    O00J    O00K    C00L    C00M    C00N    C00O
              C00P    C00Q    C00R    C00S    H00S    C00T    C00U    C00V
              H00V    C00W    C00X    H00X    C00Y    C00Z    C010    C011
              C012    C013    C014    C015    C016    H016    C017    C018
              H018    C019    C01A    C01B    H01B    C01C    H01C    C01D
              C01E    C01F    H01F    C01G    H01G    C01H    H01H    C01I
              C01J    C01K    C01L    H01L    C01M    H01M    C01N    C01O
              C01P    C01Q    C01R    H01R    C01S    C01T    C01U    C01V
              H01V    C01W    H01W    C01X    C01Y    H01Y    C01Z    C020
              H020    C021    H021    C022    C023    C024    H024    C025
              C026    H02U    H02V    C027    H027    C028    C029    C02A
              H02A    H02B    C02B    C02C    H02W    C02D    C02E    C02F
              H02F    C02G    H02C    H02D    C02H    C02I    H02I    C02J
              H02E    H02G    C02K    H02H    H02J    C02L    H02X    H02Y
              C02M    H02Z    C02N    H02     Ha      C02O    Hb      C02P
              H02K    H02L    C02Q    H02M    H02N    C02R    C02S    H02O
              H02P    C02T    C02U    C02V    Hc      C02W    H02Q    H02R
              C02X    C02Y    H02S    H02T    C02Z    Hd      C030    H03Z
              He      C031    H03     Hf      C032    H03A    H03B    C033
              H03C    H03D    C034    Hg      C035    H03E    H03F    C036
              H036    C037    H03G    H03H    C038    Hh      C039    H03I
              H03J    C03A    H03K    H03L    C03B    H03M    H03N    H03O
              C03C    H03P    H03Q    C03D    H03R    H03S    H03T    C03E
              Hi      C03F    H03U    H03V    H03W    C03G    Hj      Hk
              C03H    Hl      C03I    Hm      Hn      C03J    H03X    H03Y
              Ho      C03K    Hp      Hq      C03L    Hr      C03M    Hs
              C03O    Ht      C03P    Hu      Hv
PLAT773_ALERT_2_G Check long C-C Bond in CIF: C03O     --C03P            1.79 Ang.
PLAT868_ALERT_4_G ALERTS Due to the Use of _smtbx_masks Suppressed          ! Info
PLAT909_ALERT_3_G Percentage of I>2sig(I) Data at Theta(Max) Still        75% Note
PLAT910_ALERT_3_G Missing # of FCF Reflection(s) Below Theta(Min).          1 Note
                0  0  1,
PLAT933_ALERT_2_G Number of HKL-OMIT Records in Embedded .res File          4 Note
              -11  0 23,   0  0  1,   0  0  2,   0  0  6,
PLAT969_ALERT_5_G The 'Henn et al.' R-Factor-gap value ...........      2.869 Note
              Predicted wR2: Based on SigI**2  3.40 or SHELX Weight  9.23
PLAT978_ALERT_2_G Number C-C Bonds with Positive Residual Density.          0 Info
```

---

0 **ALERT level A** = Most likely a serious problem - resolve or explain
0 **ALERT level B** = A potentially serious problem, consider carefully
15 **ALERT level C** = Check. Ensure it is not caused by an omission or oversight
21 **ALERT level G** = General information/check it is not something unexpected

3 ALERT type 1 CIF construction/syntax error, inconsistent or missing data
22 ALERT type 2 Indicator that the structure model may be wrong or deficient
6 ALERT type 3 Indicator that the structure quality may be low
4 ALERT type 4 Improvement, methodology, query or suggestion
1 ALERT type 5 Informative message, check

It is advisable to attempt to resolve as many as possible of the alerts in all categories. Often the minor alerts point to easily fixed oversights, errors and omissions in your CIF or refinement strategy, so attention to these fine details can be worthwhile. In order to resolve some of the more serious problems it may be necessary to carry out additional measurements or structure refinements. However, the purpose of your study may justify the reported deviations and the more serious of these should normally be commented upon in the discussion or experimental section of a paper or in the "special_details" fields of the CIF. checkCIF was carefully designed to identify outliers and unusual parameters, but every test has its limitations and alerts that are not important in a particular case may appear. Conversely, the absence of alerts does not guarantee there are no aspects of the results needing attention. It is up to the individual to critically assess their own results and, if necessary, seek expert advice.

Publication of your CIF in IUCr journals

A basic structural check has been run on your CIF. These basic checks will be run on all CIFs submitted for publication in IUCr journals (*Acta Crystallographica*, *Journal of Applied Crystallography*, *Journal of Synchrotron Radiation*); however, if you intend to submit to *Acta Crystallographica Section C* or *E* or *IUCrData*, you should make sure that full publication checks are run on the final version of your CIF prior to submission.

Publication of your CIF in other journals

Please refer to the *Notes for Authors* of the relevant journal for any special instructions relating to CIF submission.

PLATON version of 11/11/2024; check.def file version of 11/11/2024

Datablock 11 - ellipsoid plot

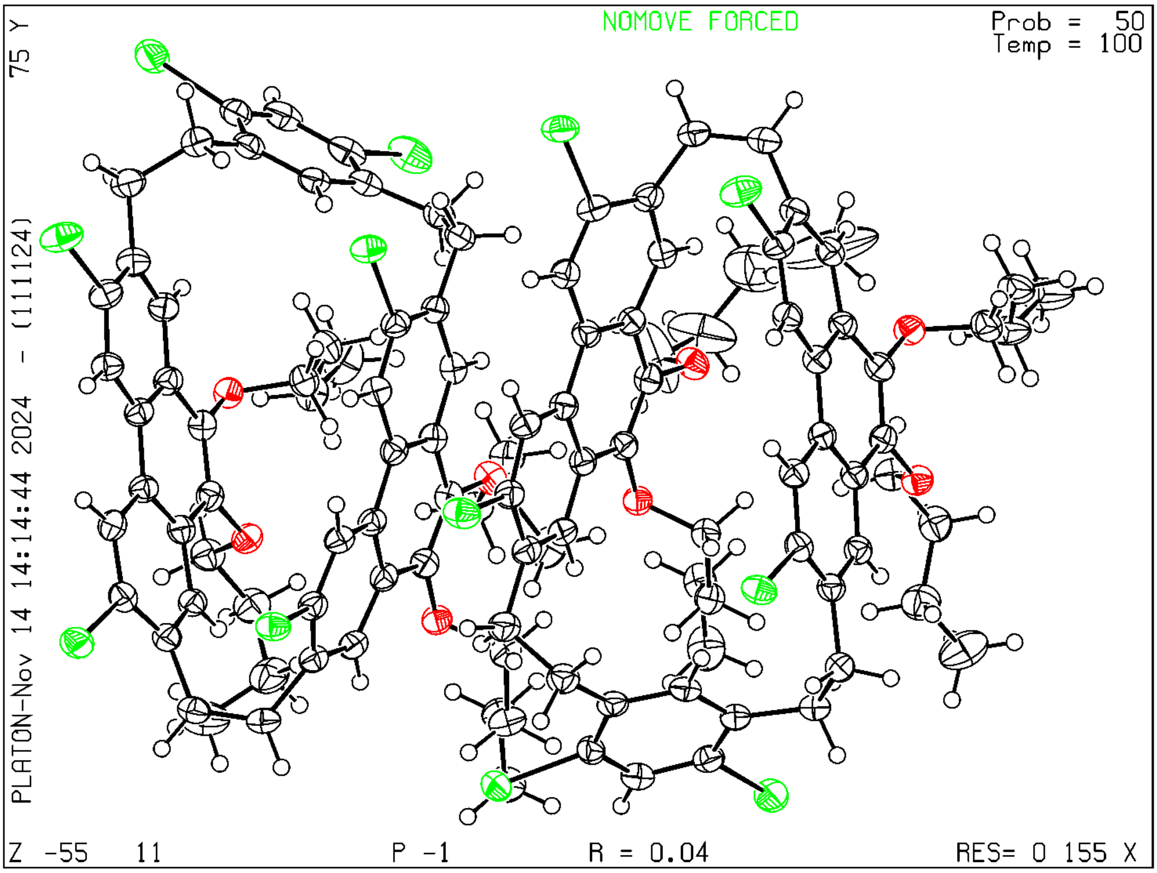

Supplement: Supplementary file 2 [file sciadv.aee3923_data_s1_to_s3.zip › Data S3. CheckCIF validation report for compound 7.pdf]
